# Coupling Rotary Motion to Helicene Inversion within a Molecular Motor

**DOI:** 10.1002/anie.202416097

**Published:** 2024-11-26

**Authors:** Yohan Gisbert, Marco Ovalle, Charlotte N. Stindt, Romain Costil, Ben L. Feringa

**Affiliations:** ^1^ Stratingh Institute for Chemistry University of Groningen Nijenborgh 3 9747 AG Groningen, The Netherlands

**Keywords:** Chirality, Molecular motors, Coupled motion, Photochemistry, Isomerization

## Abstract

Towards complex coupled molecular motions, the remote handedness inversion of a helicene moiety was achieved by a rotary molecular motor. The use of a specifically engineered dynamic helicene stator in a novel overcrowded‐alkene second‐generation molecular motor based on a fluorinated dibenzofluorene fragment allows for an unprecedented control over helicity inversion. This is achieved by the mechanical coupling of the rotation of the rotor to the helicene inversion of the stator half via a remote chirality transmission process. Thus, the unidirectional rotary motion generated upon irradiation is used to invert the dynamic stereochemistry of a helicene, leading to a 6‐step cycle with eight intermediates. In this cycle, both alternation between *P* and *M* configurations of the helicene stator and dynamic thermal interconversion (paddling motion) can be achieved. In‐depth computational and spectroscopic studies were performed to support the associated mechanism. The control over coupled motion and dynamic helicity offers prospects for the development of complex responsive systems.

## Graphical abstract

A light‐powered molecular motor featuring a specifically engineered helicene moiety is presented. The inversion of the helicene fragment is mechanically coupled to the unidirectional rotation of the motor in a 6‐step rotation cycle, which involves 8 isomers. This coupled motion results in the dynamic modulation of the handedness of the helicene by the motor via a central to helical to helical chiral transmission mechanism.

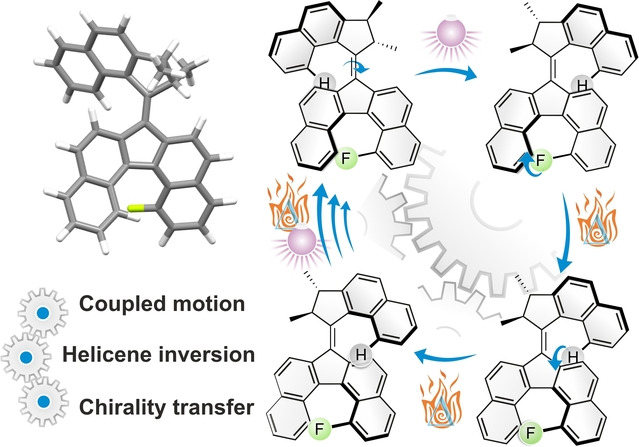

## Introduction

Since their advent, artificial molecular motors[^1^, ^2^] and other molecular machines[^3^, ^4^, ^5^, ^6^] have become ubiquitous for controlling function[^7^, ^8^, ^9^] and motion[^10^, ^11^, ^12^, ^13^, ^14^] at the molecular scale. Responsive systems such as pumps,[^15^, ^16^, ^17^, ^18^] linear muscle‐like actuators,[^19^, ^20^, ^21^, ^22^, ^23^] transporters,[^24^, ^25^] adaptive catalysts,[^26^, ^27^] and release systems[^28^, ^29^] have been reported in recent years. Among these examples, rotary molecular motors[^5^, ^7^] hold a special place, as these artificial nanometric devices can convert an input energy into a controlled, directional and repetitive motion,^[1]^ ultimately leading to the production of useful work.^[5]^ Various strategies have been implemented to harness the output force generated along with the rotation.[^7^, ^19^, ^30^] The most frequent approach involves the design of systems exploiting the collective motion of networks of motors ultimately resulting in a transmission of the generated movement across length scales.[^31^, ^32^, ^33^, ^34^] Apart from this materials‐oriented approach, the direct geared transmission of motion at the (sub−)molecular scale remains challenging but could serve as a significant step towards more complex motions and molecular machine type functions.^[10]^ To achieve this, methods have been devised, relying for instance on the preparation of molecular gears,[^35^, ^36^] aiming to be assembled into trains[^37^, ^38^] or into higher order systems.^[39]^ These systems could potentially be further coupled to a powered motion source such as a molecular motor.^[40]^

Taking advantage of directional rotation to control intramolecularly coupled motion^[41]^ has recently drawn significant attention as a way to increase the functionality of molecular motors and make use of the generated motion.^[42]^ Secondary motions were coupled to the rotation of motors, such as additional rotation,^[43]^ oscillation,[^44^, ^45^, ^46^, ^47^] winding[^48^, ^49^] and threading.[^50^, ^51^] Coupled motions are highly interesting for the design of functional molecular systems and open the way to a range of new applications, being a unique way to propagate the produced rotative motion, convert it into another dynamic function or enable operations similar to those of macroscopic machines.

**Figure 1:**
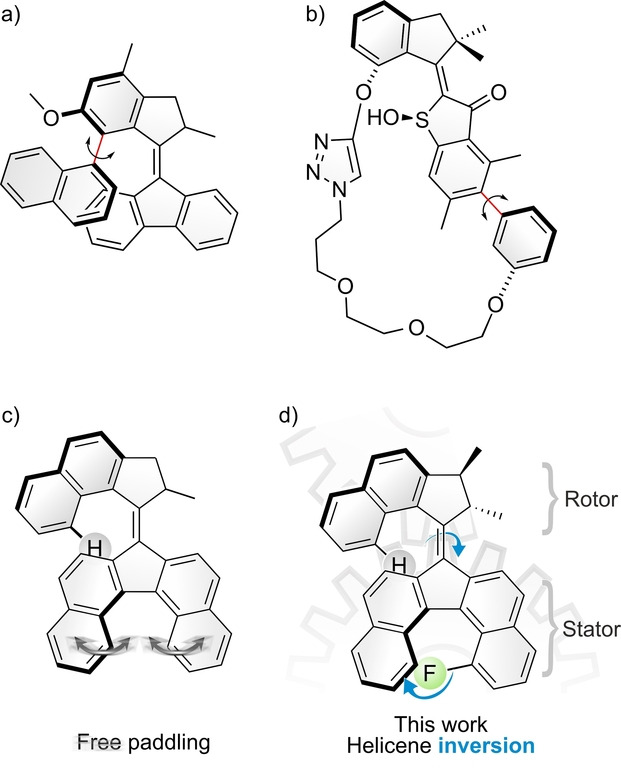
Examples of second‐generation molecular motors displaying coupled motions: a) synchronous rotation,^[40]^ b) biaryl atropoisomerisation,[^52^, ^53^, ^54^, ^55^, ^56^] c) uncontrolled paddling motion,^[57]^ d) this work: helicene inversion.

Second‐generation overcrowded‐alkene‐based molecular motors have been used as an ideal platform to study the coupling of rotary motion with more complex mechanical phenomena. Notably, we have achieved the remote modulation of the axial chirality of another submolecular fragment by synchronous rotation, affording coupled atropoisomerisation (Figure 1a).^[40]^ Coupled rotation and atropoisomerisation of biaryl moieties was also accomplished by Dube and co‐workers[^52^, ^53^, ^54^, ^55^, ^56^] using macrocycle‐embedded molecular motors (Figure 1b). These examples combined the actuation of additional dynamic stereoelements with the usual rotation of molecular motors, thus harnessing the motion generated by the isomerization of the central double bond to perform other tasks in the form of coupled motion and stereochemical modulation.

Recently, an overcrowded‐alkene‐based molecular motor featuring a dibenzo[*c,g*]fluorene stator has been conceived by our group.^[57]^ This refined design (Figure 1c) displayed improved properties compared to previous generations of molecular motors such as a bathochromic shift of the excitation wavelength resulting from the extension of the aromatic system. Unidirectional rotation of this molecular motor was obtained with visible light of up to 490 nm. Another unusual feature of this particular molecular motor is the inherent helicity of the dibenzo[*c*,*g*]fluorene stator, but applications are limited due to the very low racemization barrier of this helicene stator moiety. In this early work, a theoretical study based on DFT calculations showed that each photochemical and thermal step in the rotary cycle induced an inversion of the helicity of the dibenzo[*c,g*]fluorene stator half, thus giving rise to a paddling motion along with the rotation around the alkene axis. This phenomenon could unfortunately not be confirmed experimentally due to the low racemization barrier.

We envisioned that we could use such coupled motion to modulate the remote dynamic stereochemistry within the system via the inversion of helicene chirality in a fully reversible way using light as a stimulus. Such dynamic modulation of the handedness of a helicene within a small molecule through a straightforward photochemical activation has, to the best of our knowledge, no precedent. Noting the importance of intrinsically chiral arene systems, in particular helicenes,[^58^, ^59^] achieving selective control of helicene handedness could be of the highest importance for the development of new responsive chiral materials, as emphasized by Crassous et al.[^60^, ^61^]

Here, we successfully achieved chirality transfer and coupled motion between the two halves of the molecular motor experimentally by developing a motor‐helicene hybrid depicted in Figure 1d. Our newly designed motor bears a fluorine atom within the fjord region of the helicene stator half, that allows for a fine‐tuning of the helicity inversion barrier, thus enabling to decorrelate this unusual phenomenon of coupled motion from the thermal fluctuations leading to handedness inversions of the helicene fragment in a fully dynamic way.

## Results and Discussion

### Rational Design of M1

**Scheme 1:**
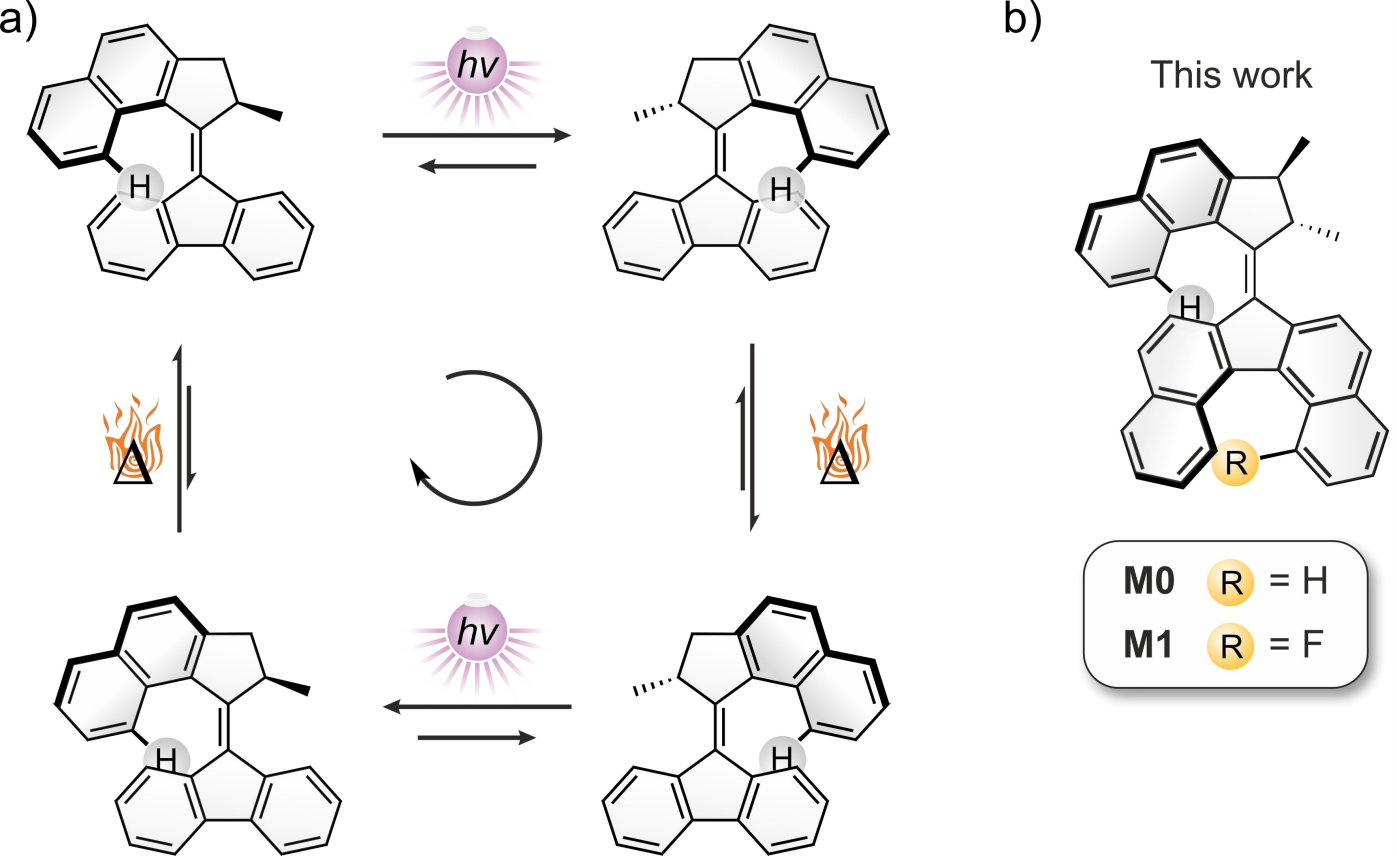
Rotation cycle of the “archetypical” 2^nd^ generation molecular motor featuring four intermediates. b) Structures of the molecular motors considered for the DFT study featuring two point‐chiral centers, as well as three dynamic stereoelements: two helices and the central overcrowded‐alkene.

Second‐generation light‐driven molecular‐motors can generate a controlled unidirectional rotational motion by following a mechanistic cycle composed of four steps for a complete 360° revolution. This mechanism (Scheme 1a) involves successive photoinduced *E*/*Z* isomerizations of the central overcrowded‐alkene double bond, each followed by a Thermal Helix Inversion step (THI). For our new design, we reasoned that the most straightforward strategy to show the helicene inversion coupled to the rotation of the overcrowded‐alkene‐based motor would involve the synthesis of a dibenzo[*c*,*g*]fluorene moiety functionalized within its fjord region. The steric hindrance provided by this functional group was envisioned to induce an increase of the racemization barrier of the stator, thus allowing for an experimental study of this coupled rotation–helicene inversion motion, as well as a differentiation of every step constituting the rotation mechanism. Given that this compound was designed with the objective of demonstrating remote chirality transfer via a central to helical to helical chiral transmission mechanism, we decided to slightly adapt the design of the rotor (upper half) of the motor compared to our earlier design^[57]^ (Figure 1c) by adding an additional methyl group on the rotor. This allowed for the selective asymmetric synthesis of both enantiomers of the desired motor, taking advantage of our recently reported method.[^62^, ^63^] It was previously shown that molecular motors with a single methyl group in the rotor racemize during the Barton–Kellogg coupling that is used to connect the rotor and stator parts whereas epimerization of the α,β‐dimethylated rotor is prevented.

**Scheme 2:**
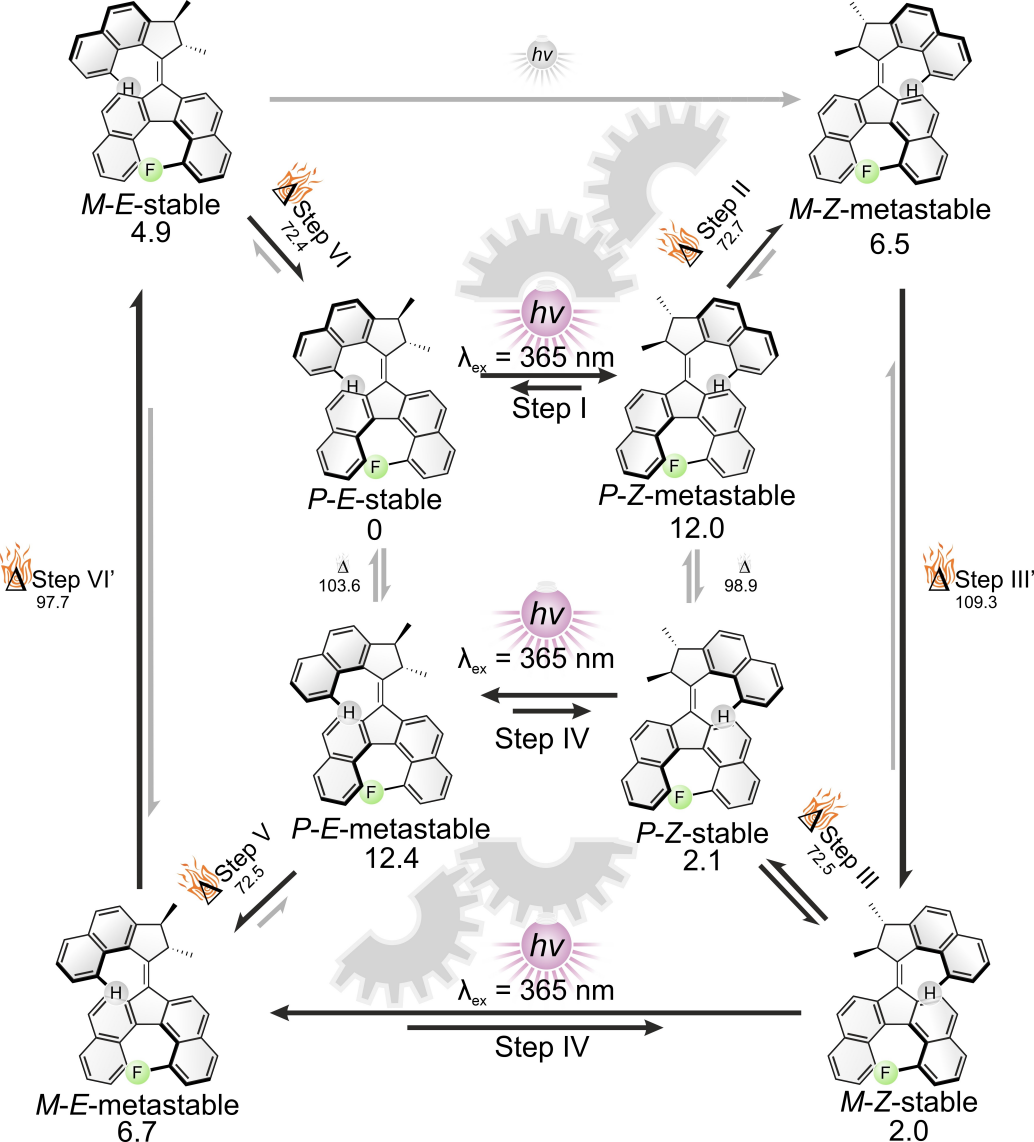
Calculated rotation cycle for **M1**. Calculations were performed at the r^2^SCAN‐3c/CPCM(toluene) level of theory. Gibbs free energy values are given in kJ/mol at 25 °C. Gibbs free energy values of the THI barriers are given with respect to the corresponding metastable state. The stereochemistry shown corresponds to the (2*S*,3*S*)‐**M1** compound. The *P*/*M* annotation denotes the helicity of the helicene stator. Gears highlight the steps involved in the coupled motion phenomenon.

The target design (Scheme 1b) therefore displays five different stereochemical elements: two point‐chiral centers, two helices and the central alkene double bond. As the stereochemistry of both stereogenic methyl groups is fixed, the rotation cycles of each enantiomer of the motor involve eight stereoisomers (Scheme 2) arising from the possible helicities for both *E* and *Z* isomers.

We performed DFT calculations to estimate the influence of substitution on the free energy of isomerization of the stator (i.e. barrier of helicene inversion) and more importantly its relative energy compared to the free energy of the THI of the rotor. In order to be able to experimentally decorrelate the THI of the rotor moiety from the helicene inversion of the stator moiety, both processes should have sufficiently different activation barriers, each high enough to prevent the process under cryogenic conditions. Geometries of the isomers and transition states involved in the rotational cycles were computed at the r^2^SCAN‐3c^[64]^ level of theory using the conductor‐like polarizable continuum CPCM(toluene) solvent model.^[65]^ **M1** (R=F, Scheme 1b) was shown to be the best candidate. The lowest‐energy barriers for the THI of **M1** were calculated to be 72.4 kJ/mol for the stator and 97.7 kJ/mol (Scheme 2) for the rotor part instead of 37.6 kJ/mol and 103.5 kJ/mol, respectively, for the unsubstituted analogue **M0** (R=H, Scheme 1b, Figures S29, S30 and S31). When R=F, both THI processes should now require thermal activation, thus allowing the rotation process to be paused at a chosen step at cryogenic temperatures in order to thoroughly characterize every intermediate with low‐temperature (chir−)optical and NMR spectroscopies. Moreover, the fluorine substituent can be used as a spectroscopic handle, allowing to clearly distinguish all isomers by ^19^F NMR due to geometrical changes around the fluorine atom in the fjord region of the bottom half of the motor.

Compared to classical second‐generation motors (Scheme 1a), **M1** features a more complicated rotation cycle composed of 6 steps and eight intermediates instead of four steps and four intermediates.^[66]^ This duplication results from the two possible helicities of the stator at each step of the rotation cycle. In Scheme 2, rotation intermediates displaying a *P* helicity of the stator are represented in the central cycle and *M* helicities in the outer one. The relative stability of the *P* or *M* helicity of the dibenzofluorene half is biased by the conformation of the rotor, resulting in multiple controlled helix inversions of the stator along the most energetically favored mechanistic pathway.

### Asymmetric Synthesis of (2R,3R)‐M1 and (2S,3S)‐M1

**Scheme 3:**
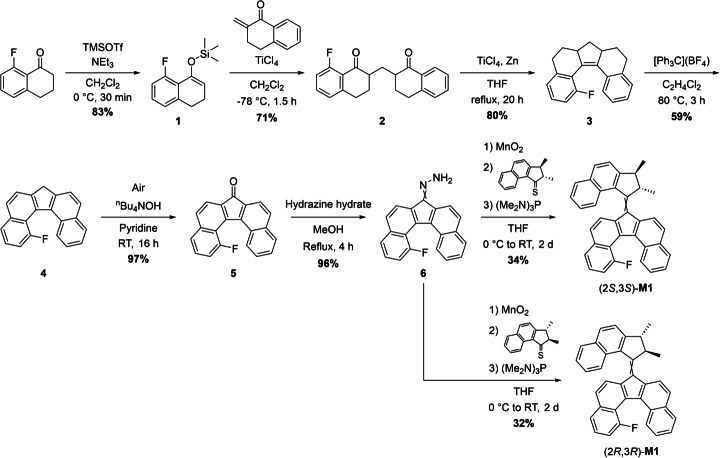
Asymmetric synthesis of motors (2*R*,3*R*)‐**M1** and (2*S*,3*S*)‐**M1**. Both were obtained as mixtures of the *E* and *Z* isomers in varying ratios depending on the handling conditions.

The fluorinated dibenzo[*c*,*g*]fluorenone stator half **5** (Scheme 3) was prepared in seven steps starting from 8‐fluoro‐1‐tetralone^[67]^ following a synthetic pathway adapted from the synthesis of symmetrical dibenzo[*c*,*g*]fluorenes.^[68]^ The dissymmetric 1,5‐diketone **2**, was synthesized by successive methylenation of α‐tetralone according to a literature procedure,^[69]^ followed by a Mukaiyama‐Michael addition of the silyl enol ether **1** obtained by treatment of 8‐fluoro‐1‐tetralone with triethylamine and trimethylsilyl trifluoromethanesulfonate. The obtained dissymmetric 1,5‐diketone **2** was then subjected to an intramolecular McMurry coupling, affording the hydrogenated fluorene analogue **3**. It must be noted that intermediates **2** and **3** both display two stereogenic centers and were isolated as diastereomeric mixtures. The hydrogenated fluorene analogue **3** was then oxidized with tritylium tetrafluoroborate, yielding 1‐fluoro‐dibenzo[*c*,*g*]fluorene **4**. The latter was further oxidized with ambient oxygen by stirring a solution of **4** in pyridine in an open flask in the presence of a catalytic amount of tetrabutylammonium hydroxide to provide the fluorine‐substituted dibenzofluorenone bottom half **5**. Fluorenone **5** was subsequently reacted with hydrazine hydrate to form the corresponding hydrazone **6**, which was oxidized by manganese dioxide to generate the corresponding diazo species. This diazo compound was then in situ subjected to a Barton–Kellogg coupling with either (2*R*,3*R*)‐ or (2*S*,3*S*)‐2,3‐dimethyl‐2,3‐dihydro‐1*H*‐cyclopenta[*a*]naphthalen‐1‐thione yielding highly enantioenriched molecular motors (2*R*,3*R*)‐**M1** or (2*S*,3*S*)‐**M1** as mixtures of the *Z* and *E* isomers in varying ratios depending on the handling conditions.

**Figure 2:**
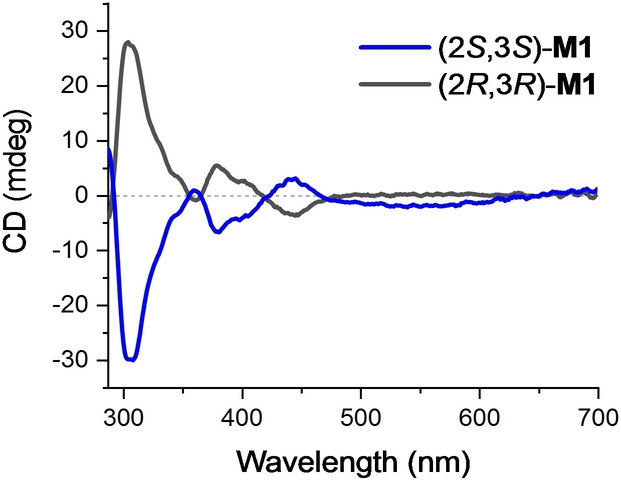
CD spectra of (2*R*,3*R*)‐**M1** (black) and (2*S*,3*S*)‐**M1** (blue) (toluene, ∼50 μM, 20 °C). Both samples are composed of isomeric mixtures (*E*/*Z* and stable/metastable isomers) as synthesized.

Both enantiomers of **M1** were obtained in ≥75 % *ee*, identical to the ones of the parent enantioenriched rotors.[^62^, ^63^] Due to opposite point chirality and helicities, opposite CD spectra displaying intense Cotton effects at 305 nm were obtained (Figure 2).

### Experimental Study of the Rotation and Coupled Selective Remote Helicene Inversion

**Scheme 4:**
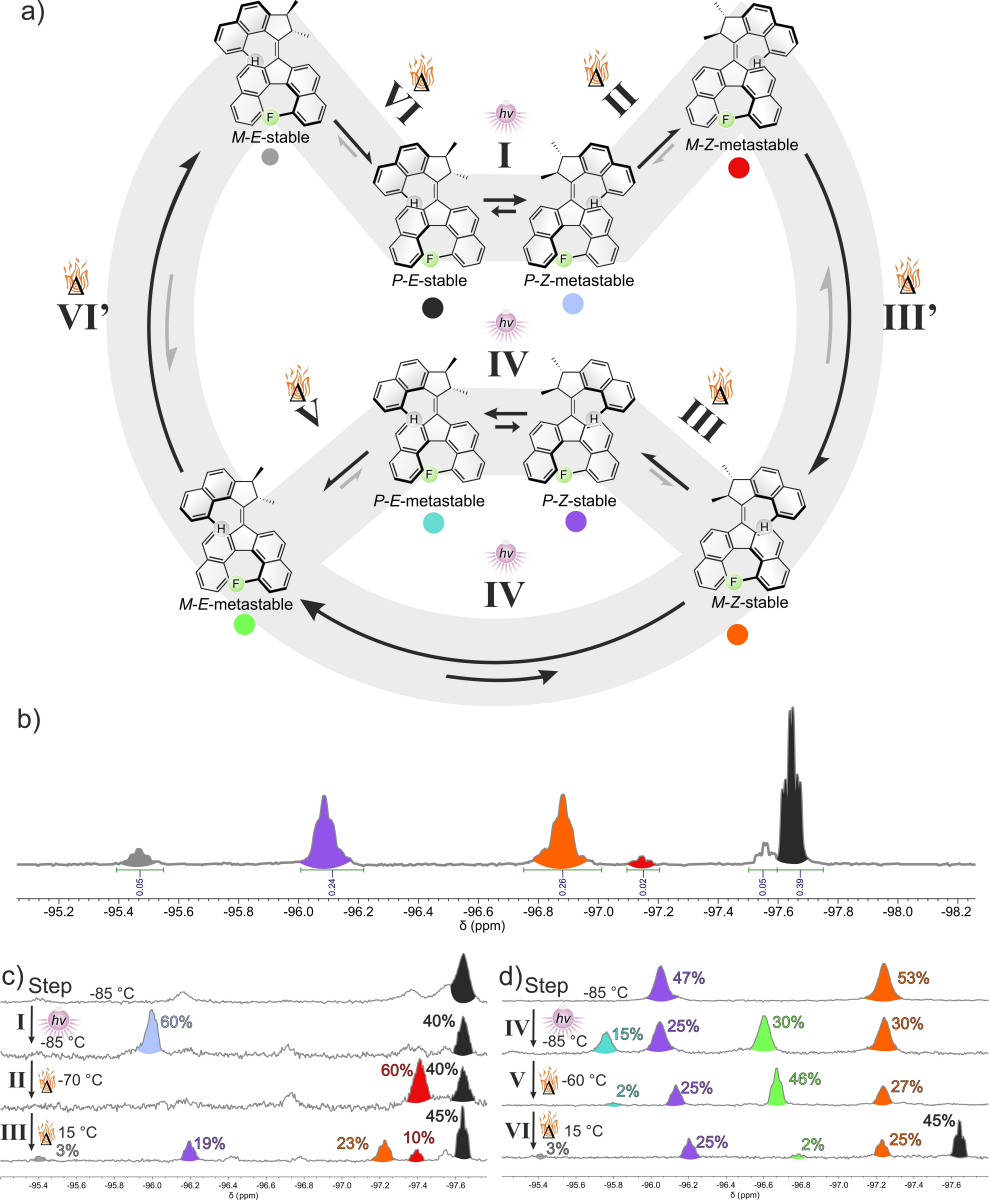
Characterization of the rotation cycle. a) Observed rotation mechanism involving eight intermediates. b) ^19^F NMR spectrum (500 MHz, 25 °C, toluene‐d8) of a mixture of *Z* and *E* isomers. Minor signals were assigned to less stable diastereomers. c) Conversion of *P*‐*E*‐stable **M1** into a mixture of *M*‐ and *P*‐*Z*‐stable **M1** by irradiation with 365 nm light followed by two successive THI steps. d) Conversion of a mixture of *M*‐ and *P*‐*Z*‐stable **M1** into *P*‐*E*‐stable **M1** by irradiation with 365 nm light followed by two successive THI steps.

As previously stated, **M1** was obtained as a mixture of the most stable isomers, mainly composed of the *P*‐*E*‐stable, *P*‐*Z*‐stable and *M*‐*Z*‐stable isomers (Scheme 4a). These isomers are easily distinguishable by ^19^F NMR (Scheme 4b) and are present in a distribution that is in accordance with the theoretically calculated relative energies (Scheme 2).

In order to prove that **M1** behaves as suggested by the calculations, we set out to independently study all steps involved in the rotation cycle presented in Scheme 2. To accomplish this, *Z* and *E* isomers of the molecular motor **M1** were first separated by column chromatography, affording highly enriched (ca. 75–100 % isomeric purity) analytical samples of both isomers.

#### Step I. P‐E‐Stable→P‐Z‐Metastable–Photoisomerization (Scheme 4, Step I)

The purified *P*‐*E*‐stable isomer features one major ^19^F NMR signal at −97.6 ppm at −85 °C. The minor signals correspond to residual *Z*‐isomers and less‐stable isomers being in a thermal equilibrium (Scheme 4c). Upon irradiation with 365 nm light at −85 °C the *P*‐*E*‐stable isomer is converted into the *P*‐*Z*‐metastable isomer (−96.0 ppm, step I), reaching a 54 : 46 *P*‐*E*‐stable*/P*‐*Z*‐metastable isomer ratio in the photostationary state (PSS) (Scheme 4c). UV/Vis spectroscopy at −85 °C showed a decrease of the most intense absorption band at λ_max_=413 nm and the formation of a redshifted absorption band at 472 nm with a well‐defined isosbestic point (Figure S15), in line with the photoswitching of a second‐generation molecular motor. This process was further followed by CD spectroscopy at the same temperature, displaying sign inversions of the Cotton effect at λ=310 and 465 nm, as expected from the helix inversion of the rotor upon photoisomerization (Figure S24).

#### Step II. *P*‐*Z*‐Metastable→*M*‐*Z*‐Metastable—Thermal Helicene Inversion (Scheme 4, Step II)

Increasing the temperature to −70 °C resulted in the complete conversion of the *P*‐*Z*‐metastable isomer into the *M*‐*Z*‐metastable isomer via an inversion of the helical stator, illustrating the coupled motion phenomenon. This change was observed by ^19^F NMR with the disappearance of the signal at −96.0 ppm and the appearance of a new upfield shifted signal at −97.4 ppm (Scheme 4c). Only minor changes were observed by UV/Vis spectroscopy, while CD spectroscopy highlighted the helix inversion process of the stator with a sign inversion of the Cotton effect at 302 nm (Figure S25).

#### Step III. *M*‐*Z*‐Metastable→*M*‐*Z*‐Stable+*P*‐*Z*‐Stable—Thermal Helix and Helicene Inversion (Scheme 4, Step III and III’)

Raising the temperature to 15 °C allowed for the relaxation of the *M*‐*Z*‐metastable state to a mixture of the *M*‐*Z*‐stable and *P*‐*Z*‐stable isomers, both having similar energies and being in a thermal equilibrium with a barrier (ΔG^≠^_calc step III_=72.5 kJ/mol) which is lower than the one involved in the thermal relaxation of the *M*‐*Z*‐metastable isomer (ΔG^≠^_calc step III’_=109.3 kJ/mol) (Scheme 2).

This transformation resulted in the conversion of the ^19^F NMR signal of *M*‐*Z*‐metastable at −97.4 ppm into two new signals at −96.2 and −97.2 ppm (Scheme 4c) which correspond to the *P‐Z‐*stable and *M‐Z‐*stable isomers, respectively. The assignment of these signals was based on the best‐fitting kinetic profile (Figure S6). The THI process was also followed by UV/Vis spectroscopy at 35 °C which showed a decrease in absorption at λ=465 nm and an increase at 405 nm. Meanwhile, CD spectroscopy showed sign inversions of the Cotton effects, at 320 and 378 nm (Figure S26).

This step indeed does not display coupled motion but a free paddling of the stator half. The exchange dynamics between these two species were further studied by variable‐temperature exchange spectroscopy (VT‐EXSY), allowing to experimentally determine the helicene inversion barrier of the stator (i.e. interconversion of *M*‐*Z*‐stable and *P*‐*Z*‐stable). The barrier for both the forward and backward isomerization was determined to be ΔG^≠^_exp step III_=66 kJ/mol, close to the calculated value (see Supporting Information for detailed discussion of the method).

In order to simplify analysis, the second half of the rotation cycle (Scheme 4, step IV to step VI) was studied starting from pure samples of the *Z*‐isomers obtained by column chromatography and composed solely of the *P*‐*Z*‐stable and *M*‐*Z*‐stable isomers, which displayed two characteristic ^19^F NMR signals at −96.0 and −97.2 ppm at −85 °C (Scheme 4d).

#### Step IV. *P*‐*Z*‐Stable+*M*‐*Z*‐Stable→*P*‐*E*‐Metastable+*M*‐*E*‐Metastable—Photoisomerization and Partial Thermal Helicene Inversion (Scheme 4, Step IV)

The mixture was cooled down to a −85 °C at which the interconversion between to the two *Z*‐stable isomers (step III) was inhibited and subsequently irradiated with 365 nm light. The conversion of the *Z*‐stable isomers (step IV) was monitored by ^19^F NMR and resulted in the generation of two metastable states *P*‐*E*‐metastable and *M*‐*E*‐metastable with characteristic signals at −95.8 and −96.6 ppm, respectively (Scheme 4d). At this temperature, the slow thermal helicene inversion (step V) from the *P*‐*E*‐metastable state to the *M*‐*E*‐metastable state was also observed as shown by the fitting model in Figure S5. Similarly to the previous photoisomerization, UV/Vis spectroscopy at −85 °C revealed a bathochromic shift of the maximum absorption wavelength (λ_max_) from 425 nm to 443 nm (Figure S19). However, a single well‐defined isosbestic point was not observed due to the two isomerizations taking place simultaneously. CD spectroscopy also showed inversion of the Cotton effect at 320 and 440 nm, in line with the helix inversion of the rotor of both isomers (Figure S27).

#### Step V. *P*‐*E*‐Metastable→*M*‐*E*‐Metastable—Thermal Helicene Inversion (Scheme 4, Step V)

The *P*‐*E*‐metastable isomer was fully converted to the *M*‐*E*‐metastable state via the helicene inversion of the stator by heating the sample to −65 °C, resulting in the disappearance of the signal at −95.8 ppm and the increase of the one at −96.6 ppm (Scheme 4d). Negligible changes were observed by UV/Vis spectroscopy. CD spectroscopy mainly featured a decrease of the most intense Cotton effect at 299 nm. No sign inversion was witnessed from this helicene inversion step, likely due to the presence of the *M*‐*E*‐metastable isomer before this thermal relaxation, thus resulting in a further decrease of the signal without inversion (Figure S28).

#### Step VI. *M*‐*E*‐Metastable → *P*‐*E*‐Stable—Thermal Helix and Helicene Inversion (Scheme 4, Step VI and VI’)

Finally, the *P*‐*E*‐stable isomer can be regenerated following two successive thermal relaxation steps, involving at first a THI of the rotor to yield the intermediate *M*‐*E*‐stable (step VI’) state which directly relaxes to the more stable initial *P*‐*E*‐stable isomer by a helicene inversion, following a coupled motion mechanism (step VI). As the second energy barrier ΔG^≠^_calc step VI_=72.4 kJ/mol is significantly lower than the first one (ΔG^≠^_calc step VI’_=97.7 kJ/mol), the thermal decay of *M*‐*E*‐metastable into *M*‐*E*‐stable cannot be studied separately from the relaxation to *P*‐*E*‐stable. Hence, the overall transformation resulted in the conversion of the signal corresponding to *M*‐*E*‐metastable at −96.8 ppm into a single peak assigned to the *P*‐*E*‐stable isomer (Scheme 4d) at 15 °C. A significant hypsochromic shift of the maximal absorption wavelength from λ_max_=440 to 417 nm was observed by UV/Vis spectroscopy (Figure S22) while sign changes of the Cotton effect at λ=320 and 400 nm with the concomitant disappearance of the band at 475 nm were observed by CD spectroscopy (Figure S29). As indicated before, the decay of *M*‐*E*‐stable into *P*‐*E*‐stable following the coupled motion mechanism could not be directly monitored. Nevertheless, as both species have similar stabilities, the exchange dynamics between these two species (step VI) was investigated by VT‐EXSY, allowing for the determination of a Gibbs free energy of activation ΔG^≠^_exp step VI_=66.1 kJ/mol for the conversion of *M‐E‐*stable to *P‐E‐*stable corresponding to a half‐life time of 67 ms at room temperature.

Based on the detailed analysis of all steps and intermediates in the cycle, molecular motor **M1** was shown to perform unidirectional 360° rotation following a 6‐step rotation cycle resulting in the coupled dynamic helicity inversion of the stator half (steps I/II and IV/V).

## Conclusion

In conclusion, a new second‐generation molecular motor featuring two intermeshed helical moieties was synthesized. This motor design was specifically engineered with the aim of producing a coupled helicene inversion motion upon actuation of the motor via a central to helical to helical chiral transmission mechanism. This complex interplay of two point stereogenic centers, an overcrowded alkene fragment and two helices was characterized using various spectroscopic techniques such as VT NMR, UV/Vis and CD spectroscopy. This sophisticated system indeed allows to make use of the controlled unidirectional rotary motion around the central overcrowded‐alkene double bond to perform the coupled inversion of a helicene fragment. Hence, we have demonstrated the mechanical transmission of motion across distance allowing for the modulation of a labile stereochemical element i.e. helicity. Such coupled helix inversion motion is of major interest for the design of future more sophisticated molecular machines and functional systems capable of performing more complex tasks. Specifically, stereogenically stable helicenes have been a building block of high importance in materials, for example as chiral dopants in polymers and liquid crystals, or for the control of Circularly Polarized Luminescence (CPL) with applications ranging from organic electronics to cryptography.^[61]^ In this study, we demonstrated the ability of a specifically designed light‐driven molecular motor to remotely invert the handedness of a helicene moiety upon actuation.

## Supporting Information

The authors have cited additional references within the Supporting Information.[^70^, ^71^, ^72^]

## Conflict of Interests

The authors declare no conflict of interest.

1

## Supporting information

As a service to our authors and readers, this journal provides supporting information supplied by the authors. Such materials are peer reviewed and may be re‐organized for online delivery, but are not copy‐edited or typeset. Technical support issues arising from supporting information (other than missing files) should be addressed to the authors.

Supporting Information

Supporting Information

## Data Availability

The data that support the findings of this study are available in the supplementary material of this article.
