# Supplementary material for: Coupling Rotary Motion to Helicene Inversion within a Molecular Motor
Source: Angew Chem Int Ed Engl. 2024 Nov 26;64(4):e202416097. doi: 10.1002/anie.202416097 (PMC11753609; doi:10.1002/anie.202416097)
Supplement: Supplementary file 2 — Supporting Information [file ANIE-64-e202416097-s002.pdf]

Angewandte Chemie
Eine Zeitschrift der Gesellschaft Deutscher Chemiker

Supporting Information

Coupling Rotary Motion to Helicene Inversion within a Molecular Motor

Y. Gisbert, M. Ovalle, C. N. Stindt, R. Costil, B. L. Feringa**

Supporting Information

Coupling Rotary Motion to Helicene Inversion within a Molecular Motor

Yohan Gisbert,* Marco Ovalle, Charlotte N. Stindt, Romain Costil, Ben L. Feringa*

Stratingh Institute for Chemistry, University of Groningen, Nijenborgh 3, 9747 AG Groningen (The Netherlands)

Table of contents

1. Supplementary experimental procedures ___ 2

1.1. General methods ___ 2
1.1. Computational methods ___ 3
1.2. Synthetic procedures ___ 3

2. In-situ NMR irradiation and relaxation experiments ___ 11
3. Exchange spectroscopy (VT-EXSY) ___ 20
4. Variable-temperature UV-vis spectroscopy ___ 26
5. Variable-temperature circular dichroism ___ 30
6. Computational analysis ___ 34
7. HPLC chromatograms ___ 36
8. NMR spectra of new compounds ___ 40
9. References ___ 69

1. Supplementary experimental procedures

1.1. General methods

Commercial reagents and solvents: All chemicals and solvents were purchased from commercial suppliers unless otherwise stated. Anhydrous solvents were obtained using a MBraun SPS 800 system and stored under N_2.

Synthesized reagents: 8-Fluoro-1-tetralone,[1] 2-Methylene-1-oxo-1,2,3,4-tetrahydronaphthalene,[2] (2*S*,3*S*)-2,3-dimethyl-2,3-dihydro-1*H*-cyclopenta[a]naphthalen-1-one[3] and (2*R*,3*R*)-2,3-dimethyl-2,3-dihydro-1*H*-cyclopenta[a]naphthalen-1-one[4] were synthesized according to reported literature procedures and characterized using routine characterization techniques.

Synthesis and purification: Standard Schlenk techniques were used, employing nitrogen or argon as the inert gas. If they were not performed at room temperature, the reaction temperatures refer to the temperature of the heating/cooling bath or heating block.

Flash column chromatography was performed on a Biotage Selekt system using the indicated solvents. TLC analysis was done on Merck silica gel 60 F_{254} aluminum sheets, and compounds were visualized with a UV lamp (254 nm or 365 nm).

Analysis:

NMR: Full characterization of the newly synthesized compounds (including ^{1}H, ^{13}C, ^{19}F, ^{29}Si and 2D NMR experiments) was performed using a Bruker Avance Neo 600 (600 MHz) spectrometer. Chemical shifts (δ) are given in parts per million (ppm) relative to TMS, using the solvent residual peak as internal standard ($CDCl_3$: δ = 7.26 for ^{1}H, δ = 77.16 for ^{13}C; CD_2Cl_2: δ = 5.32 for ^{1}H, δ = 53.84 for ^{13}C). Chemical shifts of P-*E*-stable and M-*Z*-stable isomers were used as internal references (-97.64 ppm and -97.23, respectively) for the kinetic experiments. Data is reported as follows: chemical shifts (δ) in ppm, multiplicity (s = singlet, d = doublet, dd = doublet of doublets, ddd = doublet of doublets of doublets, td = triplet of doublets, t = triplet, q = quartet, br. = broad, m = multiplet), coupling constants *J* (Hz), and integration. Signals were assigned with the help of 2D NMR experiments. Variable temperature NMR and in-situ irradiation experiments were performed using a Varian Inova 500 (500 MHz) spectrometer. NMR irradiation experiments were performed at the indicated temperature with a fiber-coupled LED and a 1500 µm optical fiber (FT1500UMT) to guide the light directly into the NMR tube inside the NMR spectrometer.

The experimental data was fitted by the default Levenberg-Marquardt COPASI[5] algorithm with a tolerance of $1x10^{-6}$ h^{-1}. The initial guess for the kinetic parameter estimation was a random value.

High-resolution mass (HMRS) spectra were recorded on a Thermofisher LTQ Orbitrap XL.

HPLC analysis was performed using a Shimadzu SPD M10AVP diode array detector using Chiralcel columns with mixtures of HPLC-grade *n*-heptane and 2-propanol as the eluent and a column temperature of 40 to 80 °C.

UV/Vis absorption spectra were recorded on a Agilent Cary 8454 spectrophotometer in 1 cm quartz cuvettes.

CD spectra were recorded on a Jasco J-715 spectropolarimeter. The LEDs were attached via a 1500 µm optical fiber (M93L01).

Low temperature spectroscopic experiments were performed using an Unisoku Cryostat (CoolSpek) which was coupled to the spectrophotometer or spectropolarimeter.

Irradiation experiments were performed using fiber-coupled LEDs (M365F1) powered with a T-Cube™ LEDD1B driver obtained from Thorlabs Inc.

1.1. Computational methods

All calculations were performed using the Orca 5.0.4 package.[6] Geometries were optimized with the composite method r^{2}SCAN-3c,[7] using the conductor-like polarizable continuum CPCM(Toluene) solvent model.[8] The thermochemical data were calculated at the same level of theory. The minima and transition states had no or one imaginary frequency, respectively.

1.2. Synthetic procedures

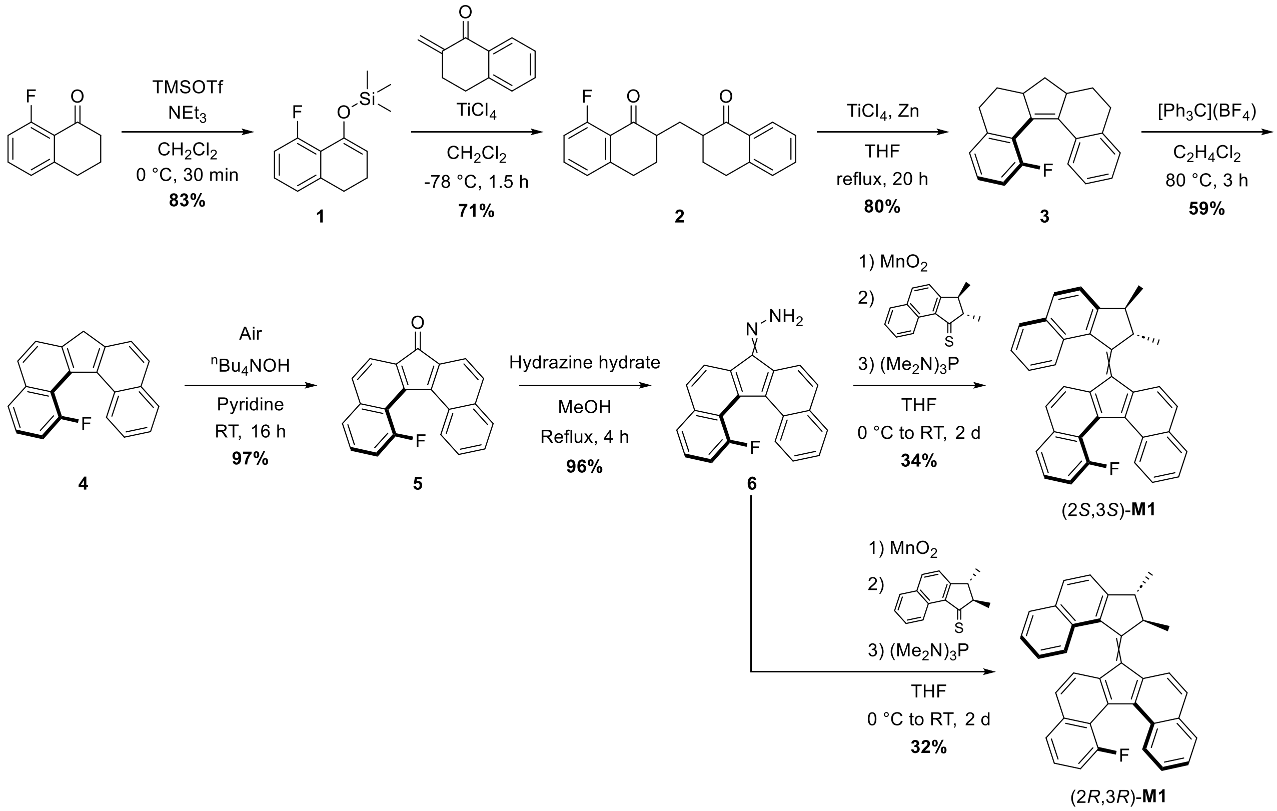

(8-fluoro-3,4-Dihydro-1-naphthyloxy)trimethylsilane (1)

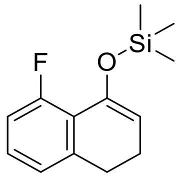

8-Fluoro-1-tetralone[1] (1.75 g, 10.66 mmol, 1 equiv.) was dissolved in anhydrous dichloromethane (16 mL) and cooled down to 0 °C under a nitrogen atmosphere. Then, triethylamine (1.6 mL, 11.72 mmol, 1.1 equiv.) and trimethylsilyl trifluoromethanesulfonate (1.9 mL, 10.66 mmol, 1.0 equiv.) were successively added. The resulting solution was stirred at this temperature for 30 min before being filtered over a short silica plug eluted with a pentane. Solvents were removed to afford (8-fluoro-3,4-dihydro-1-naphthyloxy)trimethylsilane **1** as a transparent oil in 83% yield (2.09 g, 8.84 mmol). The obtained silyl enol ether was either used immediately or temporarily stored in a freezer at –20 °C and used within a few days.

***R*f** = 0.26 (SiO_2, pentane). **^{1}H NMR** (600 MHz, $CDCl_3$, 25 °C): δ = 7.11 – 7.05 (m, 1H), 6.90 (d, *J* = 7.4 Hz, 1H), 6.87 (dd, *J* = 11.7, 8.3 Hz, 1H), 5.28 (t, *J* = 4.9 Hz, 1H), 2.72 (t, *J* = 7.9 Hz, 2H), 2.29 – 2.20 (m, 2H), 0.24 (s, 9H) ppm. **^{13}C{^{1}H} NMR** (151 MHz, $CDCl_3$, 25 °C): δ = 158.9 (d, *J* = 253.3 Hz), 146.8 (d, *J* = 1.8 Hz), 140.8 (d, *J* = 2.6 Hz), 128.3 (d, *J* = 9.1 Hz), 122.9 (d, *J* = 3.2 Hz), 121.3 (d, *J* = 7.1 Hz), 115.2 (d, *J* = 23.4 Hz), 108.5 (d, *J* = 1.7 Hz), 29.1 (d, *J* = 2.7 Hz), 22.0, 0.2 ppm. **^{19}F NMR** (565 MHz, $CDCl_3$, 25 °C): δ = –116.03 – –116.10 (m) ppm. **^{29}Si{^{1}H} NMR** (119 MHz, $CDCl_3$, 25 °C) : δ = 18.92 ppm. **HR-MS** (APCI+): calcd. for $C_{13}H_{18}FOSi$ $[MH]^+$: 237.11055, found 237.11021.

1,5-diketone (2)

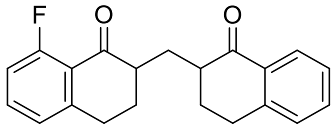

Degassed anhydrous dichloromethane (35 mL) was cooled down to –78 °C under an inert atmosphere. $TiCl_4$ (1.0 mL, 9.29 mmol, 1.0 equiv.) was slowly added. Then, a solution of 2-methylene-1-oxo-1,2,3,4-tetrahydronaphthalene (2.1 g, 13.27 mmol, 1.5 equiv.) dissolved in 12 mL of anhydrous dichloromethane and a solution of (8-fluoro-3,4-dihydro-1-naphthyloxy)trimethylsilane **1** (2.09 g, 8.84 mmol, 1 equiv.) dissolved in 12 mL of dichloromethane were successively added. The reaction was stirred at –78 °C for 1.5 h before being warmed-up to room temperature and the mixture quenched by addition of a saturated aqueous solution of potassium carbonate (20 mL). The layers were separated, and the aqueous fraction was washed with dichloromethane (2x20 mL). The combined organic layers were washed with brine (100 mL), dried over magnesium sulfate and the solvents were evaporated in vacuo. The crude product was then adsorbed on celite and purified by flash column chromatography (SiO_2, EtOAc/pentane 0:100 to 15:85) to afford 1,5-diketone **2** as a yellow-orange oil which solidified upon standing in 71% yield (2.03 g, 6.28 mmol). The obtained product is composed of a mixture of all four possible diastereomers.

R_f = 0.30 (SiO_2, acetone/pentane 4:96). **^{1}H NMR** (600 MHz, $CDCl_3$, 25 °C): δ = 7.99 (ddd, J = 7.9, 3.2, 1.4 Hz, 1H), 7.45 (td, J = 7.4, 1.3 Hz, 1H), 7.39 (td, J = 8.0, 5.1 Hz, 1H), 7.29 (td, J = 7.4, 1.2 Hz, 1H), 7.23 (d, J = 7.9 Hz, 1H), 7.02 (dd, J = 7.7, 1.1 Hz, 1H), 6.95 (ddd, J = 11.1, 8.0, 2.3 Hz, 1H), 3.12 – 3.00 (m, 4H), 2.93 – 2.87 (m, 0.38H), 2.86 – 2.81 (m, 0.38H), 2.81 – 2.74 (m, 1H), 2.72 – 2.65 (m, 0.62H), 2.41 – 2.24 (m, 2H), 1.98 (t, J = 6.6 Hz, 1H), 1.96 – 1.86 (m, 1.62H), 1.65 – 1.54 (m, 1H) ppm. **^{13}C{^{1}H} NMR** (151 MHz, $CDCl_3$, 25 °C): δ = 201.2, 200.4, 199.2, 198.6, 162.4 (d, J = 264.7 Hz), 162.3 (d, J = 264.4 Hz), 146.3, 146.3, 144.1, 144.0, 134.2 (d, J = 10.4 Hz), 134.2 (d, J = 10.3 Hz), 133.4, 133.4, 132.7, 132.5, 128.9, 128.9, 127.5, 127.4, 126.7, 126.7, 124.5, 124.5, 121.8 (d, J = 5.2 Hz), 121.6 (d, J = 5.0 Hz), 115.0 (d, J = 22.2 Hz), 114.9 (d, J = 22.1 Hz), 47.3, 46.1, 46.0, 45.0, 31.4, 30.1, 29.8, 29.5, 29.2, 29.1 (d, J = 2.3 Hz), 29.0, 28.8, 28.8 (d, J = 2.5 Hz), 28.6 ppm. **^{19}F NMR** (565 MHz, $CDCl_3$, 25 °C): δ = –112.48 (dd, J = 11.2, 5.2 Hz, 0.62F), –112.77 (dd, J = 11.2, 5.2 Hz, 0.38F) ppm. **HR-MS** (ESI+): calcd. for $C_{21}H_{19}FO_2Na$ $[M+Na]^+$: 345.12613, found 345.12394.

Hexahydro-dibenzofluorene (3)

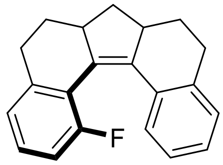

Zinc powder (4.06 g, 62.04 mmol, 10 equiv.) was stirred vigorously under an inert atmosphere for one hour in order to partially break the oxidation layer. Degassed anhydrous tetrahydrofuran (60 mL) was then added and the suspension was cooled to 0 °C before slowly adding $TiCl_4$ (2.7 mL, 24.82 mmol, 4 equiv.). The mixture was then heated at reflux for 3 h before being allowed to cool down to room temperature. A solution of diketone **2** (2.00 g, 6.20 mmol, 1 equiv.) dissolved in tetrahydrofuran (10 mL) was then added dropwise over 2 h using a syringe pump. The reaction mixture was then heated at reflux for 16 h. After cooling down to room temperature, the reaction mixture was filtered over celite and washed with 75 mL of a 1M aqueous HCl solution, followed by water (75 mL) and brine (75 mL). The organic layer was then dried over magnesium sulfate and the solvents were removed in vacuo. The crude product was then purified by column chromatography (SiO_2, CH_2Cl_2/pentane 0:100 to 5:95) to afford the hydrogenated dibenzofluorene analogue **3** as a white solid in 80% yield (1.44 g, 4.97 mmol). The obtained product is composed of a mixture of all four possible diastereomers.

R_f = 0.52 (SiO_2, pentane). **^{1}H NMR** (600 MHz, $CDCl_3$, 25 °C): δ = 7.50 (t, J = 6.3 Hz, 0.3H), 7.30 (dd, J = 7.4, 5.6 Hz, 0.6H), 7.20 – 7.16 (m, 0.4H), 7.13 – 7.05 (m, 2.5H), 7.01 – 6.95 (m, 1.6H), 6.91 (dd, J = 7.6, 1.1 Hz, 0.7H), 6.84 – 6.80 (m, 0.7H), 3.13 – 2.97 (m, 4.2H), 2.93 (ddq, J = 14.0, 6.9, 2.3 Hz, 0.7H), 2.88 (t, J = 3.3 Hz, 0.2H), 2.85 (d, J = 3.3 Hz, 0.2H), 2.75 (dtd, J = 14.1, 4.0, 1.9 Hz, 0.3H), 2.59 – 2.52 (m, 0.4H), 2.46 (dt, J = 11.1, 6.0 Hz, 0.4H), 2.26 – 2.15 (m, 1.7H), 2.02 (ddd, J = 13.3, 8.8, 2.4 Hz, 0.7H), 1.97 – 1.79 (m, 2.6H), 1.48 (dtd, J = 13.3, 12.4, 3.7 Hz, 0.3H), 1.38 (q, J = 11.2 Hz, 0.4H) ppm. **^{13}C{^{1}H} NMR** (151 MHz, $CDCl_3$, 25 °C): δ = 158.7 (d, J = 249.6 Hz), 158.6 (d, J = 250.0 Hz), 144.4 (d, J = 4.1 Hz), 140.9 (d, J = 4.6 Hz), 139.2, 137.8, 137.6, 135.1, 134.0 (d, J = 2.6 Hz), 132.5, 128.9, 128.9, 128.7, 128.2 (d, J = 8.7 Hz), 128.2, 127.7 (d, J = 8.6 Hz), 127.3, 127.1, 126.6 (d, J = 5.9 Hz), 126.2 (d, J = 5.4 Hz), 125.7, 124.8, 124.7, 124.3 (d, J = 3.1 Hz), 124.2 (d, J = 17.3 Hz), 122.8 (d, J = 2.8 Hz), 113.4 (d, J = 22.2 Hz), 112.8 (d, J = 22.1 Hz), 47.0, 46.9, 46.2, 43.4, 40.3, 34.2, 32.1, 32.1, 31.6, 30.5, 29.5, 29.5 (d, J = 2.4 Hz), 29.2, 28.8 (d, J = 2.6 Hz) ppm. **^{19}F NMR** (565 MHz, $CDCl_3$, 25 °C): δ = –106.43 (dt, J = 10.2,

5.3 Hz, 0.67F), –108.13 (dt, J = 10.2, 6.0 Hz, 0.33F) ppm. **HR-MS** (ESI+): calcd. for $C_{21}H_{20}F$ $[M+H]^+$: 291.15436, found 291.15415.
Due to the presence of multiple isomers in the obtained pure samples, signals in the ^1H NMR spectrum were integrated with respect to the total number of protons in the compounds (19 H). For ^{13}C NMR, all signals were listed without taking the multiplicity induced by the coupling with fluorine into account, as the obtained spectrum was too complex to be assigned using 2D NMR methods as for the previous fluorinated precursors.

1-Fluoro-dibenzo[*c*,*g*]fluorene (4)

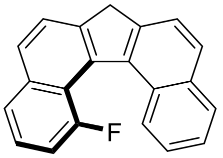

Hydrogenated dibenzofluorene analogue **4** (1.44 g, 4.96 mmol, 1 equiv.) was dissolved in anhydrous dichloroethane (125 mL). Tritylium tetrafluoroborate (9.82 g, 29.75 mmol, 6 equiv.) was then added under an inert atmosphere and the mixture was stirred at 80 °C for 3 h. The mixture was then cooled down to room temperature, filtered through neutral alumina (CH_2Cl_2) and the solvents were removed by rotary evaporation. The crude product was then purified by column chromatography (SiO_2, CH_2Cl_2/Pentane 0:100 to 5:95) to afford 1-fluoro-dibenzo[*c*,*g*]fluorene **4** as a white solid in a 59% yield (0.83 g, 2.93 mmol).

R_f = 0.38 (SiO_2, pentane). **^{1}H NMR** (600 MHz, $CDCl_3$, 25 °C): δ = 8.32 – 8.25 (m, 1H), 7.99 – 7.93 (m, 1H), 7.88 (d, J = 8.1 Hz, 1H), 7.84 (dd, J = 8.1, 1.8 Hz, 1H), 7.79 (d, J = 8.1 Hz, 1H), 7.73 (dd, J = 8.1, 2.6 Hz, 2H), 7.55 – 7.45 (m, 3H), 7.29 (ddd, J = 11.6, 7.6, 1.1 Hz, 1H), 4.09 (br. s, 2H) ppm. **^{13}C{^{1}H} NMR** (151 MHz, $CDCl_3$, 25 °C): δ = 158.6 (d, J = 252.7 Hz), 143.8, 142.2, 138.9, 135.9 (d, J = 4.8 Hz), 134.4, 133.6, 130.0 (d, J = 3.7 Hz), 128.0, 127.9, 126.8 (d, J = 13.9 Hz), 126.6 (d, J = 2.9 Hz), 125.1, 125.0, 124.3 (d, J = 3.3 Hz), 124.3 (d, J = 2.2 Hz), 123.6, 122.6, 120.2 (d, J = 16.2 Hz), 111.1 (d, J = 21.7 Hz), 38.8 ppm. **^{19}F NMR** (565 MHz, $CDCl_3$, 25 °C): –98.09 (td, J = 12.0, 5.1 Hz). **HR-MS** (APCI+): calcd. for $C_{21}H_{14}F$ $[M+H]^+$: 285.10741, found 285.10686.

1-Fluoro-dibenzo[*c*,*g*]fluorenone (5)

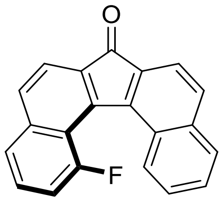

To a solution of 1-fluoro-dibenzo[*c*,*g*]fluorene **4** (50 mg, 0.18 mmol, 1 equiv.) was added pyridine (2 mL) in an oversized 50 mL round bottom flask equipped with the largest possible ovoid stir bar. A 1M solution of tetrabutylammonium hydroxide in methanol (50 µL, 0.05 mmol, 0.28 equiv.) was added, inducing a sudden color change to dark purple. The mixture was submitted to strong stirring at room temperature without closing the flask for four hours. The pyridine was then evaporated and the residue was redissolved in dichloromethane (10 mL) and washed with a 0.1 M aqueous HCl solution (2x10 mL) followed by brine (10 mL). The organic layer was then dried over magnesium sulfate and the solvent was removed by rotary evaporation. The crude product was adsorbed onto celite and purified by column chromatography (SiO_2, CH_2Cl_2/pentane 2:8 to 4:6) to afford pure 1-fluoro-dibenzo[*c*,*g*]fluorenone **5** as a purple solid in 97% yield (51 mg, 0.17 mmol).

***R*f** = 0.29 (SiO_2, CH_2Cl_2/pentane 30:70). **^{1}H NMR** (600 MHz, $CDCl_3$, 25 °C): δ = 8.00 (dd, *J* = 11.9, 8.6 Hz, 1H), 7.84 – 7.76 (m, 3H), 7.75 (d, *J* = 7.9 Hz, 1H), 7.72 (d, *J* = 8.0 Hz, 1H), 7.67 (d, *J* = 8.3 Hz, 1H), 7.54 – 7.45 (m, 2H), 7.43 (t, *J* = 7.7 Hz, 1H), 7.20 (dd, *J* = 11.4, 7.5 Hz, 1H) ppm. **^{13}C{^{1}H} NMR** (151 MHz, $CDCl_3$, 25 °C): δ = 193.7, 158.4 (d, *J* = 255.4 Hz), 147.5, 143.4, 140.2 (d, *J* = 3.8 Hz), 138.8, 134.7, 132.0, 130.3, 129.4 (d, *J* = 2.8 Hz), 129.3 (d, *J* = 3.3 Hz), 128.7, 128.3, 128.2, 127.4 (d, *J* = 14.0 Hz), 125.9 (d, *J* = 2.1 Hz), 125.2 (d, *J* = 3.8 Hz), 120.5 (d, *J* = 16.6 Hz), 120.4, 119.8, 112.4 (d, *J* = 21.2 Hz) ppm. **^{19}F NMR** (565 MHz, $CDCl_3$, 25 °C): δ = –97.43 (td, *J* = 11.8, 5.0 Hz) ppm. **HR-MS** (ESI+): calcd. for $C_{21}H_{12}FO$ $[M+H]^+$: 299.08667, found 299.08641.

(1-Fluoro-dibenzo[*c*,*g*]fluoren-7-ylidene)hydrazine (6)

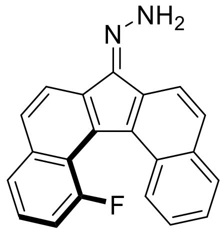

1-Fluoro-dibenzo[*c*,*g*]fluorenone **5** (258 mg, 0.86 mmol, 1 equiv.) was dissolved in methanol (7 mL) and the mixture was degassed and heated to 64 °C under an inert atmosphere. Hydrazine hydrate (1.4 mL, 22.92 mmol, 26.5 equiv.) was added and the solution was stirred at 64 °C for 3 h. After cooling down to room temperature, water (50 mL) was added and the mixture was extracted with ethyl acetate (3x50 mL). The combined organic layers were washed with brine (100 mL) and dried over magnesium sulfate. The solvents were then removed, the crude product was adsorbed on celite and purified by flash column chromatography (SiO_2, CH_2Cl_2/pentane 50:50 to 100:0) to afford hydrazone **6** as an orange solid composed of a mixture of *Z* and *E* isomers in 96% yield (260 mg, 0.83 mmol).

***R*f** = 0.28 (SiO_2, CH_2Cl_2/pentane 50:50). **^{1}H NMR** (600 MHz, CD_2Cl_2, 25 °C): 8.18 (ddd, *J* = 12.2, 8.5, 1.0 Hz, 0.85H), 8.12 (d, *J* = 8.3 Hz, 0.15H), 8.06 (d, *J* = 8.4 Hz, 1H), 7.96 (d, *J* = 8.2 Hz, 0.85H), 7.93 – 7.87 (m, 2.15H), 7.86 – 7.82 (m, 1H), 7.79 – 7.75 (m, 1H), 7.53 (ddd, *J* = 8.1, 6.7, 1.2 Hz, 1H), 7.48 – 7.41 (m, 2H), 7.29 – 7.25 (m, 0.15H), 7.23 (ddd, J = 11.8, 7.6, 1.1 Hz, 0.85H), 6.93 (br. s, 1.7H), 6.90 (br. s, 0.3H) ppm. **^{13}C{^{1}H} NMR** (151 MHz, CD_2Cl_2, 25 °C): δ = 159.1 (d, *J* = 253.9 Hz), 158.7 (d, *J* = 252.3 Hz), 144.0, 143.8, 140.7, 139.0, 137.2 (d, *J* = 4.7 Hz), 136.9 (d, *J* = 4.3 Hz), 136.7, 136.6, 136.2, 135.0, 134.9, 132.0, 129.8 (d, *J* = 4.0 Hz), 129.7 (d, *J* = 3.8 Hz), 129.2, 129.1, 128.8, 128.6, 128.1 (d, *J* = 1.1 Hz), 128.0 (d, *J* = 2.7 Hz), 127.8, 127.7, 127.7, 127.3, 126.9 (d, *J* = 8.5 Hz), 126.9, 126.77, 125.7 (d, *J* = 8.5 Hz), 125.6, 125.3 (d, *J* = 2.2 Hz), 125.1 (d, *J* = 2.2 Hz), 125.0 (d, *J* = 3.5 Hz), 124.6 (d, *J* = 3.5 Hz), 122.7, 121.8, 120.2 (d, *J* = 15.9 Hz), 119.6, 118.6, 112.1 (d, *J* = 21.3 Hz), 111.9 (d, *J* = 21.4 Hz) ppm. **^{19}F NMR** (565 MHz, CD_2Cl_2, 25 °C): –97.99 (td, *J* = 11.9, 5.1 Hz, 0.15F), –99.13 (td, *J* = 12.0, 5.1 Hz, 0.85F) ppm. **HR-MS** (ESI+): calcd. for $C_{21}H_{14}FN_2$ $[M+H]^+$: 313.11355, found 313.11390.

(2*S*,3*S*)-**M1**

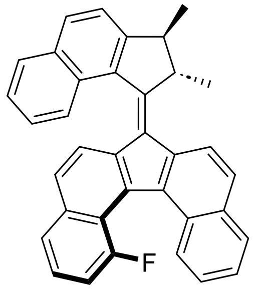

(2*S*,3*S*)-2,3-Dimethyl-2,3-dihydro-1*H*-cyclopenta[a]naphthalen-1-one (200 mg, 0.95 mmol, 1 equiv.) and Lawesson's Reagent (1.15 g, 2.85 mmol, 3 equiv.) were suspended in degassed anhydrous toluene (10 mL). The mixture was stirred at 100 °C for 4 h. After cooling down to room temperature, the mixture was directly transferred to a short chromatography column (SiO_2, CH_2Cl_2/Pentane 0:100 to 10:90). The red fraction (red on SiO_2, purple to blue in solution) was collected. Solvents were removed, affording (2*S*,3*S*)-2,3-dimethyl-2,3-dihydro-1*H*-cyclopenta[a]naphthalen-1-thione in 84% yield (181 mg, 0.80 mmol) which was immediately used without any further purification for the next step.

(1-Fluoro-dibenzo[*c*,*g*]fluoren-7-ylidene)hydrazine **6** (175 mg, 0.56 mmol, 1.0 equiv.) was dissolved in anhydrous tetrahydrofuran (15 mL). Anhydrous sodium sulfate (358 mg, 2.52 mmol, 4.5 equiv.) was added and the suspension was degassed by bubbling argon for 10 min. The reaction mixture was cooled down to 0 °C and placed in the dark. MnO_2 (146 mg, 1.68 mmol, 3 equiv.) was added and the suspension was stirred at 0 °C for 15 min. The *in-situ* generated diazo compound was then filtered and transferred to another Schlenk flask containing (2*S*,3*S*)-2,3-dimethyl-2,3-dihydro-1*H*-cyclopenta[a]naphthalen-1-thione (181 mg, 0.80 mmol, 1.4 equiv.) by cannula filtration. An extra 5 mL of THF was used for rinsing. The resulting solution was then stirred at room temperature for 24 h in the dark before tris(dimethylamino)phosphine (0.3 mL, 1.68 mmol, 3 equiv.) was added. The reaction mixture was stirred for a further 16 h and the volatiles were removed in vacuo. The crude product was adsorbed on silica and purified by column chromatography (SiO_2, CH_2Cl_2/pentane, 0:100 to 10:90) to yield pure (2*S*,3*S*)-**M1** as an orange solid in 34% yield (91 mg, 0.19 mmol). The obtained product is a mixture of the *Z* and *E* isomers obtained in a varying ratio depending on the handling conditions.

***R*$_f$** = 0.53 (SiO_2, CH_2Cl_2/pentane 10:90). **^{1}H NMR** (600 MHz, CD_2Cl_2, 25 °C): 8.26 (d, *J* = 8.6 Hz, 0.49H), 8.24 – 8.13 (m, 1.50H), 8.11 (d, *J* = 8.4 Hz, 0.47H), 8.08 – 7.88 (m, 3.72H), 7.82 (d, *J* = 8.1 Hz, 0.57H), 7.76 – 7.69 (m, 0.75H), 7.67 – 7.63 (m, 0.90H), 7.61 (d, *J* = 8.3 Hz, 0.61H), 7.52 – 7.22 (m, 6.74H), 7.19 – 7.13 (m, 0.46H), 7.10 (d, *J* = 8.9 Hz, 0.23H), 7.02 (d, *J* = 8.7 Hz, 0.48H), 7.01 – 6.98 (m, 0.34H), 4.14 – 4.10 (m, 0.15H), 4.07 (q, *J* = 6.8 Hz, 0.49H), 4.03 – 3.94 (m, 0.24H), 3.26 – 3.18 (m, 0.90H), 1.55-1.51 (m, 1.10H), 1.47 – 1.43 (m, 1.97H), 1.41 – 1.36 (m, 2.90H) ppm. **^{13}C{^{1}H} NMR** (151 MHz, CD_2Cl_2, 25 °C): δ = 160.0, 158.3, 155.6, 155.4, 154.7, 154.7, 154.4, 142.1, 139.6, 138.6, 138.4, 138.1, 137.4, 136.5, 136.2, 136.1, 136.0, 136.0, 135.6, 135.5, 135.4, 134.0, 133.9, 133.7, 133.7, 133.5, 133.5, 133.4, 132.5, 132.4, 132.3, 132.3, 132.1, 131.9, 131.0, 130.9, 130.4, 130.1, 129.8, 129.8, 129.8, 129.6, 129.3, 129.2, 129.2, 129.1, 129.1, 128.6, 128.1, 128.0, 127.9, 127.7, 127.6, 127.5, 127.5, 127.4, 127.4, 127.3, 127.1, 127.1, 127.0, 126.8, 126.7, 126.6, 126.5, 126.1, 126.0, 125.9, 125.9, 125.9, 125.7, 125.7, 125.6, 125.5, 125.5, 125.3, 125.3, 124.9, 124.9, 124.9, 124.7, 124.7, 124.6, 124.5, 124.5, 124.4, 124.4, 124.4, 124.3,

124.1, 123.9, 123.8, 123.5, 122.6, 122.5, 122.4, 122.1, 122.1, 121.8, 121.6, 120.7, 120.6, 111.6, 111.4, 111.3, 50.3, 21.9, 19.7 ppm. **^{19}F NMR** (565 MHz, CD_2Cl_2, 25 °C): δ = –97.28 (m, *M*-*E*-stable-**M1**), –97.88 (m, *P*-*Z*-stable-**M1**), –98.69 (*M*-*Z*-stable-**M1**), –99.45 (td, *J* = 11.7, 5.1 Hz, *P*-*E*-stable-**M1**) ppm. **HR-MS** (ESI+): calcd. for $C_{36}H_{26}F$ $[M+H]^+$: 477.20131, found 477.20078. **HPLC** (Daicel CHIRALCEL OD-H, ^{i}PrOH/^{n}heptane 0.5:99.5, 1 mL/min, 80 °C, 395 nm): retention times (min) 12.30 (*E*- or *Z*- (2*R*,3*R*)**-M1**), 13.46 (*E*- or *Z*- (2*R*,3*R*)**-M1**), 16.71 (*E*-(2*S*,3*S*)**-M1**), 21.69 (*Z*-(2*S*,3*S*)**-M1**), *ee* 87%.

Because of the presence of multiple isomers in the obtained pure samples, signals in the ^{1}H NMR spectrum were integrated with respect to the total number of protons in the compounds (25 H). For ^{13}C NMR, all signals were listed without taking the multiplicity induced by the coupling with fluorine into account, as the obtained spectrum was too complex to be assigned using 2D NMR methods as was done for the previous fluorinated precursors. ^{19}F NMR was found to be the most reliable methods to monitor the composition of these isomeric mixtures and signals were assigned accordingly.

Analytical samples of pure or highly enriched *Z* and *E* isomers were obtained by column chromatography (SiO_2, Et_2O/pentane 3:97). In order to obtain enrichment, the column has to be long (approx. 40 cm x 1 cm for 30 mg. of sample) and protected from direct light. Only the head and tail of the product were collected and studied. The first obtained fraction is enriched in *Z* isomer and the last one is enriched in *E* isomer.

A minor isomer not involved in the 8-step rotation cycle was observed by NMR (characteristic signal at -99.3 ppm in ^{19}F NMR) and HPLC. This residual isomer was assigned to be the cis-dimethylated diastereomer, residual from the parent ketone.

(2*R*,3*R*)**-M1**

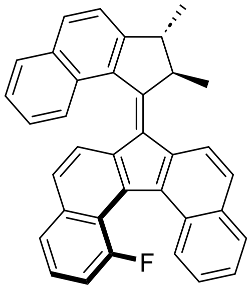

(2*R*,3*R*)-2,3-Dimethyl-2,3-dihydro-1*H*-cyclopenta[a]naphthalen-1-one (110 mg, 0.52 mmol, 1 equiv.) and Lawesson's Reagent (0.64 g, 1.57 mmol, 3 equiv.) were suspended in degassed anhydrous toluene (6 mL). The mixture was stirred at 100 °C for 4 h. After cooling down to room temperature, the mixture was directly transferred to a short chromatography column (SiO_2, CH_2Cl_2/Pentane 0:100 to 10:90). The red fraction (red on SiO_2, purple to blue in solution) was collected. Solvents were removed, affording (2*R*,3*R*)-2,3-dimethyl-2,3-dihydro-1*H*-cyclopenta[a]naphthalen-1-thione in 81% yield (96 mg, 0.42 mmol) which was immediately used without any further purification for the next step.

(1-Fluoro-dibenzo[*c*,*g*]fluoren-7-ylidene)hydrazine **6** (95 mg, 0.30 mmol, 1.0 equiv.) was dissolved in anhydrous tetrahydrofuran (5 mL). Anhydrous sodium sulfate (194 mg, 1.37 mmol,

4.5 equiv.) was added and the suspension was degassed by bubbling argon for 10 min. The reaction mixture was cooled down to 0 °C and placed in the dark. MnO_2 (79 mg, 0.91 mmol, 3 equiv.) was added and the suspension was stirred at 0 °C for 15 min. The *in-situ* generated diazo compound was then filtered and transferred to another Schlenk flask containing (2*R*,3*R*)-2,3-dimethyl-2,3-dihydro-1*H*-cyclopenta[a]naphthalen-1-thione (69 mg, 0.30 mmol, 1 equiv.) by cannula filtration. An extra 1 mL of THF was used for rinsing. The resulting solution was then stirred at room temperature for 24 h in the dark before tris(dimethylamino)phosphine (0.16 mL, 0.91 mmol, 3 equiv.) was added. The reaction mixture was stirred for a further 16 h and the volatiles were removed in vacuo. The crude product was adsorbed on silica and purified by column chromatography (SiO_2, CH_2Cl_2/pentane, 0:100 to 10:90) to yield pure (2*R*,3*R*)**-M1** as an orange solid in 32% yield (46 mg, 0.10 mmol). The obtained product is a mixture of the *Z* and *E* isomers obtained in a varying ratio depending on the handling conditions.

***R*f** = 0.53 (SiO_2, CH_2Cl_2/pentane 10:90). **^{1}H, ^{13}C{^{1}H} and ^{19}F NMR** spectra are identical the ones of (2*S*,3*S*)**-M1**. **HR-MS** (ESI+): calcd. for $C_{36}H_{25}F$ $[M]^+$: 476.19348, found 476.19222. **HPLC** (Daicel CHIRALCEL OD-H, ^{i}PrOH/^{n}heptane 0.5:99.5, 1 mL/min, 80 °C, 395 nm): retention times (min) 12.29 (*E*- or *Z*- (2*R*,3*R*)**-M1**), 13.50 (*E*- or *Z*- (2*R*,3*R*)**-M1**), 16.96 (*E*-(2*S*,3*S*)**-M1**), 22.46 (*Z*-(2*S*,3*S*)**-M1**), *ee* 75%.

2. In-situ NMR irradiation and relaxation experiments

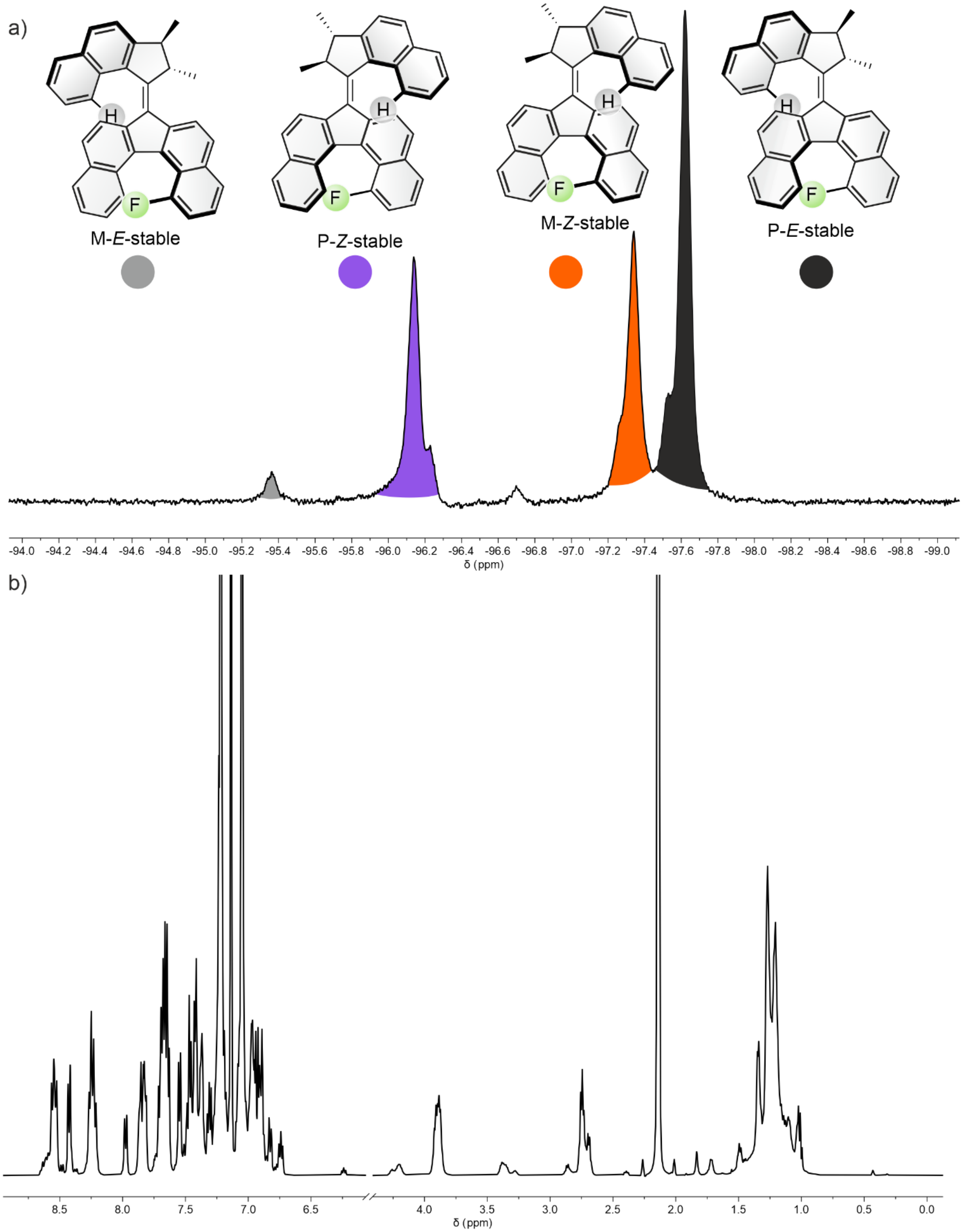

Figure S1. Low temperature NMR (500 MHz, toluene-d_8, –85 °C) spectra of the different isomers obtained from the synthesis of **M1**. a) ^{19}F NMR signals and their assignments highlighted in their respective color. b) ^{1}H NMR spectrum.

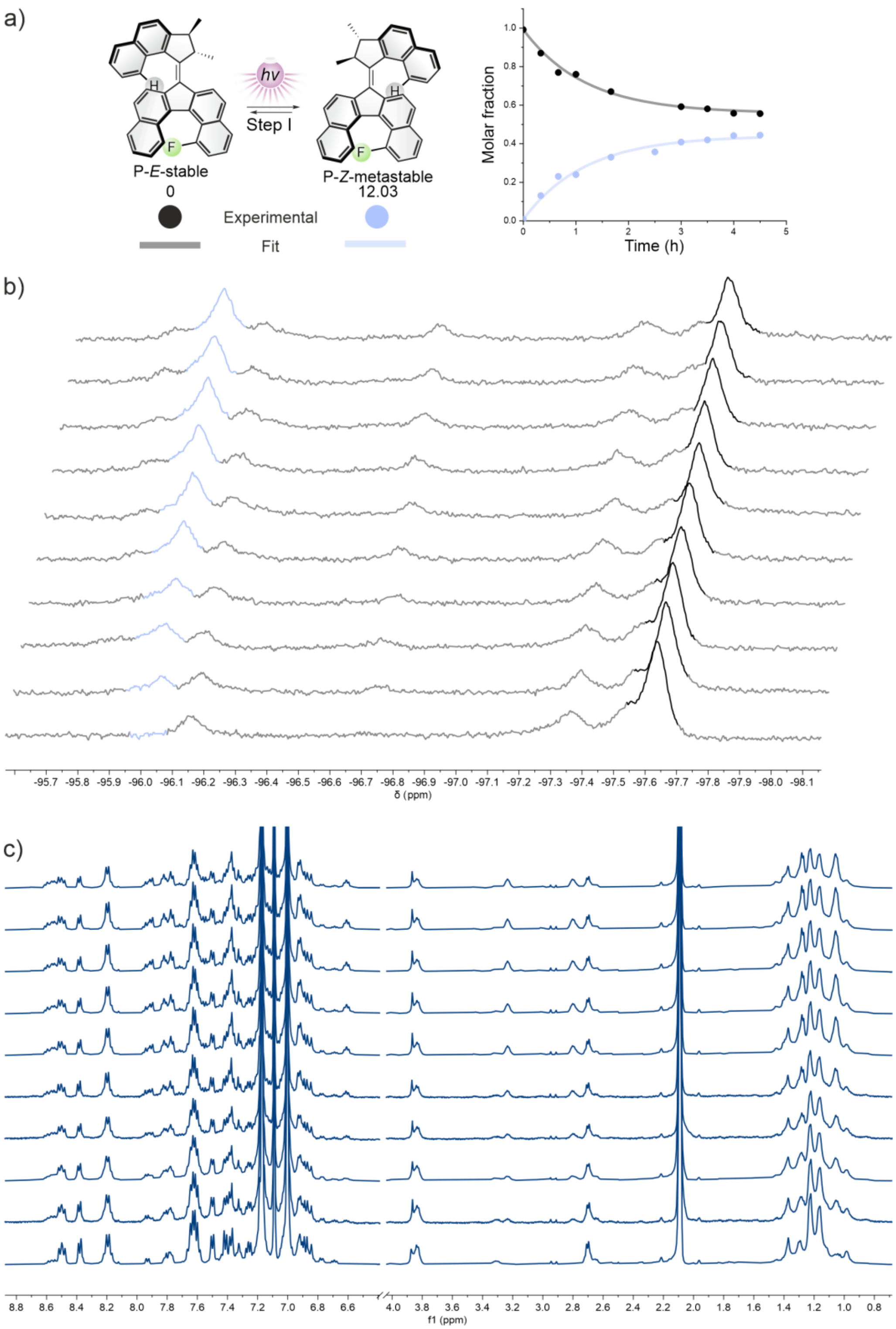

Figure S2. Step I of the rotation cycle. Low temperature NMR (500 MHz, toluene-d$_8$, –85 °C) *in-situ* irradiation (λ_{irr} = 365 nm) of an enriched sample of ***P-E*-stable** (black) is converted to ***P-Z*-metastable** (blue). a) Kinetic traces of the evolution of isomers of **M1** obtained by the integration of ^{19}F NMR signals. b) Evolution of the ^{19}F NMR spectra (from bottom to top). c) Evolution of the ^{1}H NMR spectra (from bottom to top).

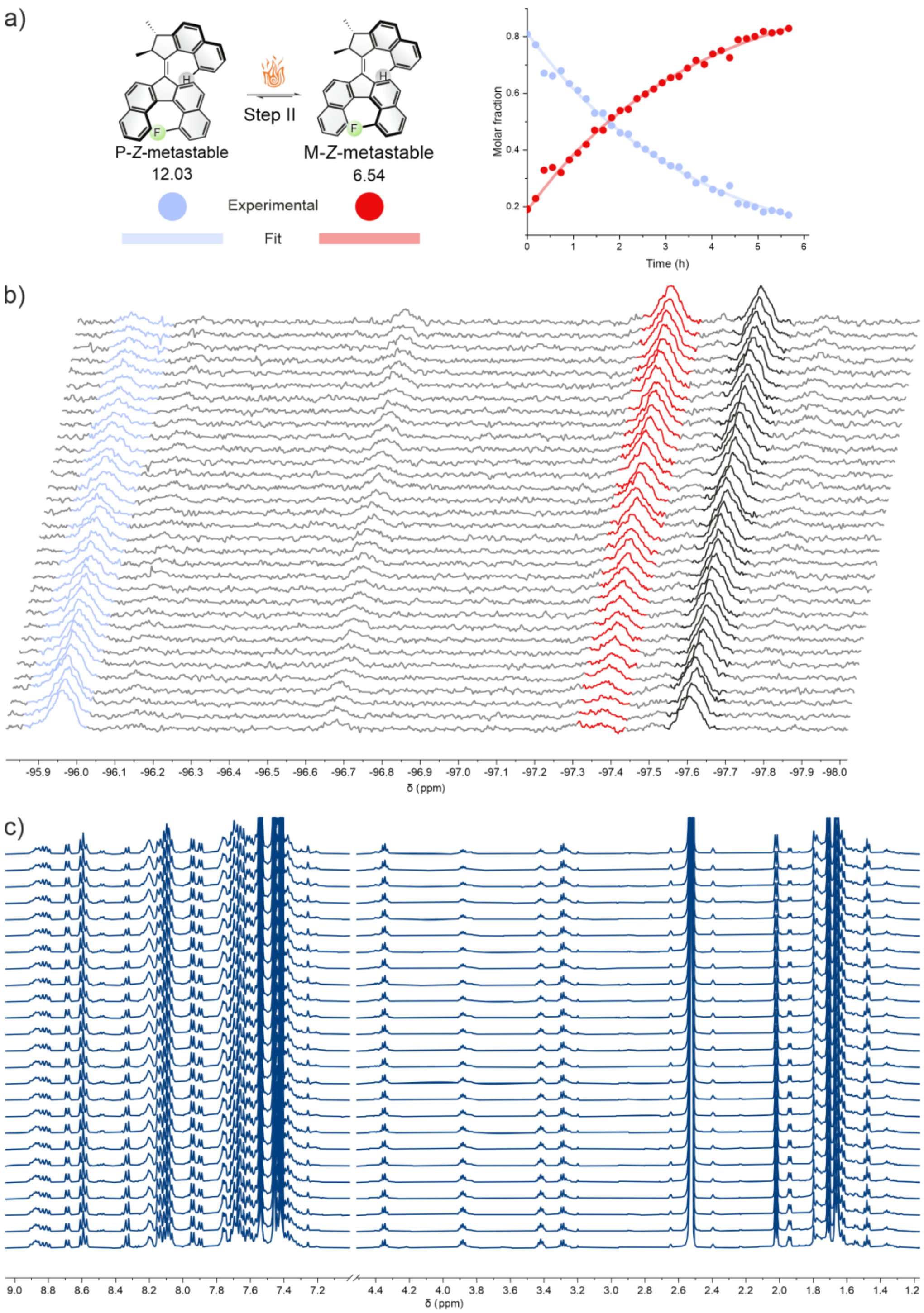

Figure S3. Step II of the rotation cycle. Low temperature NMR (500 MHz, toluene-d_8, –70 °C) *in-situ* thermal helicene inversion of the previously generated ***P-Z*-metastable** (blue) to ***M-Z*-metastable** (red). The (photochemically) unreacted ***P-E*-stable** (black) remains constant during this process. a) Kinetic traces of the evolution of isomers of **M1** obtained by the integration of ^{19}F NMR signals. b) Evolution of the ^{19}F NMR spectra (from bottom to top). c) Evolution of the ^{1}H NMR spectra (from bottom to top). Observed rate constant k = 0.28 h^{-1} @ –70 °C.

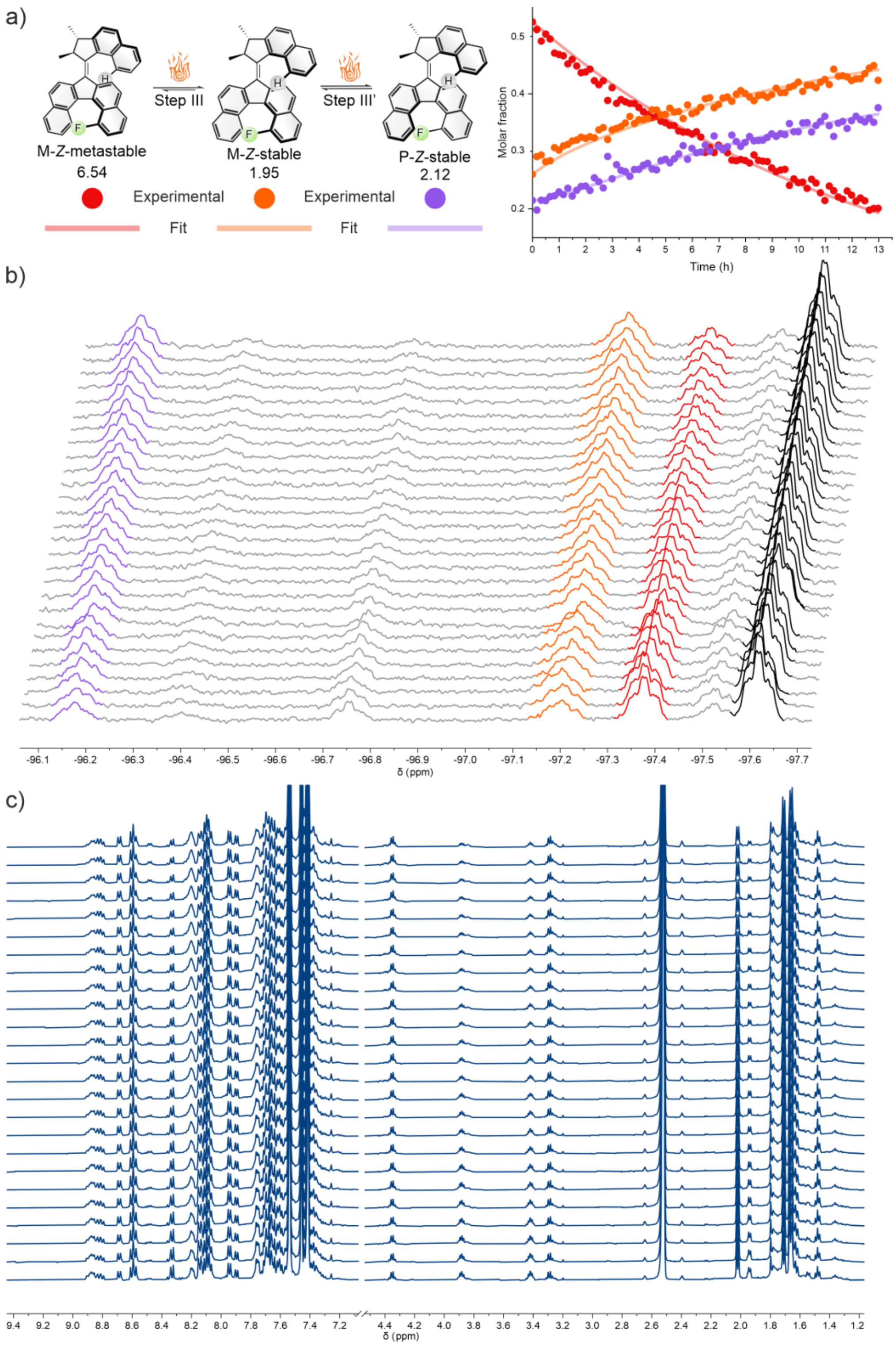

Figure S4. Step III of the rotation cycle. NMR (500 MHz, toluene-d_8, 15 °C) *in*-situ thermal helix and helicene inversion of the previously generated ***M-Z*-metastable** (red) to ***M-Z*-stable** (orange) and ***P-Z*-stable** (purple). The (photochemically) unreacted ***P-E*-stable** (black) remains constant during this process. a) Kinetic traces of the evolution of isomers of **M1** obtained by the integration of ^{19}F NMR signals. b) Evolution of the ^{19}F NMR spectra (from bottom to top). c) Evolution of the ^{1}H NMR spectra (from bottom to top).

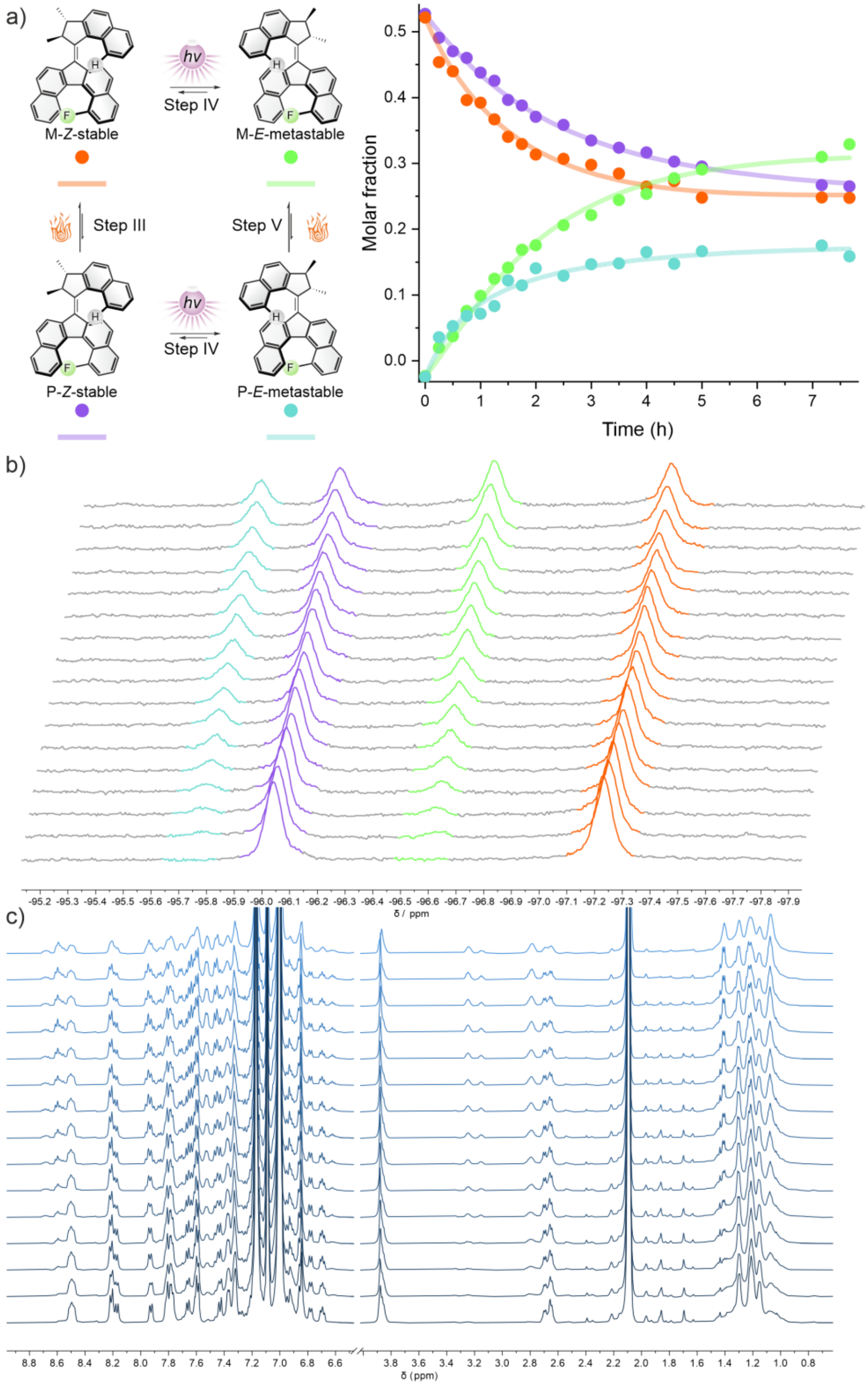

Figure S5. Step IV of the rotation cycle. Low temperature NMR (500 MHz, toluene-d_8, –85 °C) *in-situ* irradiation (λ_{irr} = 365nm) of an enriched sample of ***M-Z*-stable** (orange) and ***P-Z*-stable** (purple) are converted to ***M-E*-metastable** (green) ***P-E*-metastable** (cyan), respectively. At this temperature, the thermal helicene inversion is also observed (step V) a) Kinetic traces of the evolution of isomers of **M1** obtained by the integration of ^{19}F NMR signals. b) Evolution of the ^{19}F NMR spectra (from bottom to top). c) Evolution of the ^{1}H NMR spectra (from bottom to top).

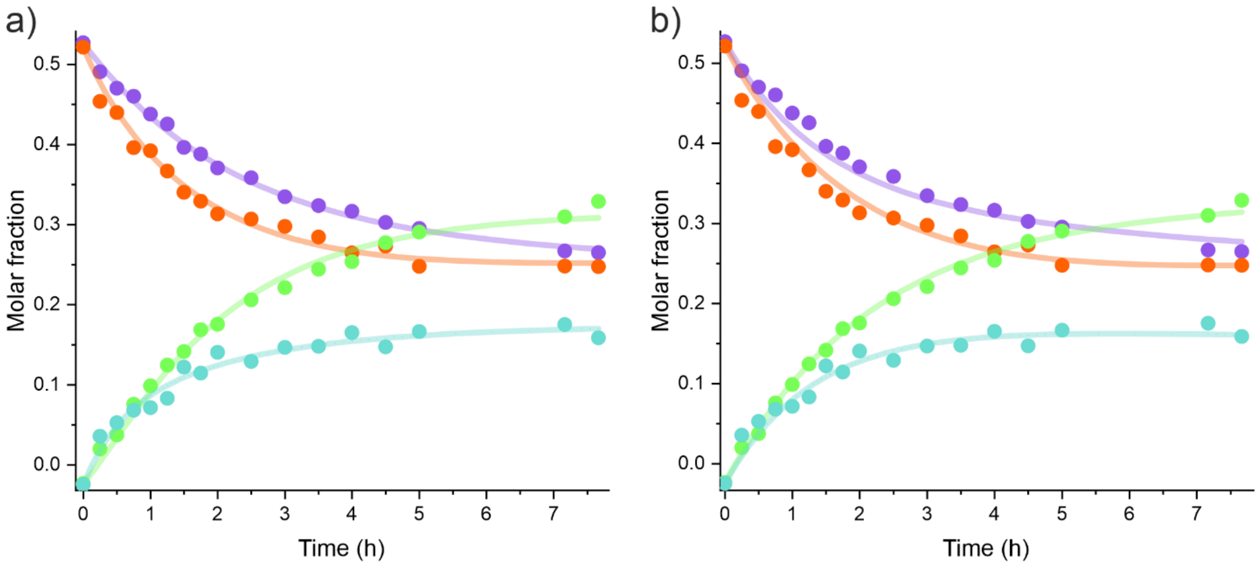

Figure S6. Comparison of different fitted kinetic profiles for Step IV (figure S5) allowing to assign signals corresponding to *P*-*Z*-stable and *M*-*Z*-stable. a) Fitting in the case that *P*-*Z*-stable corresponds to the signal at –97.8 ppm and *M*-*Z*-stable at –98.6 ppm. b) Fitting in the case that *P*-Z-stable corresponds to the signal at –98.6 ppm and M-*Z*-stable at –97.8 ppm. Kinetic profile a) showed a better fit.

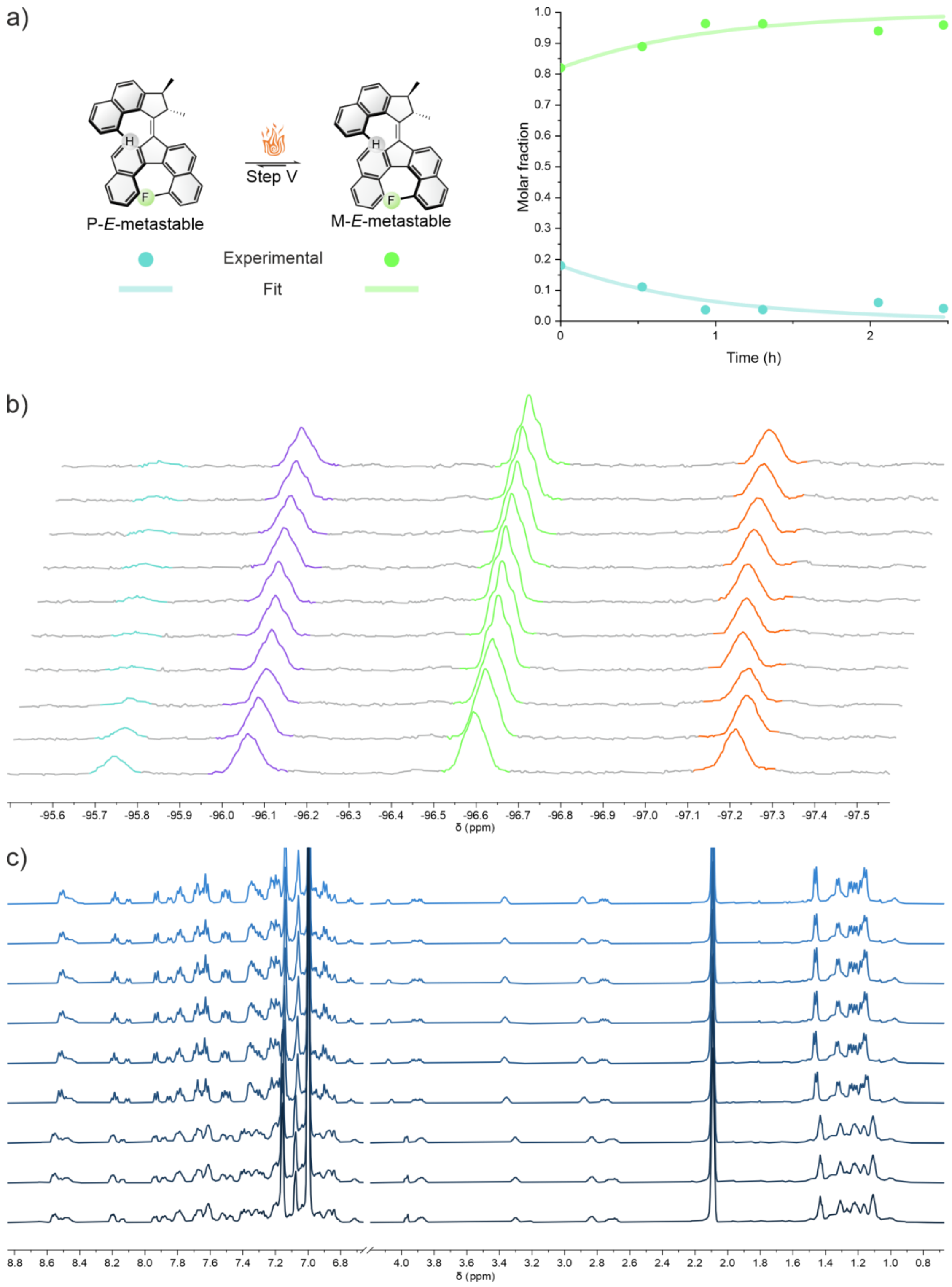

Figure S7. Step V of the rotation cycle. NMR (500 MHz, toluene-d_8, –60 °C) *in*-situ thermal helicene inversion of the previously generated ***P-E*-metastable** (cyan) to ***M-E*-metastable** (green). The (photochemically) unreacted ***M-Z*-stable** (orange) and ***P-Z*-stable** (purple) remains constant during this process. a) Kinetic traces of the evolution of isomers of **M1** obtained by the integration of ^{19}F NMR signals. b) Evolution of the ^{19}F NMR spectra (from bottom to top). c) Evolution of the ^{1}H NMR spectra (from bottom to top).

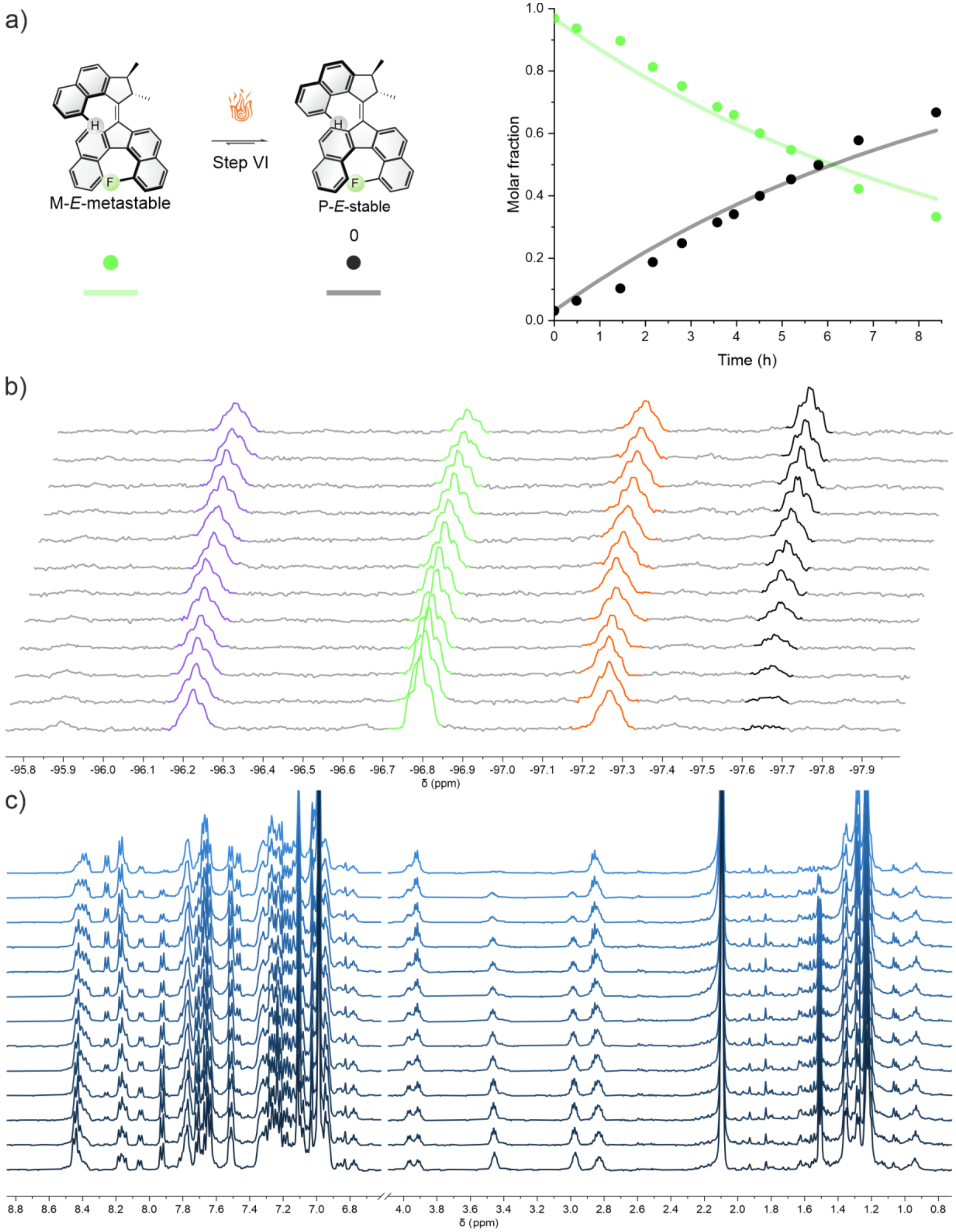

Figure S8. Step VI of the rotation cycle. NMR (500 MHz, toluene-d_8, 15 °C) *in*-situ thermal helix and helicene inversion of the previously generated ***M-E*-metastable** (red) to ***P-E*-stable** (Black). The (photochemically) unreacted ***M-Z*-stable** (orange) and ***P-Z*-stable** (purple) remain constant during this process. a) Kinetic traces of the evolution of isomers of **M1** obtained by the integration of ^{19}F NMR signals. b) Evolution of the ^{19}F NMR spectra (from bottom to top). c) Evolution of the ^{1}H NMR spectra (from bottom to top).

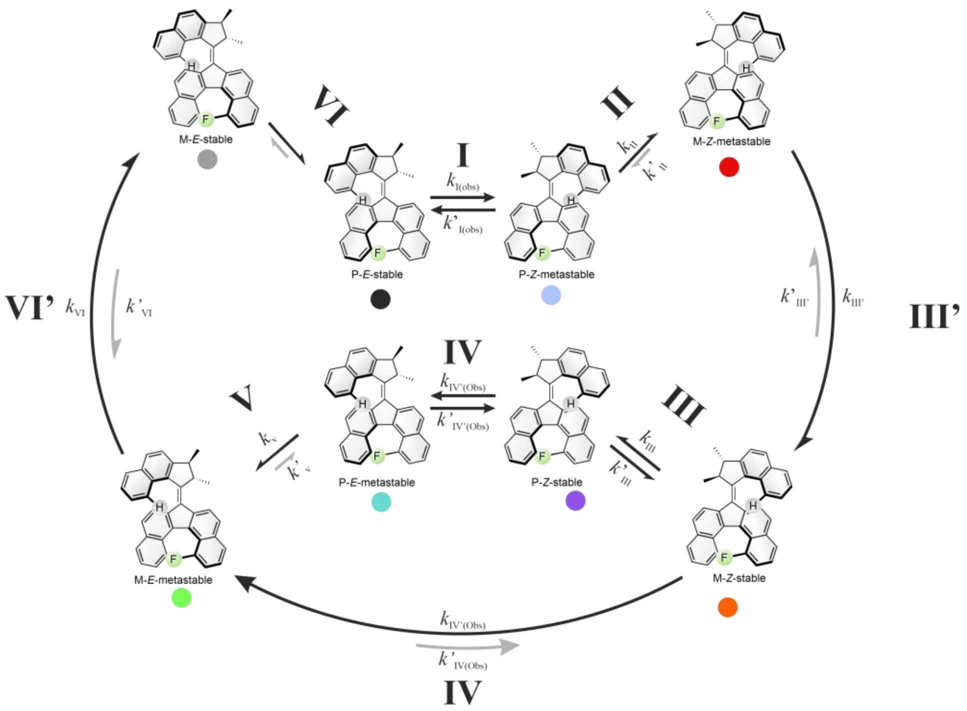

Table S1. Fitted kinetic constants associated to the corresponding step in the rotation cycle of **M1** during the experiments depicted in Figure S1 to Figure S8.

Constant	Reaction	Value (h^{-1})	Temperature (°C)	Std. Dev
$k_{I(Obs)}$	***P-E*-stable → *P-Z*-metastable**	0.38	-85	0.01
$k'_{I(Obs)}$	***P-E*-stable ← *P-Z*-metastable**	0.47	-85	0.02
k_{II}	***P-Z*-metastable → *M*-Z-metastable**	0.28	-70	0.002
k'_{II}	***P-Z*-metastable ← *M-Z*-metastable**	NA	-70	NA
$k_{III'}$	***M-Z*-metastable → *M-Z*-stable**	0.08	15	$3x10^{-4}$
$k'_{III'}$	***M-Z*-metastable ← *M-Z*-stable**	NA	15	NA
k_{III}	***M-Z*-stable → *P-Z*-stable**	0.11	15	0.02
k'_{III}	***M-Z*-stable ← *P-Z*-stable**	0.10	15	0.02
$k_{IV(Obs)}$	***P-Z*-stable → *P-E*-metastabale**	0.35	-85	0.01
$k'_{IV(Obs)}$	***P-Z*-stable ← *P-E*-metastabale**	0.52	-85	0.03
$k_{IV'(Obs)}$	***M-Z*-stable → *M-E-metastable***	0.20	-85	0.008
$k'_{IV'(Obs)}$	***M-Z*-stable ← *M-E-metastable***	0.16	-85	0.01
k_V	***P-E*-metastable → *M-E*-metastable**	0.95	-85	0.11
k'_V	***P-E*-metastable ← *M-E*-metastable**	0.53	-85	0.07
k_V	***P-E*-metastable → *M-E*-metastable**	1.04	-60	0.88
k'_V	***P-E*-metastable ← *M-E*-metastable**	NA	-60	NA
k_{VI}	***M-E*-metastable → *P-E*-stable**	0.10	15	0.002
k'_{VI}	***M-E*-metastable ← *P-E*-stable**	NA	15	NA

3. Exchange spectroscopy (VT-EXSY)

The exchange between the *P* and *M* helicities of the *E*-stable conformers and the exchange between the *P* and *M* helicities of the *Z*-stable conformers of motor **M1** were studied by VT-EXSY spectroscopy.

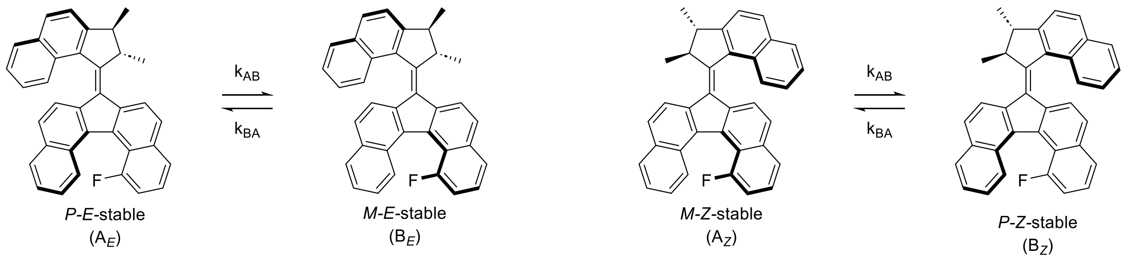

Figure S9: Exchange reactions between the *P* and *M* helicities of the *E*-stable and *Z*-stable conformers of motor **M1**.

The two-site exchange between species A and B, as labeled in Figure S9, is given by equation 1

$$\mathrm{A} \underset{k_{BA}}{\overset{k_{AB}}{\rightleftharpoons}} \mathrm{B} \tag{1}$$

The corresponding populations of species A (P_A) and species B (P_B) are described by equations 2 and 3, respectively.

$$P_A = \frac{k_{BA}}{k_{AB}+k_{BA}} \tag{2}$$

$$P_B = \frac{k_{AB}}{k_{AB}+k_{BA}} \tag{3}$$

By solving the Bloch-McConnel equations it follows that the intensities of the auto peaks (diagonal peaks) I_{AA} and I_{BB} and the intensities of the exchange peaks (cross-peaks) I_{AB} and I_{BA} are given by equations 4a-d.[9]

$$I_{AA} = I_A(0)(-(\lambda_2 - a_{11})e^{-\lambda_1\tau_m} + (\lambda_1 - a_{11})e^{-\lambda_2\tau_m})/(\lambda_1 - \lambda_2) \tag{4a}$$

$$I_{BB} = I_B(0)(-(\lambda_2 - a_{22})e^{-\lambda_1\tau_m} + (\lambda_1 - a_{22})e^{-\lambda_2\tau_m})/(\lambda_1 - \lambda_2) \tag{4b}$$

$$I_{AB} = I_A(0)(a_{21}e^{-\lambda_1\tau_m} - a_{21}e^{-\lambda_2\tau_m})/(\lambda_1 - \lambda_2) \tag{4c}$$

$$I_{BA} = I_B(0)(a_{12}e^{-\lambda_1\tau_m} - a_{12}e^{-\lambda_2\tau_m})/(\lambda_1 - \lambda_2) \tag{4d}$$

In these equations $\lambda_{1,2} = \frac{1}{2}\{(a_{11} + a_{22}) \pm [(a_{11} - a_{22})^2 + 4k_{AB}k_{BA}]^{\frac{1}{2}}\}$, $a_{11} = R_A + k_{AB}$, $a_{12} = -k_{BA}$, $a_{21} = -k_{AB}$ and $a_{22} = R_B + k_{BA}$. R_A and R_B are the longitudinal relaxation rates of magnetization for A and B, and $I_A(0)$ and $I_B(0)$ denote the amount of longitudinal magnetization associated with states A and B at the start of the mixing period τ_m.

The activation parameters $\Delta H^{\ddagger}$ and $\Delta S^{\ddagger}$ for the exchange reactions can be obtained directly from the temperature dependence of the reaction rates (k) using the following Eyring equation:

$$k = \frac{k_B T}{h} e^{-\frac{\Delta G^{\ddagger}}{RT}} = \frac{k_B T}{h} e^{-\frac{\Delta H^{\ddagger}}{RT} + \frac{\Delta S^{\ddagger}}{R}} \tag{5}$$

Alternatively, this equation can be rewritten in its linearized form, according to equation 6.

$$\ln\frac{kh}{k_B T} = -\frac{\Delta H^{\ddagger}}{R}\frac{1}{T} + \frac{\Delta S^{\ddagger}}{R} \tag{6}$$

Plotting $ln\frac{kh}{k_B T}$ versus 1/T allows one to perform a linear regression on the data to obtain the enthalpy of activation $\Delta H^{\ddagger}$ from the negative slope $-\Delta H^{\ddagger}/R$ and the entropy of activation $\Delta S^{\ddagger}$ form the y-intercept $\Delta S^{\ddagger}/R$.

The Gibbs free energy of activation $\Delta G^{\ddagger}$ at a temperature T can be calculated from the enthalpy and entropy of activation using equation 7.

$$\Delta G^{\ddagger} = \Delta H^{\ddagger} - T\Delta S^{\ddagger} \tag{7}$$

Hence, NMR experiments were performed on a Varian Inova 500 MHz (^{1}H NMR frequency) spectrometer. An NMR sample containing a ~ 1:1 mixture of *E*-stable and *Z*-stable in toluene-d_8 was prepared (ca. 10 mM). This allowed the simultaneous analysis of both sets of exchanging species in a single set of experiments. The chemical exchange between states A and B was monitored by recording a series of 2D homonuclear ^{19}F NOESY spectra with mixing times of 0.001, 0.005, 0.01, 0.015, 0.02, 0.03, 0.04, 0.06, 0.08, 0.12, 0.16, 0.32, 0.48, 0.64, 0.96, 1.28 and 1.92 s at 6 different set temperatures of 5, 10, 15, 20, 25 and 30 °C, respectively. A spectral window of 2353 Hz was employed and 4 scans were acquired for each experiment. Figure S10 (left) shows a 1D ^{19}F NMR spectrum of the mixture at 25 °C, and Figure S10 (right) shows a 2D ^{19}F NOESY spectrum of the same mixture with clearly visible auto peaks and exchange peaks.

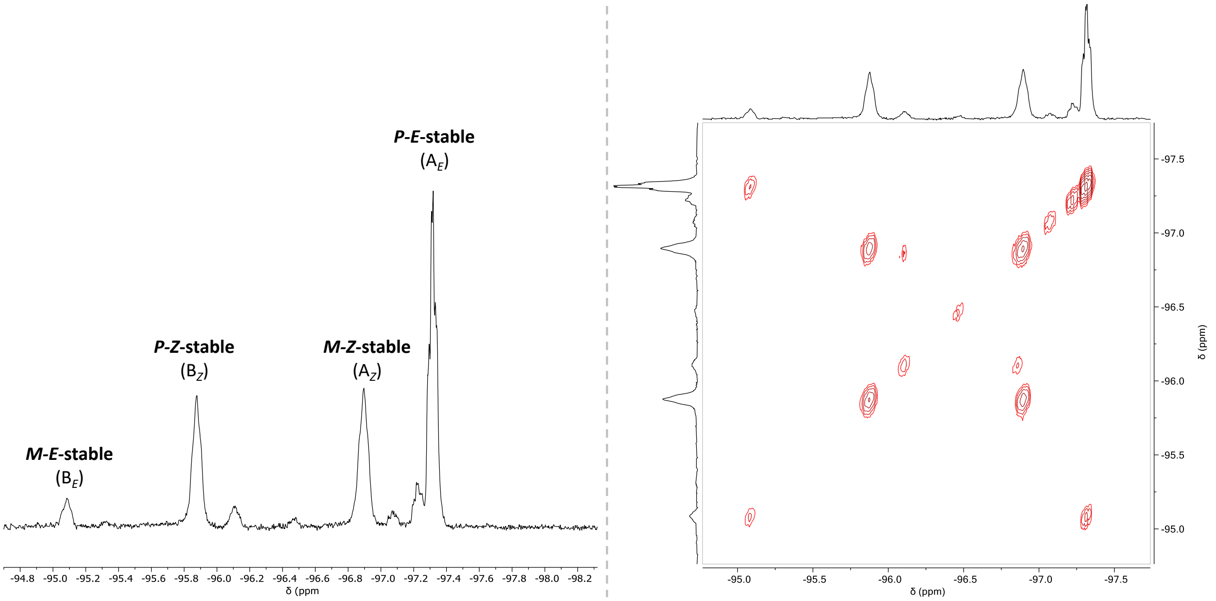

Figure S10: Left: ^{19}F NMR spectrum of the mixture of isomers of motor **M1** (470MHz, toluene-d_8, 25 °C). Right: 2D ^{19}F NOESY spectrum of the same mixture (470MHz, toluene-d_8, 25 °C, 440 ms mixing time).

E isomers

The diagonal peaks and the cross peaks were integrated and their intensity was plotted at each temperature as a function of the mixing time to obtain the buildup curves shown in Figure S11. These curves were simultaneously fitted using equations 4a-4d. Population ratios P_A/P_B were determined from 1D ^{19}F NMR spectra and these population ratios were used to constrain the ratio of k_{BA}/k_{AB} in the modelling procedure. The measured population ratios as well as the calculated rate constants and relaxation rates at each temperature are summarized in Table S2.

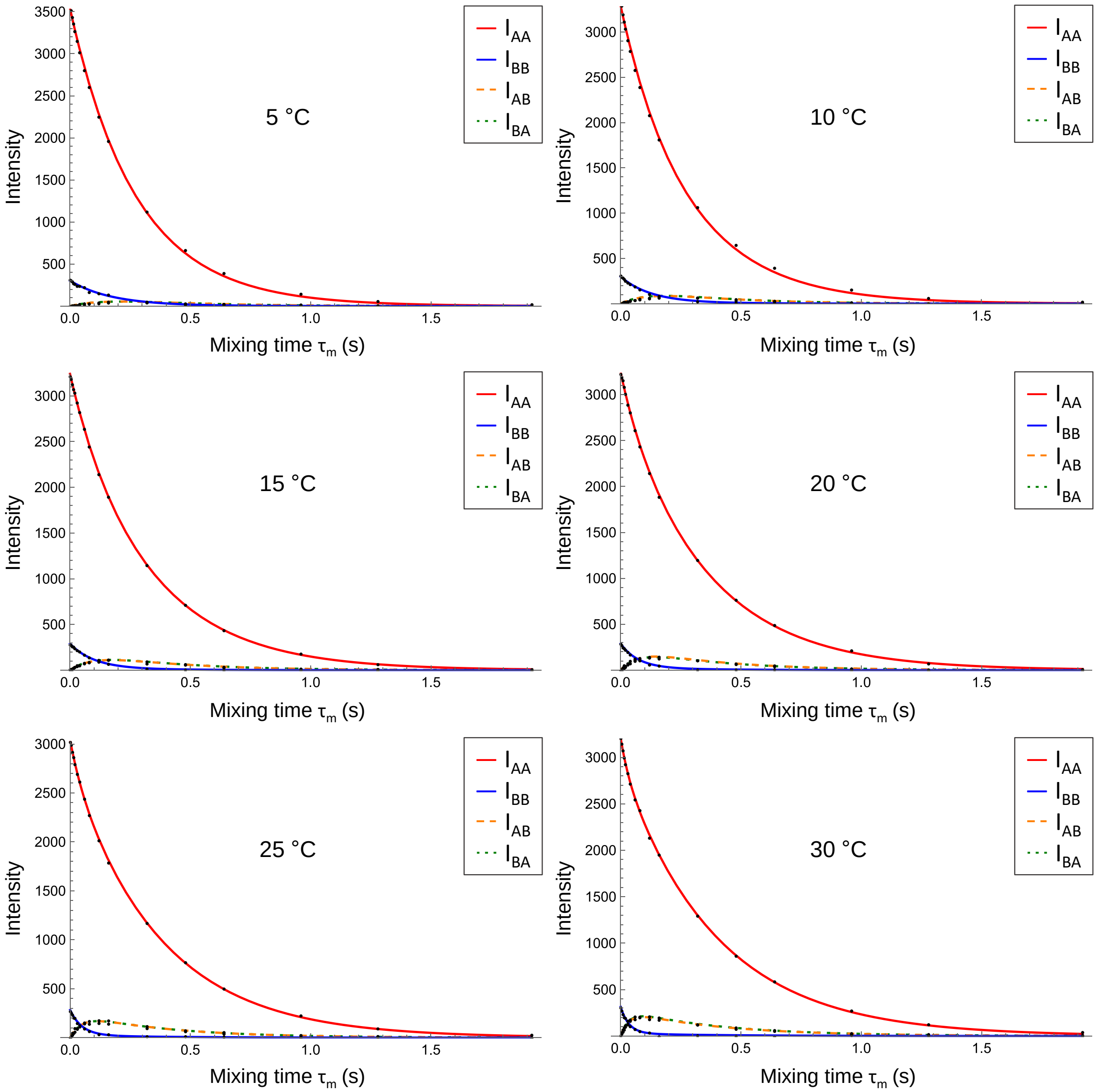

Figure S11: Buildup curves for the exchange between *P*-*E*-stable and M-*E*-stable showing the intensities of the diagonal peaks and cross-peaks as a function of mixing time τ_m at the different temperatures.

Table S2: Summary of the measured population ratios (P_A/P_B) as well as the calculated rate constants (k_{AB} and k_{BA}) and relaxation rates (R_A and R_B) at the different temperatures for the exchange between *P-E*-stable and *M-E*-stable. Standard errors are given in parentheses.

T (°C)	P_A/P_B	k_{AB} (s^{-1})	k_{BA} (s^{-1})	R_A (s^{-1})	R_B (s^{-1})
5	11.69	0.19 (0.03)	2.6 (0.4)	3.49 (0.04)	3.5 (0.6)
10	11.32	0.39 (0.05)	4.4 (0.6)	3.39 (0.06)	3.3 (0.8)
15	11.44	0.57 (0.02)	6.5 (0.2)	3.00 (0.02)	2.9 (0.3)
20	10.76	0.94 (0.04)	10.1 (0.4)	2.87 (0.04)	2.5 (0.5)
25	10.78	1.53 (0.06)	16.5 (0.6)	2.73 (0.05)	2.3 (0.6)
30	10.66	2.31 (0.08)	24.7 (0.8)	2.60 (0.07)	1.8 (0.8)

The obtained rate constants for the forward (k_{AB}) and backward (k_{BA}) reactions were used to plot $\ln(k \cdot h)/(k_B \cdot T)$ against 1/T and the data was fitted using the linearized Eyring equation (equation 6), see Figure S12. The obtained activation parameters and the corresponding values at 20 °C of the Gibbs free energy of activation, half-life and lifetime are summarized in Table S3.

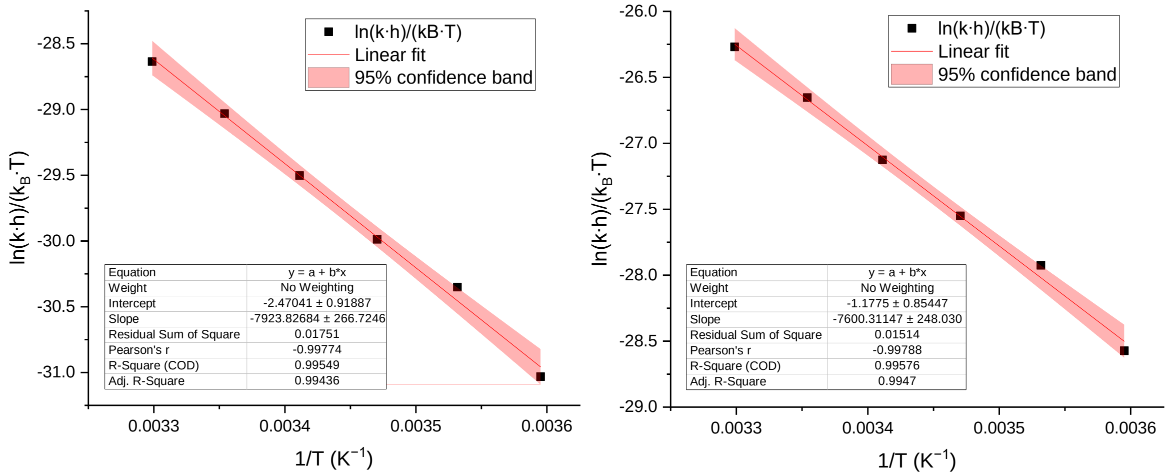

Figure S12: Eyring pots for the exchange reactions from *P-E*-stable to *M-E*-stable (A → B, left) and *M-E*-stable to *P-E*-stable (B → A, right).

Table S3: Calculated activation parameters corresponding to the exchange reactions from *P-E*-stable to *M-E*-stable (A → B) and *M-E*-stable to *P-E*-stable (B → A). Standard errors are given in parentheses.

	$\Delta H^‡$ ($kJ \cdot mol^{-1}$)	$\Delta S^‡$ ($J \cdot mol^{-1} \cdot K^{-1}$)	$\Delta G^‡$ (20 °C) ($kJ \cdot mol^{-1}$)	$t_{1/2}$ (20 °C) (s)	τ (20 °C) (s)
**A → B**	66 (2)	−21(8)	71.9 (0.2)	0.74	1.1
**B → A**	63 (2)	−10 (7)	66.1 (0.2)	6.7×10^{-2}	9.7×10^{-2}

Z isomers

The same procedure that was used for the pair of *E* isomers was applied to the pair of *Z* isomers. Hence, the diagonal peaks and the cross peaks were integrated and their intensity was plotted at each temperature as a function of the mixing time to obtain the buildup curves shown in Figure S13. These curves were simultaneously fitted using equations 4a-4d. Population ratios P_A/P_B were determined from 1D ^{19}F NMR spectra and these population ratios were used to constrain the ratio of k_{BA}/k_{AB} in the modelling procedure. The measured population ratios as well as the calculated rate constants and relaxation rates at each temperature are summarized in Table S4.

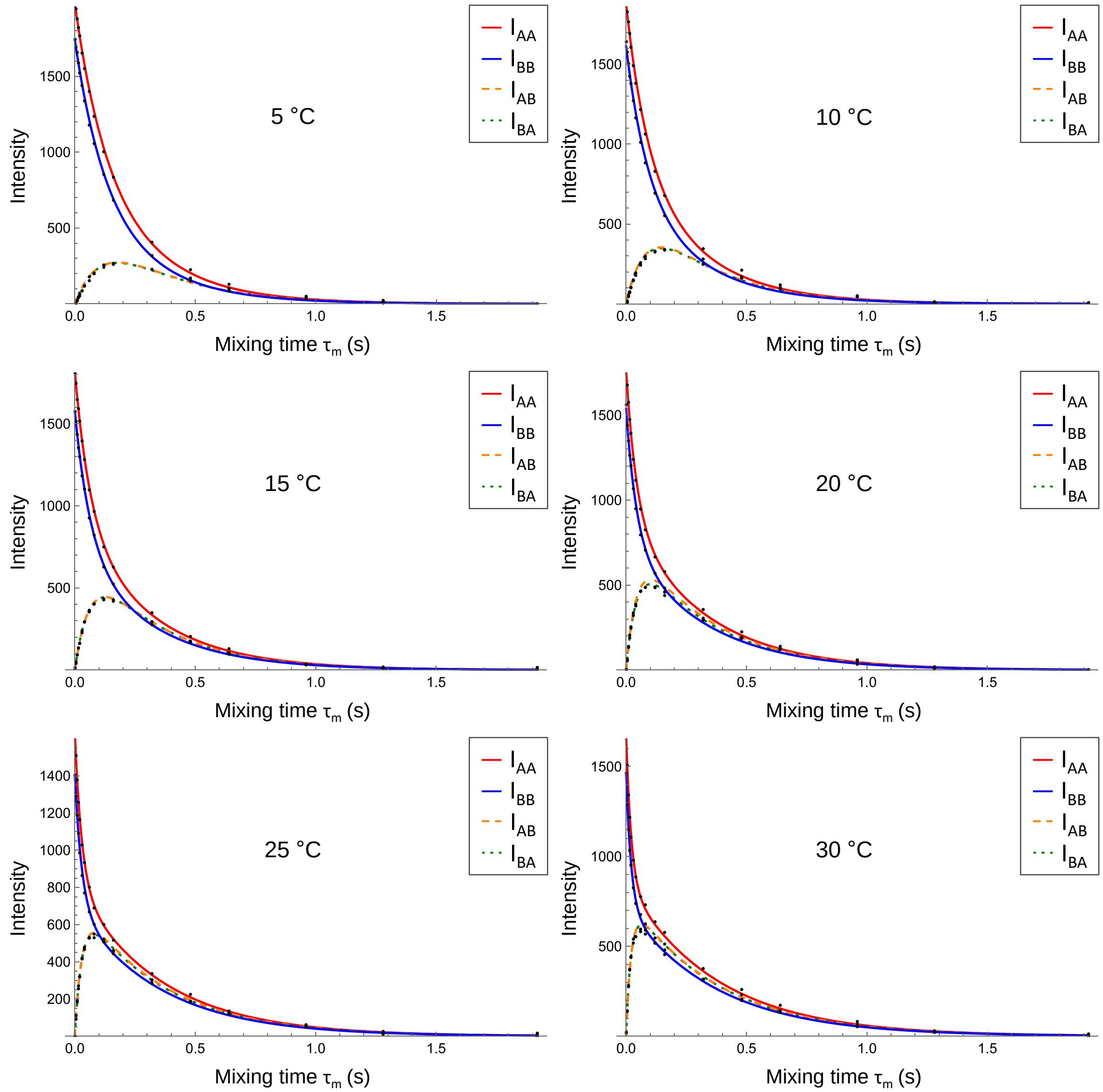

Figure S13: Buildup curves for the exchange between *M*-*Z*-stable and *P*-*Z*-stable showing the intensities of the diagonal peaks and cross-peaks as a function of mixing time τ_m at the different temperatures.

Table S4: Summary of the measured population ratios (P_A/P_B) as well as the calculated rate constants (k_{AB} and k_{BA}) and relaxation rates (R_A and R_B) at the different temperatures for the exchange between *M-Z*-stable and *P-Z*-stable isomers. Standard errors are given in parentheses.

T (°C)	P_A/P_B	k_{AB} (s^{-1})	k_{BA} (s^{-1})	R_A (s^{-1})	R_B (s^{-1})
5	1.13	2.18 (0.07)	2.46 (0.07)	3.60 (0.08)	3.7 (0.1)
10	1.15	3.7 (0.1)	4.3 (0.1)	3.7 (0.1)	3.5 (0.2)
15	1.15	5.80 (0.06)	6.67 (0.07)	3.50 (0.07)	2.99 (0.08)
20	1.09	9.4 (0.2)	10.2 (0.2)	3.3 (0.2)	2.9 (0.2)
25	1.14	14.6 (0.2)	16.6 (0.3)	3.7 (0.2)	1.9 (0.3)
30	1.15	20.7 (0.5)	23.7 (0.6)	3.8 (0.5)	1.4 (0.5)

The obtained rate constants for the forward (k_{AB}) and backward (k_{BA}) reactions were used to plot $\ln(k \cdot h)/(k_B \cdot T)$ against 1/T and the data was fitted using the linearized Eyring equation (equation 6), see Figure S14. The obtained activation parameters and the corresponding values at 20 °C of the Gibbs free energy of activation, half-life and lifetime are summarized in Table S5.

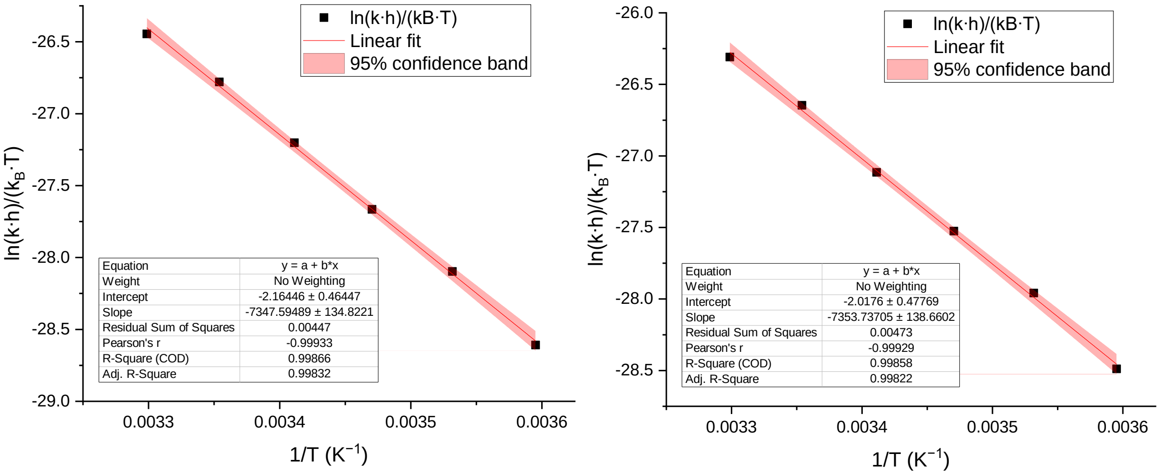

Figure S14: Eyring pots for the exchange reactions from *M-Z*-stable to *P-Z*-stable (A → B, left) and *P-Z*-stable to *M-Z*-stable (B → A, right).

Table S5: Calculated activation parameters corresponding to the exchange reactions from *M-Z*-stable to *P-Z*-stable (A → B) and *P-Z*-stable to *M-Z*-stable (B → A). Standard errors are given in parentheses

	$\Delta H^{\ddagger}$ ($kJ \cdot mol^{-1}$)	$\Delta S^{\ddagger}$ ($J \cdot mol^{-1} \cdot K^{-1}$)	$\Delta G^{\ddagger}$ (20 °C) ($kJ \cdot mol^{-1}$)	$t_{1/2}$ (20 °C) (s)	τ (20 °C) (s)
**A → B**	61 (1)	−18 (4)	66.4 (0.1)	7.6 x 10^{-2}	0.11
**B → A**	61 (1)	−17(4)	66.1 (0.1)	6.7 x 10^{-2}	9.7 x 10^{-2}

4. Variable-temperature UV-vis spectroscopy

All variable-temperature UV-vis experiments were performed on (2*S*,3*S*)-**M1**. As two helixes are present in the compound, the corresponding stereodescriptors were color-coded, in green for the helicity of the lower helicene part, and in orange for the helicity of the upper half of the overcrowded-alkene.

Due to consequent drifting induced by the thermal equilibration of the compounds, the first few spectra of thermal decays were omitted.

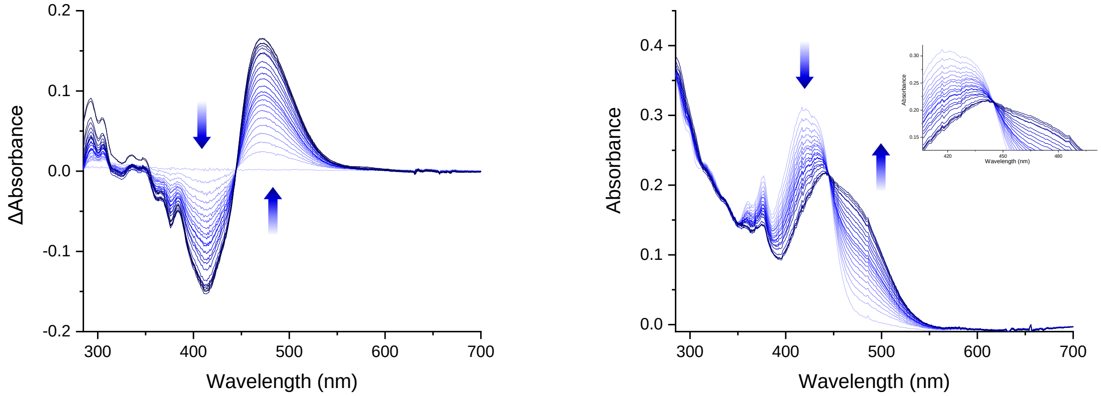

Figure S15. UV-vis monitoring (toluene, ~25 µM, –85 °C) of the irradiation of a highly enriched sample of (2*S*,3*S*)-(*P*,*M*)-*E*-**M1** with 365 nm UV light, yielding metastable (2*S*,3*S*)-(*P*,*P*)-*Z*-**M1** (step I). Left: difference absorption spectra. Right: UV-vis spectra.

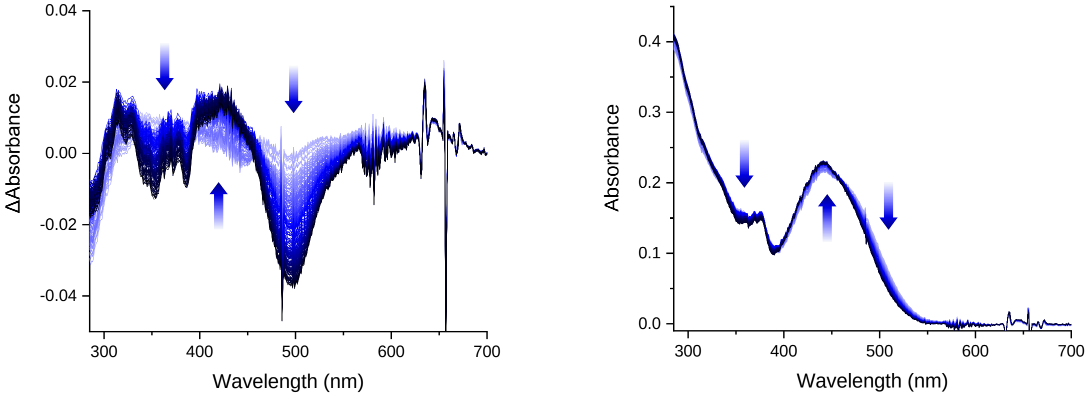

Figure S16. UV-vis monitoring (toluene, ~25 µM, –65 °C) of the helicene inversion of the dibenzofluorene bottom half resulting in the conversion of (2*S*,3*S*)-(*P*,*P*)-*Z*-**M1** in (2*S*,3*S*)-(*M*,*P*)-*Z*-**M1** (step II). Left: difference absorption spectra, right: UV-vis spectra.

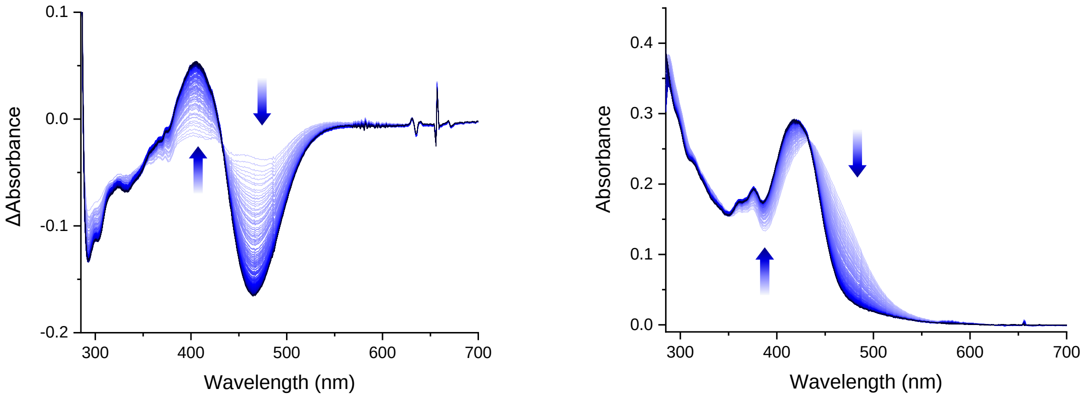

Figure S17. UV-vis monitoring (toluene, ~25 µM, 35 °C) of the conversion of metastable (2*S*,3*S*)-(*M*,*P*)-*Z*-**M1** in stable (2*S*,3*S*)-(*M*,*M*)-*Z*-**M1** and (2*S*,3*S*)-(*P*,*M*)-*Z*-**M1** through a THI of the upper half followed by the equilibration of both helicities of the bottom half, happening readily at this temperature (steps III' and III). Left: difference absorption spectra. Right: UV-vis spectra.

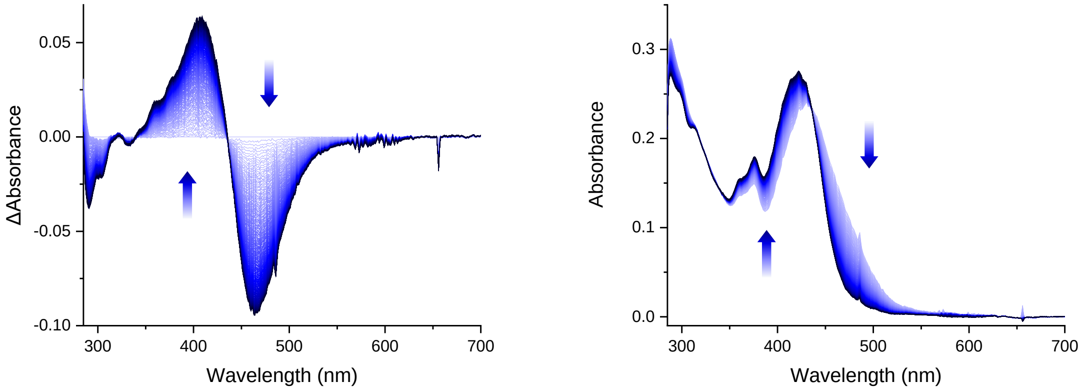

Figure S18. UV-vis monitoring (toluene, ~30 µM, 30 °C) of the conversion of metastable (2*S*,3*S*)-(*P*,*P*)-*Z*-**M1** in (2*S*,3*S*)-(*M*,*M*)-*Z*-**M1** and (2*S*,3*S*)-(*P*,*M*)-*Z*-**M1** through successive helicene inversion of the lower half (step II) followed by a THI of the upper half of the motor (step III') and successive helicene inversion of the lower half (step III). The same process is presented Figure S17 where the data was acquired after recording the two previous steps under cryogenic conditions (baseline at –85 °C), resulting in a large thermal drift, requiring significant processing. For this control experiment, both baseline spectrum and thermal decay were recorded at 30 °C. The observed changes are the same as presented Figure S17 following the cryogenic temperature experiments, thus proving the accuracy of the previously used methodology. Left: difference absorption spectra. Right: UV-vis spectra.

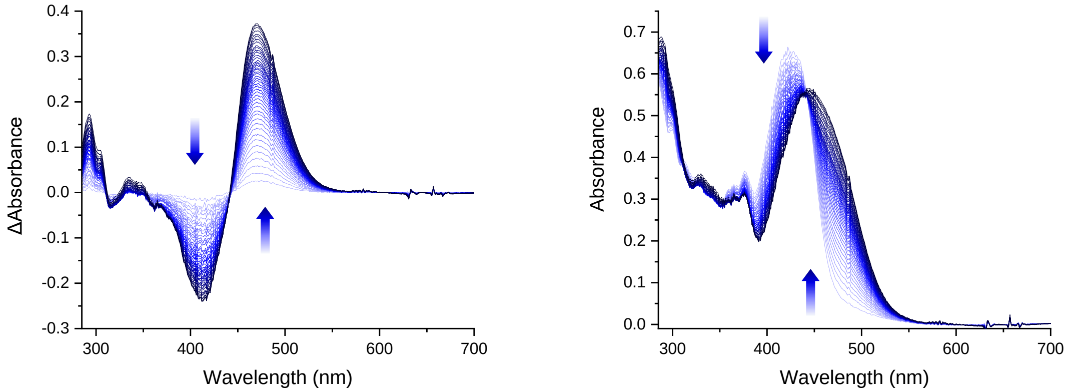

Figure S19. UV-vis monitoring (toluene, ~30 µM, –85 °C) of the irradiation of a highly enriched sample containing stable (2*S*,3*S*)-(*P*,*M*)-*Z*-**M1** and stable (2*S*,3*S*)-(*M*,*M*)-*Z*-**M1** in a 1:1 ratio with 365 nm UV light, yielding respectively metastable states (2*S*,3*S*)-(*P*,*P*)-*E*-**M1** and (2*S*,3*S*)-(*M*,*P*)-*E*-**M1** (step IV). Left: difference absorption spectra. Right: UV-vis spectra.

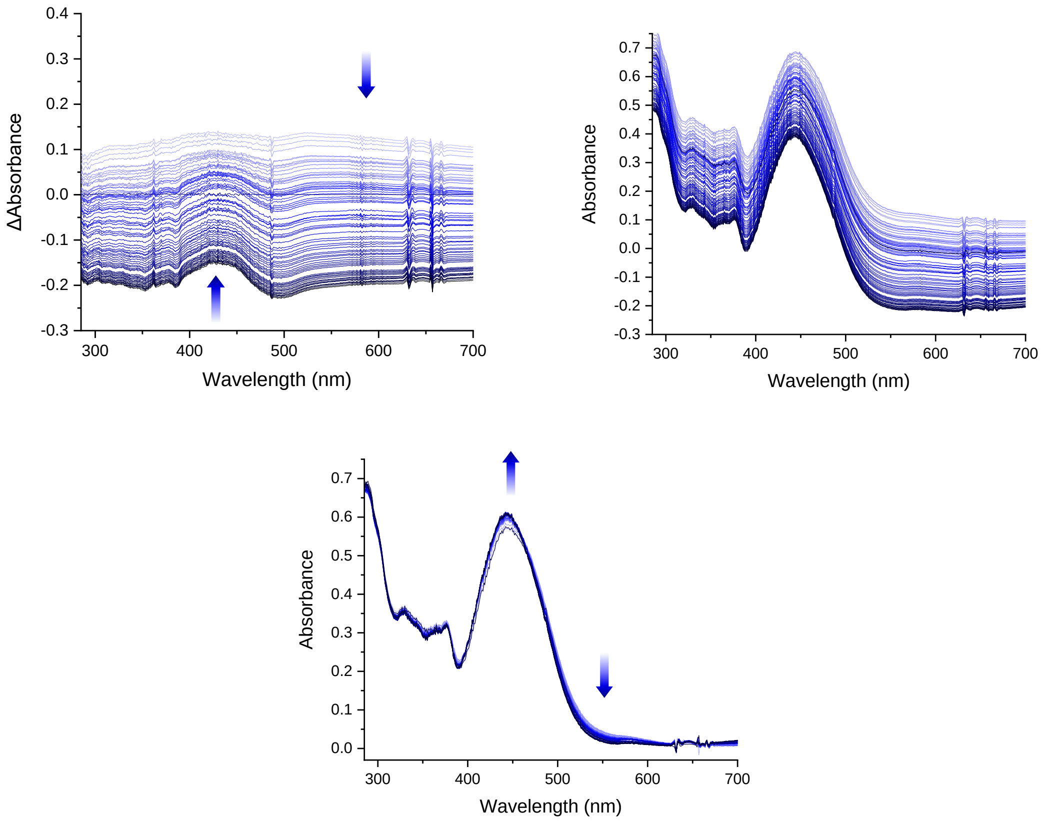

Figure S20. UV-vis monitoring (toluene, ~30 µM, –65 °C) of helicene inversion of the dibenzofluorene bottom half resulting in the conversion of metastable (2*S*,3*S*)-(*P*,*P*)-*E*-**M1** in metastable (2*S*,3*S*)-(*M*,*P*)-*E*-**M1** (step V). The initial sample already contained ~50% of metastable (2*S*,3*S*)-(*M*,*P*)-*E*-**M1** from the previous step. This transformation resulted in a very small change of the characteristic features of the UV-vis spectrum, within a short timescale, resulting in a preponderance of the thermal drift in the obtained data. a) Difference absorption spectra. b) UV-vis spectra, c) UV-vis spectra after baseline corrections.

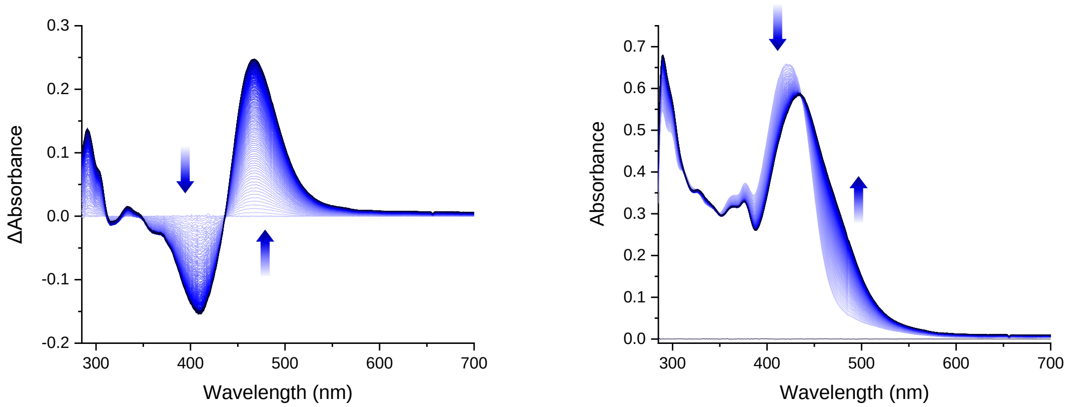

Figure S21. UV-vis monitoring (toluene, ~30 µM, 0 °C) of the irradiation of a highly enriched sample containing (2*S*,3*S*)-(*P*,*M*)-*Z*-**M1** and (2*S*,3*S*)-(*M*,*M*)-*Z*-**M1** in a 1:1 ratio with 365 nm UV light, yielding metastable (2*S*,3*S*)-(*M*,*P*)-*E*-**M1** (concerted step IV and step V). Upon illumination, (2*S*,3*S*)-(*P*,*P*)-*E*-**M1** is also generated, but readily converts to (2*S*,3*S*)-(*M*,*P*)-*E*-**M1** at this temperature (step V). Left: difference absorption spectra. Right: UV-vis spectra.

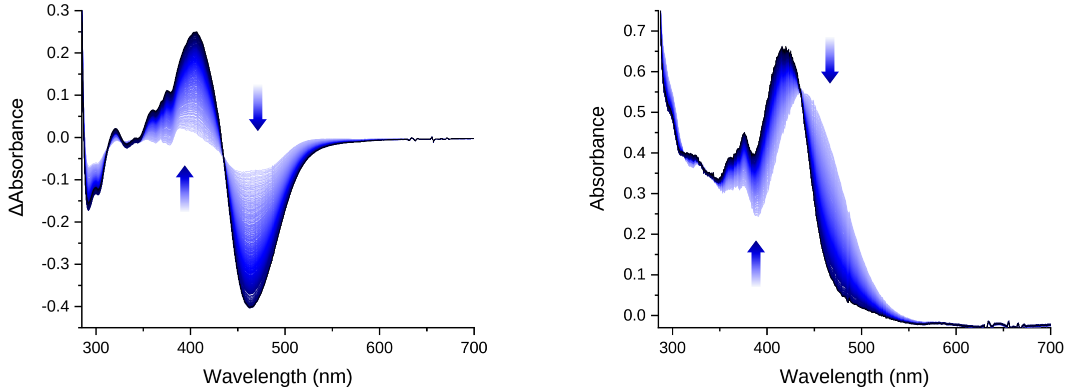

Figure S22. UV-vis monitoring (toluene, ~30 µM, 30 °C) of the conversion of metastable (2*S*,3*S*)-(*M*,*P*)-*E*-**M1** in stable (2*S*,3*S*)-(*P*,*M*)-*E*-**M1** through a THI of the upper half of the motor (step VI') followed by a helicene inversion of the lower half (step VI). Left: difference absorption spectra. Right: UV-vis spectra.

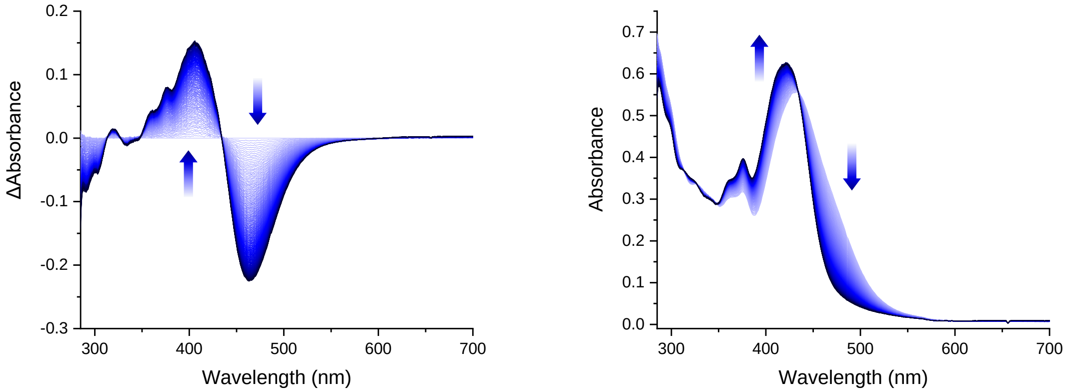

Figure S23. UV-vis monitoring (toluene, ~30 µM, 30 °C) of the conversion of metastable (2*S*,3*S*)-(*M*,*P*)-*E*-**M1** in stable (2*S*,3*S*)-(*P*,*M*)-*E*-**M1** through a THI of the upper half of the motor (step VI') followed by a helicene inversion of the lower half (step VI). The same process is presented Figure S22 where the data was acquired after recording the two previous steps under cryogenic conditions (baseline at –85 °C), resulting in a large thermal drift, requiring significant processing. For this control experiment, both baseline spectrum and thermal decay were recorded at 30 °C. The observed changes are the same as presented in Figure S22 following the cryogenic temperature experiments, thus proving the accuracy of the previously used methodology. Left: difference absorption spectra. Right: UV-vis spectra.

5. Variable-temperature circular dichroism

All variable-temperature UV-vis experiments were performed on (2*S*,3*S*)**-M1**. As two helixes are present in the compound, the corresponding stereodescriptors were color-coded, in green for the helicity of the lower helicene part, and in orange for the helicity of the upper half of the overcrowded-alkene. Due to consequent drifting induced by the thermal equilibration of the compounds, the first few spectra of thermal decays were omitted.

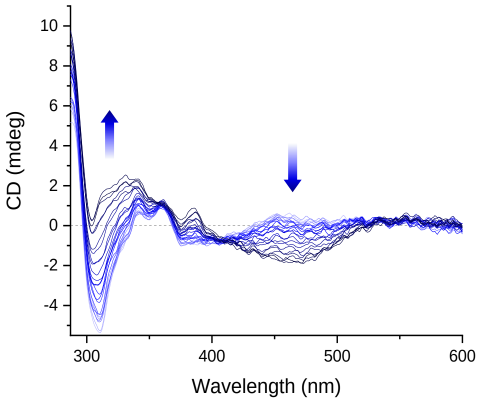

Figure S24. CD monitoring (toluene, ~25 µM, –85 °C) of the irradiation of a highly enriched sample of (2*S*,3*S*)-(*P*,*M*)-*E*-**M1** with 365 nm UV light, yielding metastable (2*S*,3*S*)-(*P*,*P*)-*Z*-**M1** (step I).

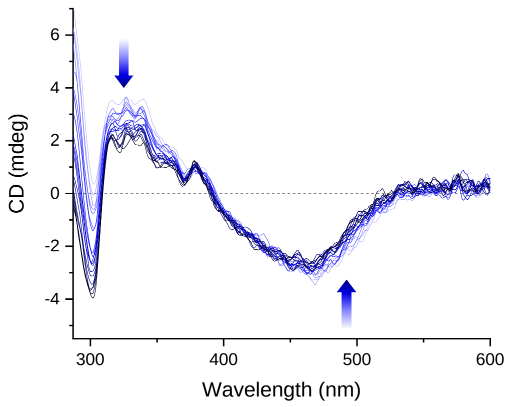

Figure S25. CD monitoring (toluene, ~25 µM, –65 °C) of the helicene inversion of the dibenzofluorene bottom half resulting in the conversion of (2*S*,3*S*)-(*P*,*P*)-*Z*-**M1** in (2*S*,3*S*)-(*M*,*P*)-*Z*-**M1** (step II).

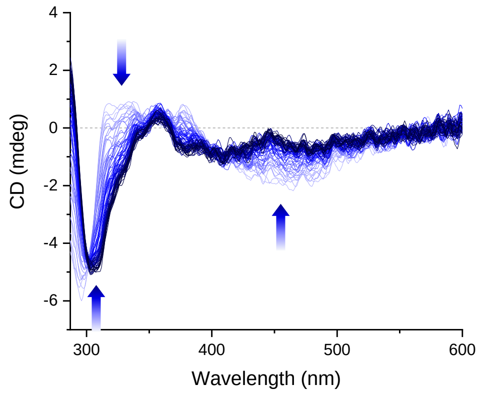

Figure S26. CD monitoring (toluene, ~25 µM, 30 °C) of the conversion of metastable (2*S*,3*S*)-(*M*,*P*)-*Z*-**M1** in stable (2*S*,3*S*)-(*M*,*M*)-*Z*-**M1** (step III’) and (2*S*,3*S*)-(*P*,*M*)-*Z*-**M1** through a THI of the upper half followed by the equilibration of both helicities of the bottom half, happening readily at this temperature (step III).

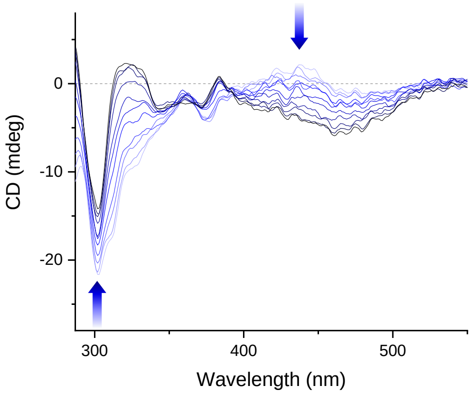

Figure S27. CD monitoring (toluene, ~30 µM, –85 °C) of the irradiation of a highly enriched sample containing (2*S*,3*S*)-(*P*,*M*)-*Z*-**M1** and (2*S*,3*S*)-(*M*,*M*)-*Z*-**M1** in a 1:1 ratio with 365 nm UV light, yielding respectively metastable states (2*S*,3*S*)-(*P*,*P*)-*E*-**M1** and (2*S*,3*S*)-(*M*,*P*)-*E*-**M1** (step IV).

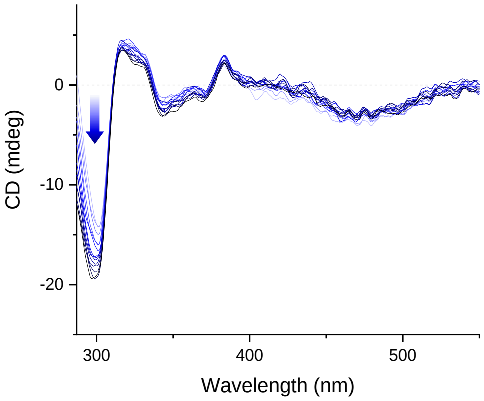

Figure S28. CD monitoring (toluene, ~30 µM, –65 °C) of the helicene inversion of the dibenzofluorene bottom half resulting in the conversion of (2*S*,3*S*)-(*P*,*P*)-*E*-**M1** in (2*S*,3*S*)-(*M*,*P*)-*E*-**M1** (step V). The initial sample already contained ~50% of (2*S*,3*S*)-(*M*,*P*)-*E*-**M1** from the previous step.

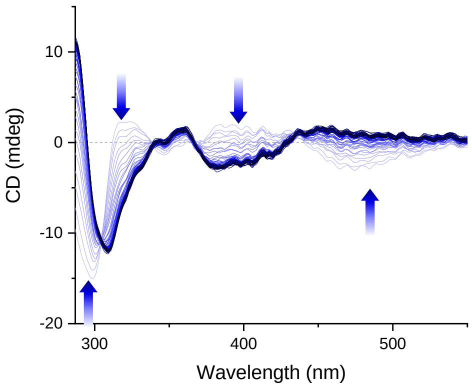

Figure S29. CD monitoring (toluene, ~30 µM, 30 °C) of the conversion of metastable (2*S*,3*S*)-(*M*,*P*)-*E*-**M1** in stable (2*S*,3*S*)-(*P*,*M*)-*E*-**M1** through a THI of the upper half of the motor (step VI') followed by a helicene inversion of the lower half (step VI).

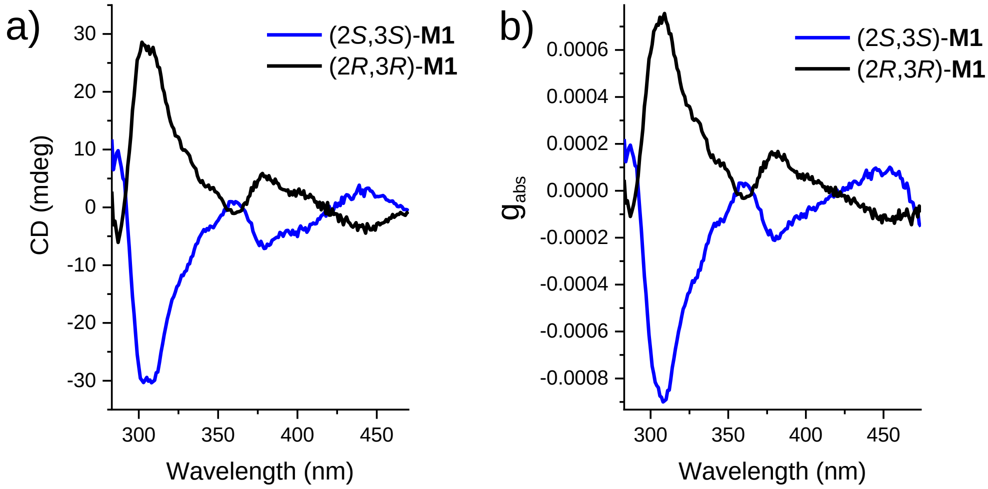

Figure S30. a) Unprocessed CD spectra of (2*R*,3*R*)-**M1** (black) and (2*S*,3*S*)-**M1** (blue) (toluene, ~50 µM, 20 °C). Both samples are composed of isomeric mixtures (*E*/*Z* and stable/metastable isomers) as synthesized. b) CPL absorption dissymmetry factors (g_{abs} = Δε/ε) obtained from the CD spectra presented in panel a). It is important to note that the studied samples are not enantiopure but highly enantioenriched and are composed of mixtures of isomers whose ratio can vary depending on the handling and measurement conditions (light sources in CD and UV/Vis spectrophotometers), thus only the order of magnitude of the g_{abs} can be extracted from this figure.

6. Computational analysis

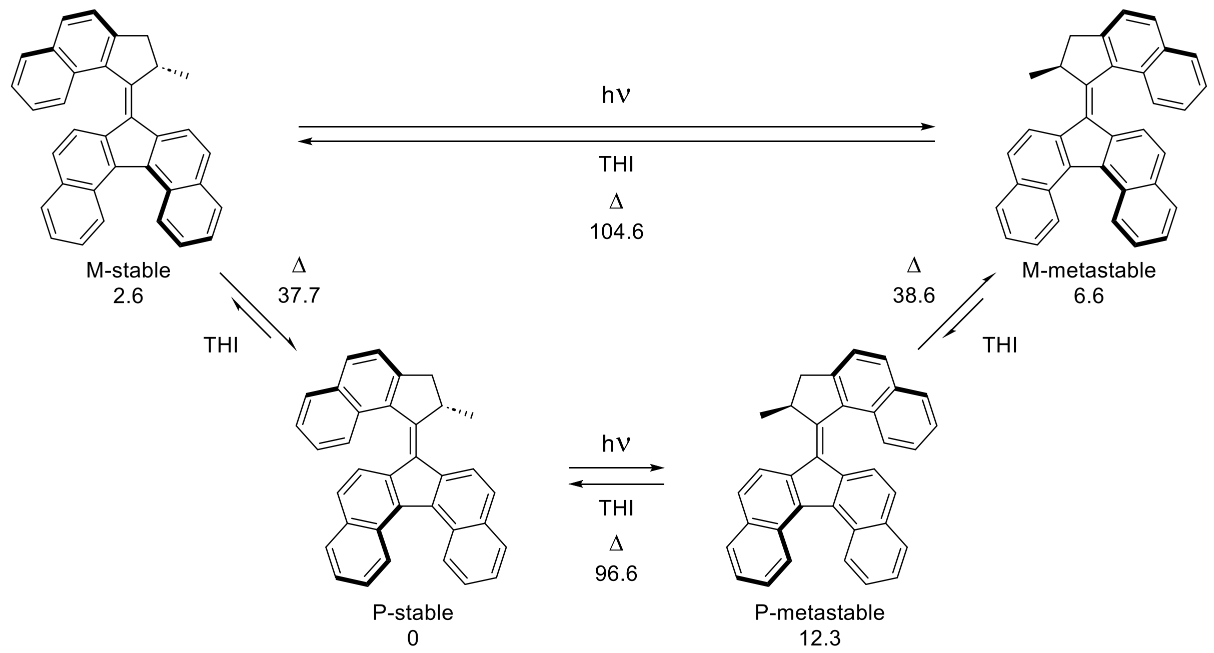

Figure S31. Calculated rotation cycle for the previously reported[10] dibenzofluorene-based molecular motor (*S*- enantiomer) at the r²SCAN-3c/CPCM(Toluene) level of theory. Gibbs free energies are given in kJ/mol at 25 °C. Transition state free energies are given relatively to the corresponding metastable state.

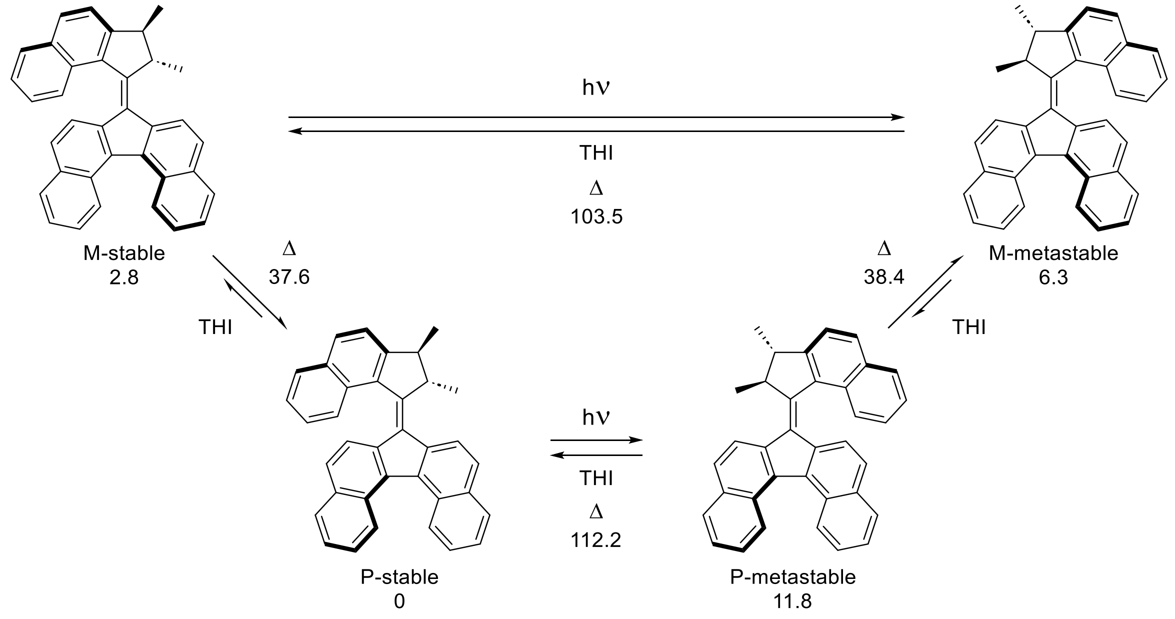

Figure S32. Calculated rotation cycle for the dimethylated analogue of the previously reported dibenzofluorene-based molecular motor (2*S*,3*S*)-**M0** at the r²SCAN-3c/CPCM(Toluene) level of theory. Gibbs free energies are given in kJ/mol at 25 °C. Transition state free energies are given relatively to the corresponding metastable state.

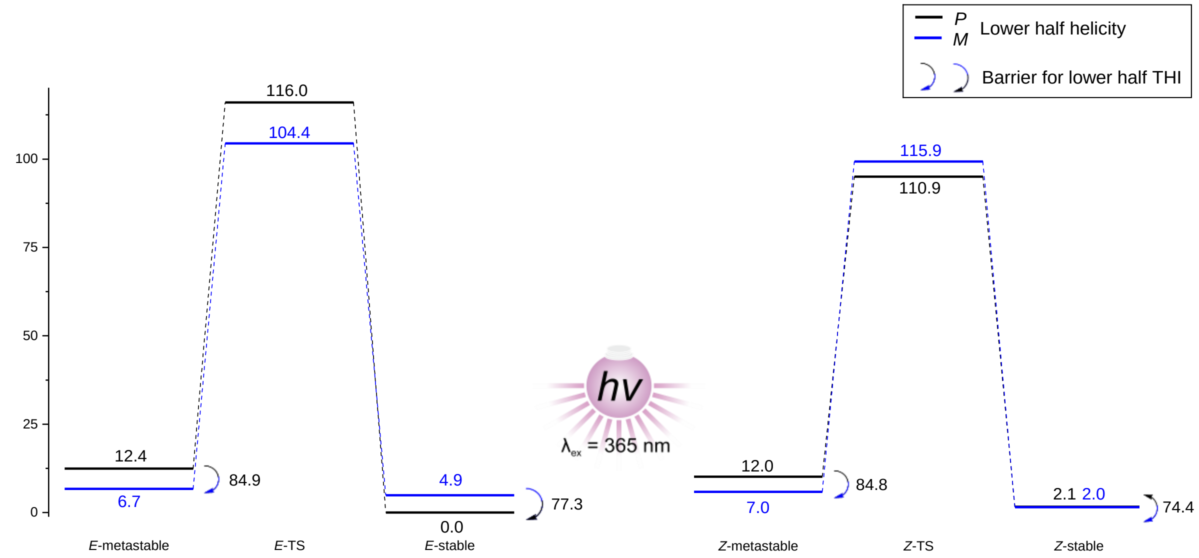

Figure S33. Calculated rotation mechanism for (2S,3*S*)-**M1** at r^{2}SCAN-3c/CPCM(Toluene) level of theory. Gibbs free energies are given in kJ/mol at 25 °C. Curved arrows indicate the favored direction and transition state free energies for the thermal helix inversions of the helicoidal bottom half.

Table S6. Calculated Gibbs free energy values for the rotation cycle of **M1** at various temperatures used for the experimental study. Calculations performed at the r^{2}SCAN-3c/CPCM(Toluene) level of theory. Energies are given in kJ/mol, relative to the most stable isomer.

		ΔG_{calc}(T)						
**Name**	**T**	–85 °C	–70 °C	–65 °C	0 °C	15 °C	25 °C	30 °C
(2*S*,3*S*)-(*M*,*P*)-*E*-**M1**	*M*-E_{ms}	6.8	6.8	6.8	6.7	6.7	6.7	6.7
(2*S*,3*S*)-(*M*,*M*)-*E*-**M1**	*M*-E_s	5.0	5.0	5.0	4.9	4.9	4.9	4.9
(2*S*,3*S*)-(*M*,*P*)-*Z*-**M1**	*M*-Z_{ms}	7.0	7.0	7.0	7.0	7.0	7.0	7.0
(2*S*,3*S*)-(*M*,*M*)-*Z*-**M1**	*M*-Z_s	2.0	2.0	2.0	2.0	2.0	2.0	2.0
(2*S*,3*S*)-(*P*,*P*)-*E*-**M1**	*P*-E_{ms}	12.4	12.4	12.4	12.4	12.4	12.4	12.4
(2*S*,3*S*)-(*P*,*M*)-E-**M1**	*P*-E_s	0.0	0.0	0.0	0.0	0.0	0.0	0.0
(2*S*,3*S*)-(*P*,*P*)-Z-**M1**	*P*-Z_{ms}	12.0	12.0	12.0	12.0	12.0	12.0	12.0
(2*S*,3*S*)-(*P*,*M*)-*Z*-**M1**	*P*-Z_s	2.2	2.2	2.2	2.1	2.1	2.1	2.1
M-*E*-TS-THI_{up}		104.5	104.5	104.5	104.4	104.4	104.4	104.4
M-*Z*-TS-THI_{up}		115.1	115.2	115.2	115.7	115.8	115.9	115.9
P-*E*-TS-THI_{up}		115.0	115.1	115.2	115.8	115.9	116.0	116.1
P-*Z*-TS-THI_{up}		109.9	110.0	110.0	110.6	110.8	110.9	110.9
E_{ms}-TS-THI_{low}		84.2	84.3	84.3	84.7	84.8	84.9	84.9
E_s-TS-THI_{low}		76.6	76.7	76.7	77.1	77.2	77.3	77.3
Z_{ms}-TS-THI_{low}		84.2	84.2	84.3	84.6	84.7	84.8	84.8
Z_s-TS-THI_{low}		73.7	73.8	73.8	74.3	74.4	74.4	74.5

7. HPLC chromatograms

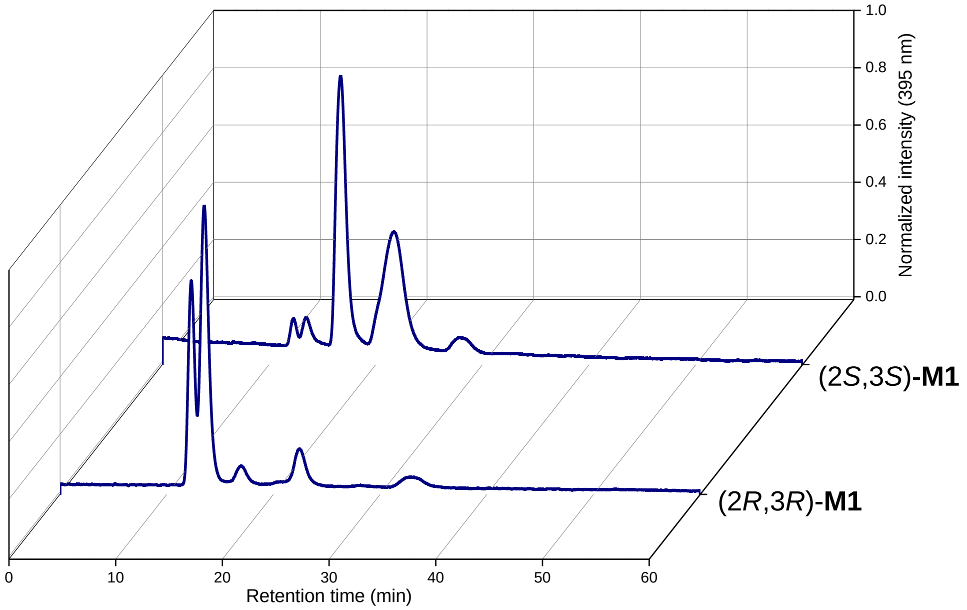

Figure S34. Stacked HPLC Chromatogram of (2*R*,3*R*)-**M1** and (2*S*,3*S*)-**M1** (Chiralcel OD-H, *n*-heptane/2-propanol 99.5:0.5, 1.0 mL/min, 80 °C).

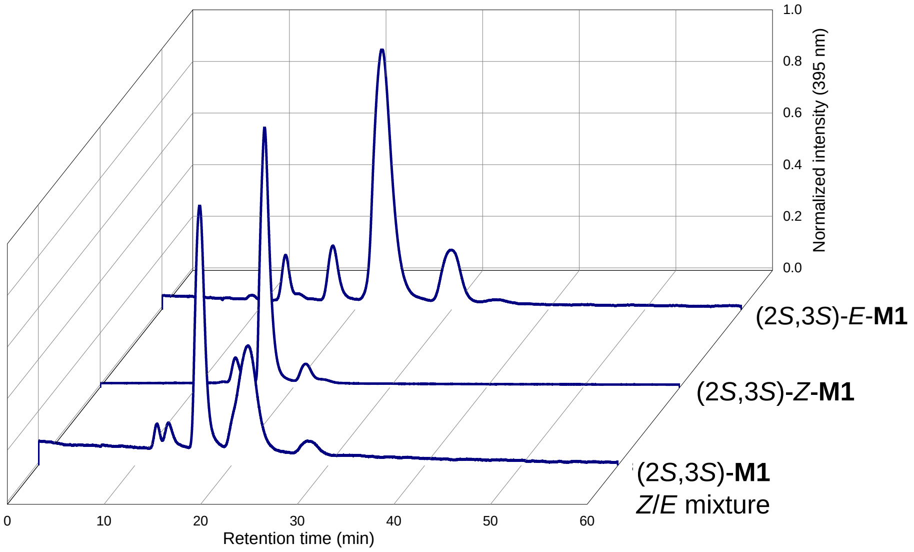

Figure S35. Stacked HPLC Chromatogram of (2*S*,3*S*)-**M1** as a mixture of *Z* and *E* isomers, and separated (2*S*,3*S*)-Z-**M1** and (2*S*,3*S*)-*E*-**M1** (Chiralcel OD-H, *n*-heptane/2-propanol 99.5:0.5, 1.0 mL/min, 80 °C).

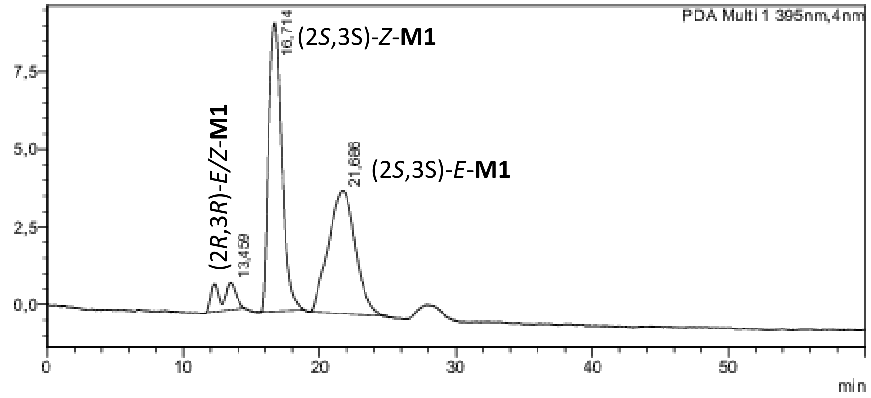

<Peak Table>

PDA Ch1 395nm

Peak#	Ret. Time	Area	Area%
1	13,459	78175	6,273
2	16,714	621390	49,861
3	21,686	546692	43,867
Tota		1246257	100,000

Figure S36. HPLC Chromatogram of (2*S*,3*S*)**-M1** (Chiralcel OD-H, *n*-heptane/2-propanol 99.5:0.5, 1.0 mL/min, 80 °C).

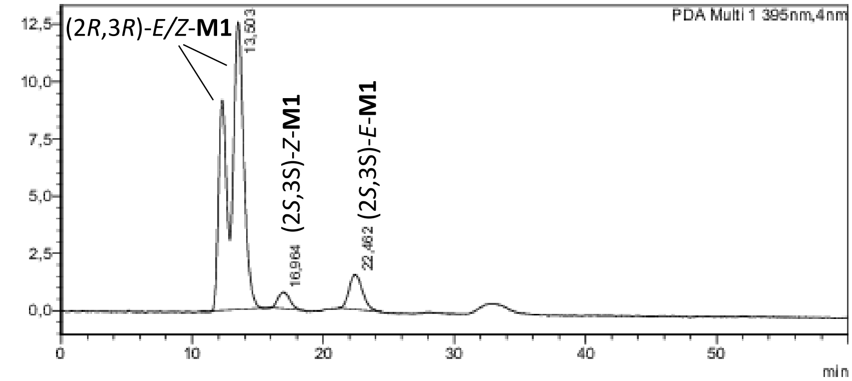

<Peak Table>

PDA Ch1 395nm

Peak#	Ret. Time	Area	Area%
1	13,503	1071936	87,454
2	16,964	45322	3,698
3	22,462	108461	8,849
Tota		1225720	100,000

Figure S37. HPLC Chromatogram of (2*R*,3*R*)**-M1**(Chiralcel OD-H, *n*-heptane/2-propanol 99.5:0.5, 1.0 mL/min, 80 °C).

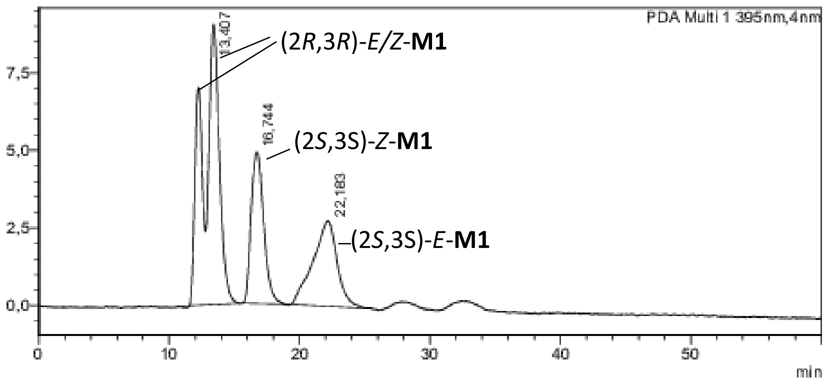

<Peak Table>

PDA Ch1 395nm

Peak#	Ret. Time	Area	Area%
1	13,407	782639	53,452
2	16,744	327777	22,386
3	22,183	353778	24,162
Tota		1464193	100,000

Figure S38. HPLC Chromatogram of a mixture of (2*S*,3*S*)**-M1** and (2*R*,3*R*)**-M1** (Chiralcel OD-H, *n*-heptane/2-propanol 99.5:0.5, 1.0 mL/min, 80 °C).

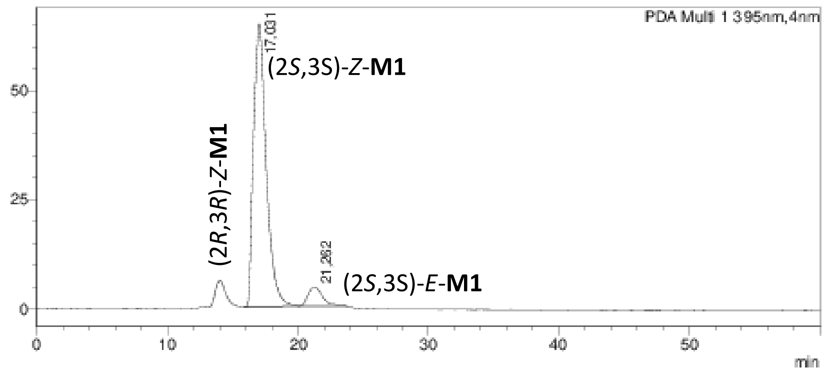

<Peak Table>

PDA Ch1 395nm

Peak#	Ret. Time	Area	Area%
1	17,031	4330688	92,674
2	21,262	342371	7,326
Total		4673059	100,000

Figure S39. HPLC Chromatogram of (2*S*,3*S*)-*Z*-**M1** (Chiralcel OD-H, *n*-heptane/2-propanol 99.5:0.5, 1.0 mL/min, 80 °C).

8. NMR spectra of new compounds

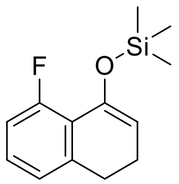

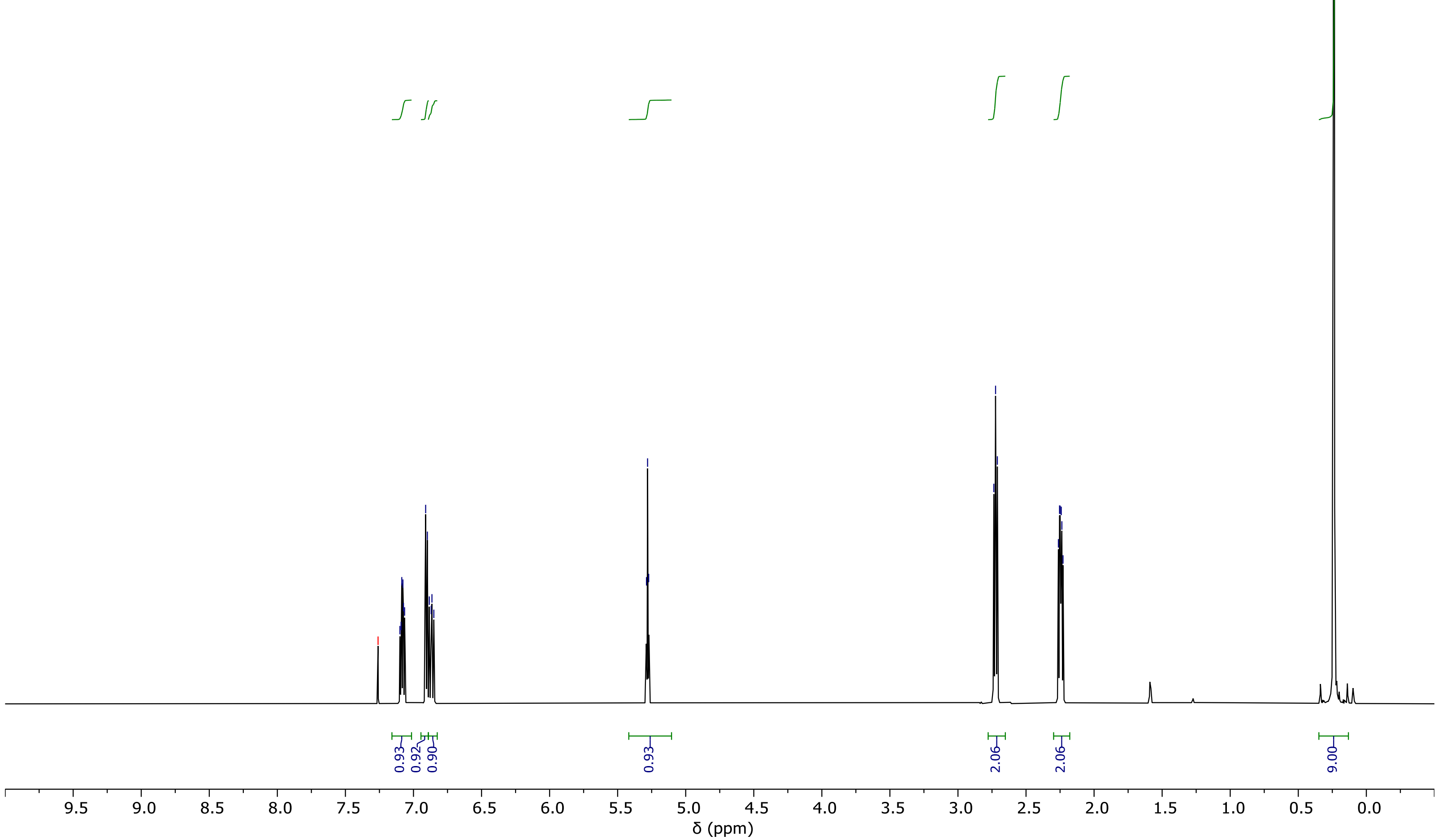

^{1}H-NMR spectrum of compound **1** (600 MHz, $CDCl_3$, 25 °C).

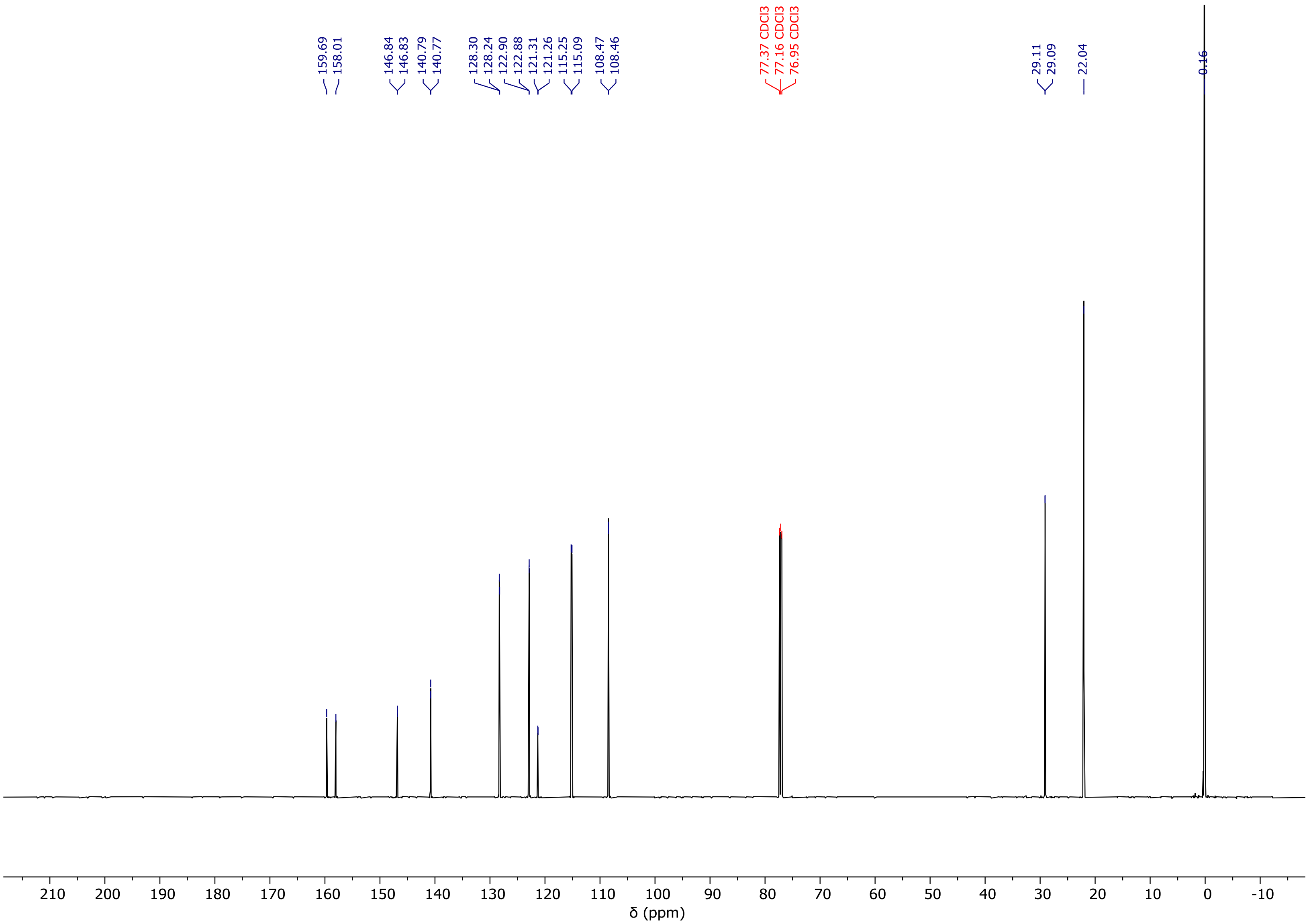

$^{13}C\{^{1}H\}$-NMR spectrum of compound **1** (151 MHz, $CDCl_3$, 25 °C).

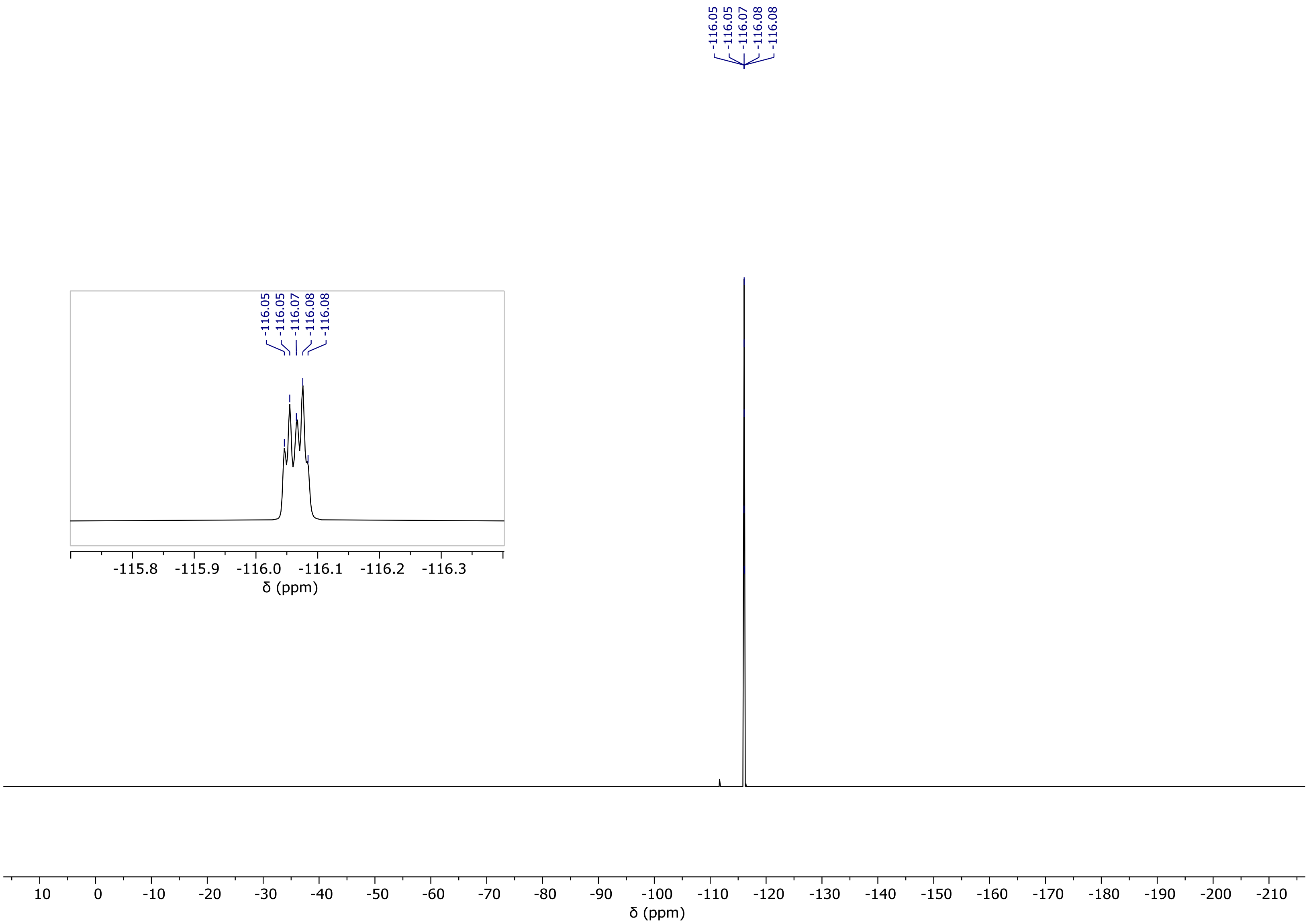

^{19}F NMR spectrum of compound **1** (565 MHz, $CDCl_3$, 25 °C).

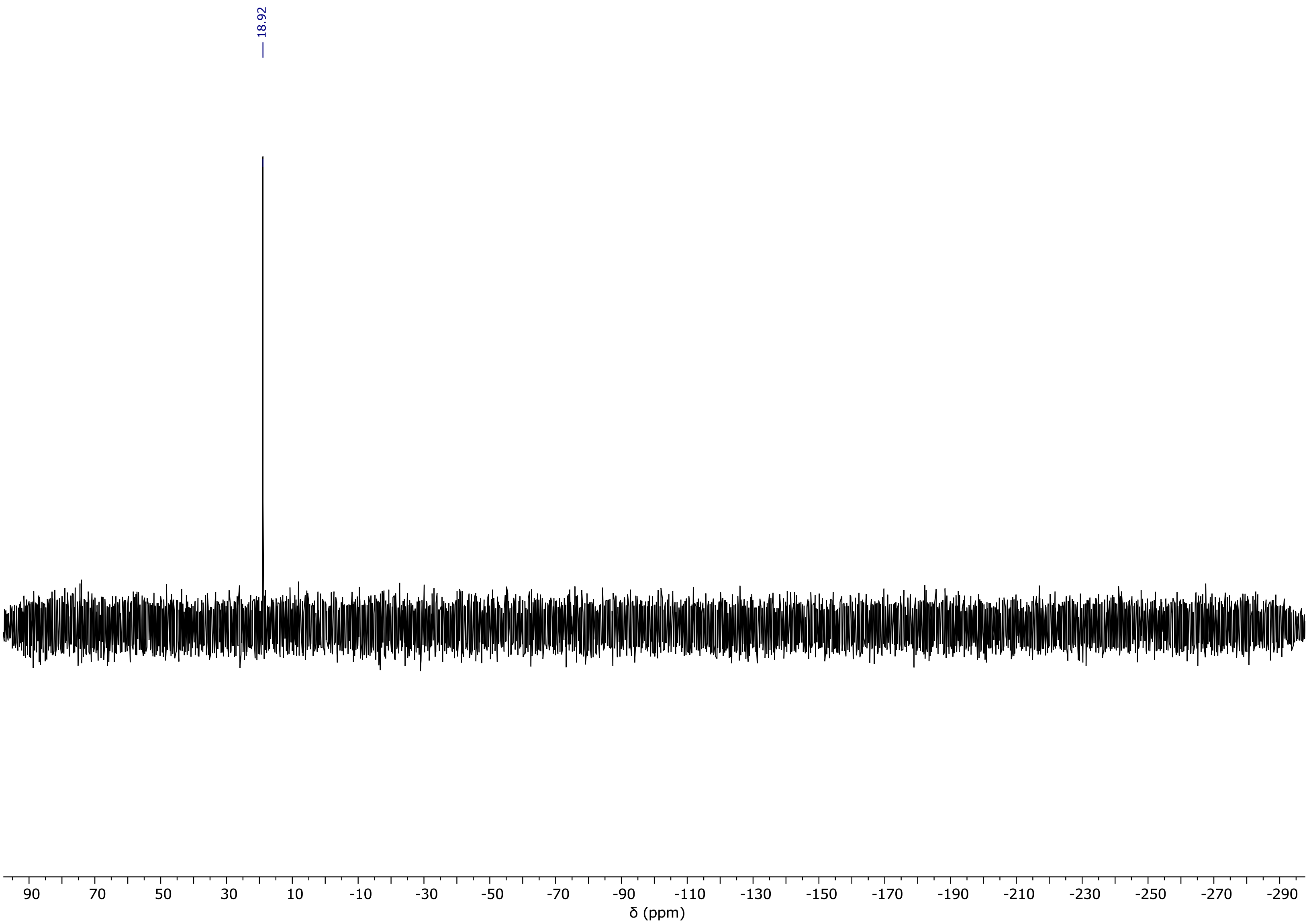

$^{29}Si\{^1H\}$-NMR spectrum of compound **1** (119 MHz, $CDCl_3$, 25 °C).

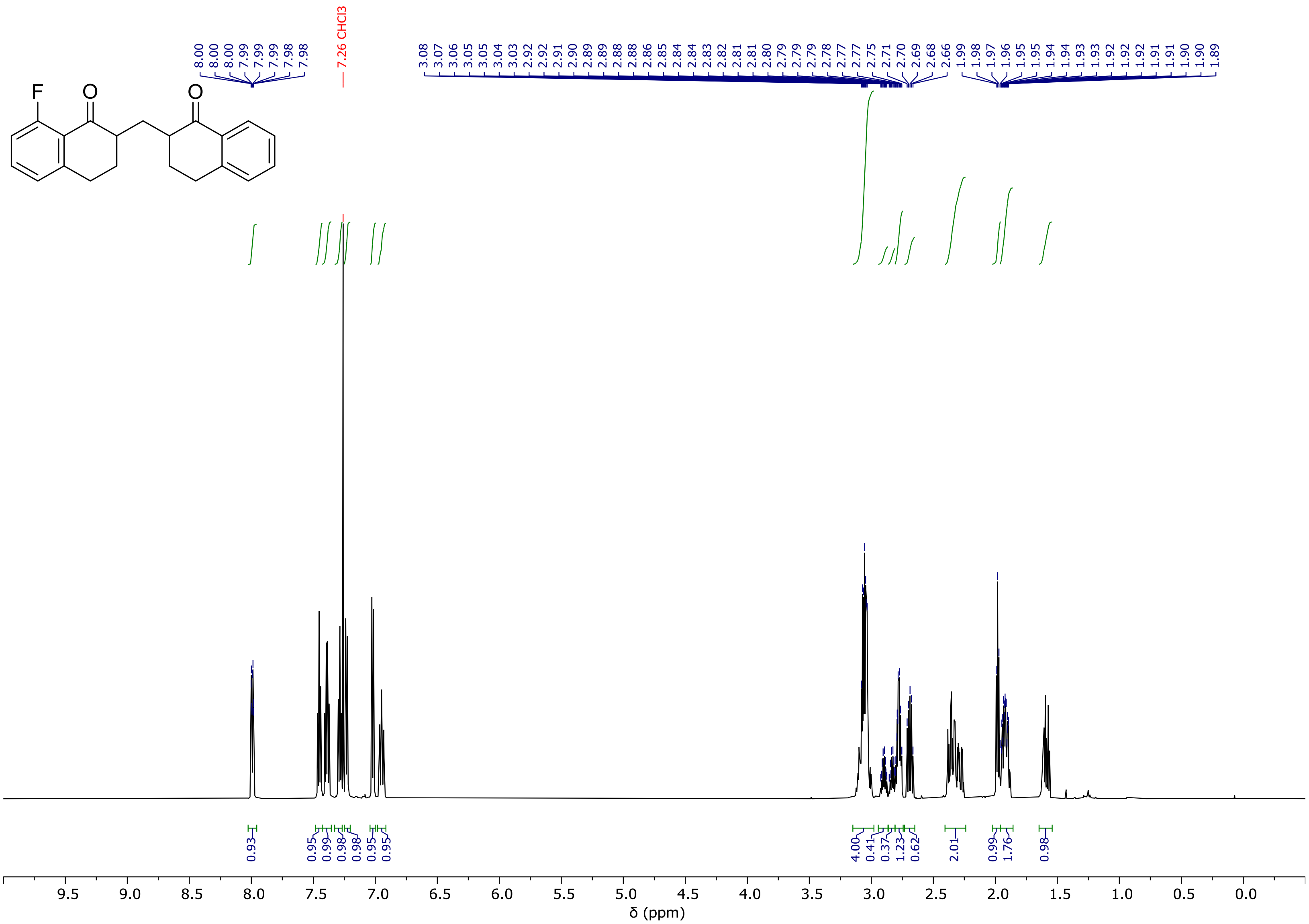

^{1}H-NMR spectrum of compound **2** (600 MHz, $CDCl_3$, 25 °C).

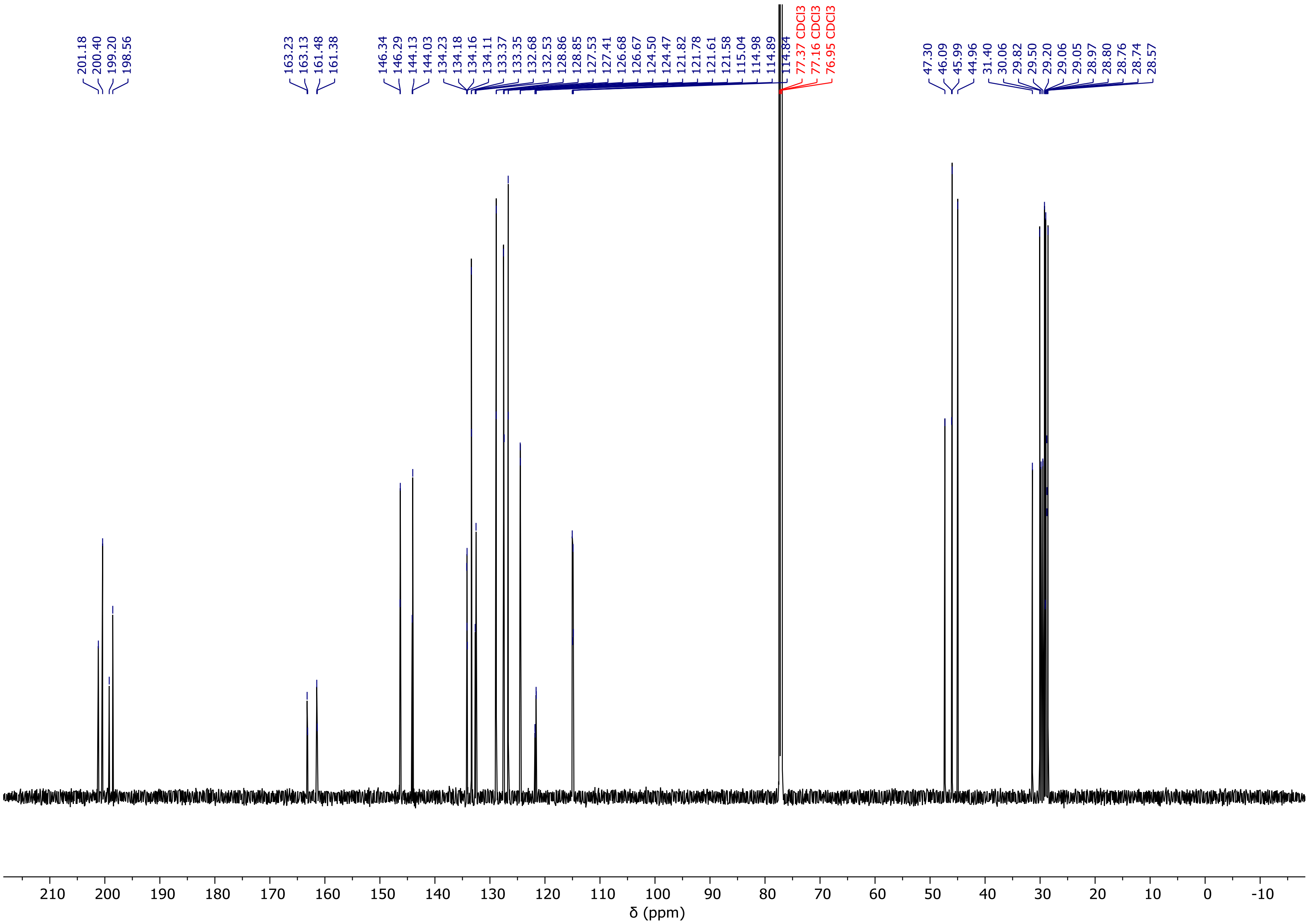

$^{13}C\{^{1}H\}$-NMR spectrum of compound **2** (151 MHz, $CDCl_3$, 25 °C).

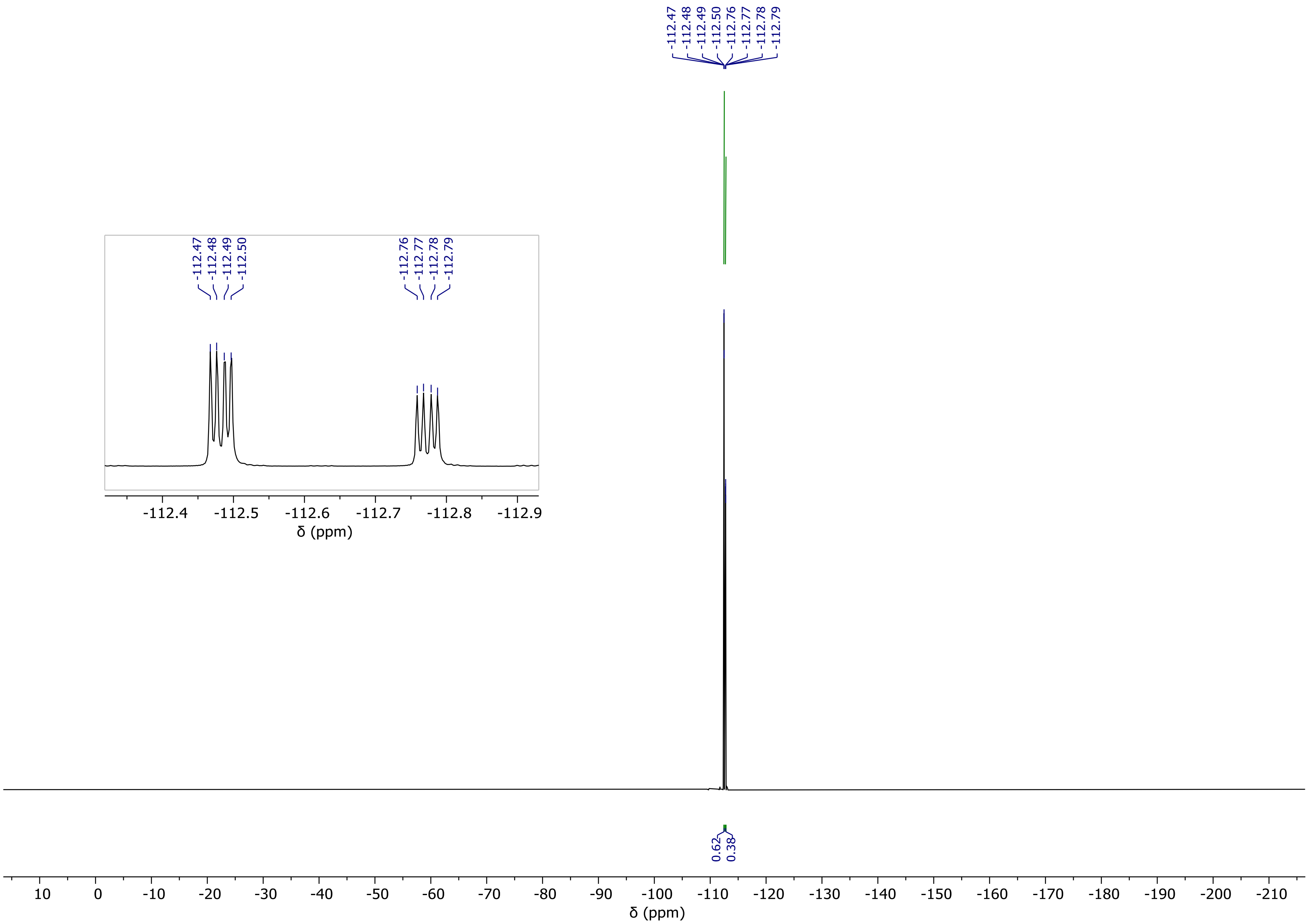

^{19}F-NMR spectrum of compound **2** (565 MHz, $CDCl_3$, 25 °C).

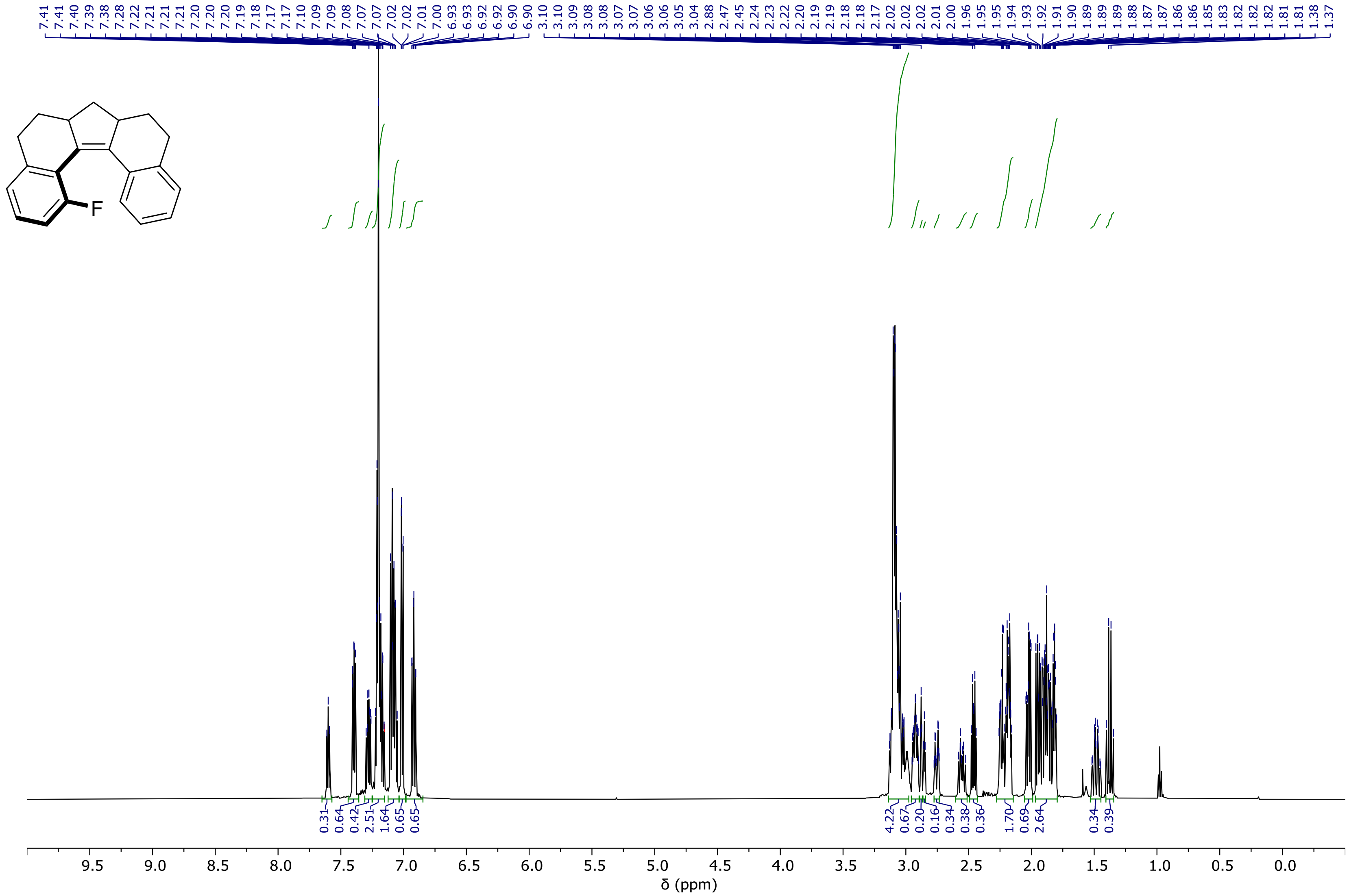

^{1}H-NMR spectrum of compound **3** (600 MHz, $CDCl_3$, 25 °C).

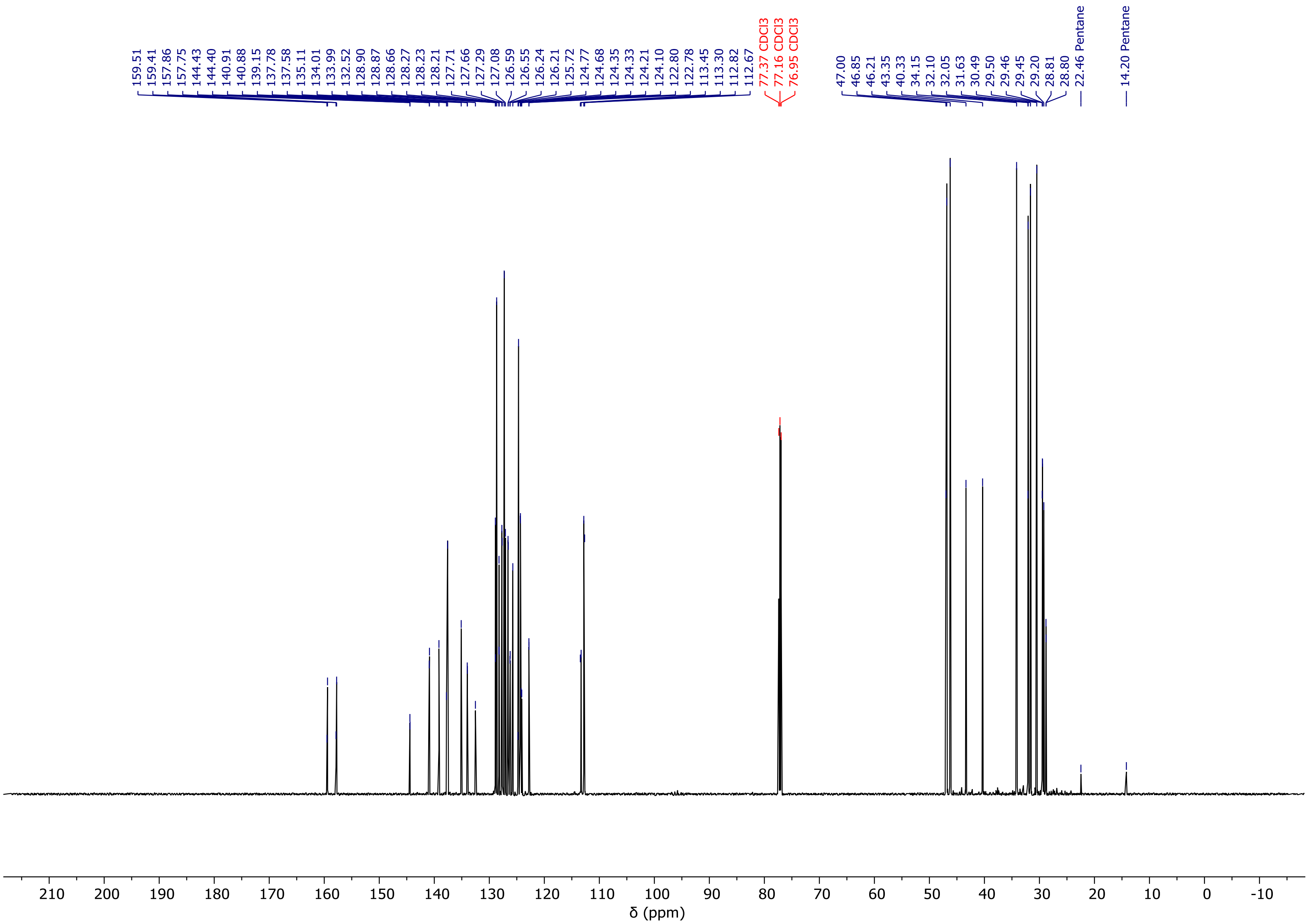

$^{13}C\{^{1}H\}$-NMR spectrum of compound **3** (151 MHz, $CDCl_3$, 25 °C).

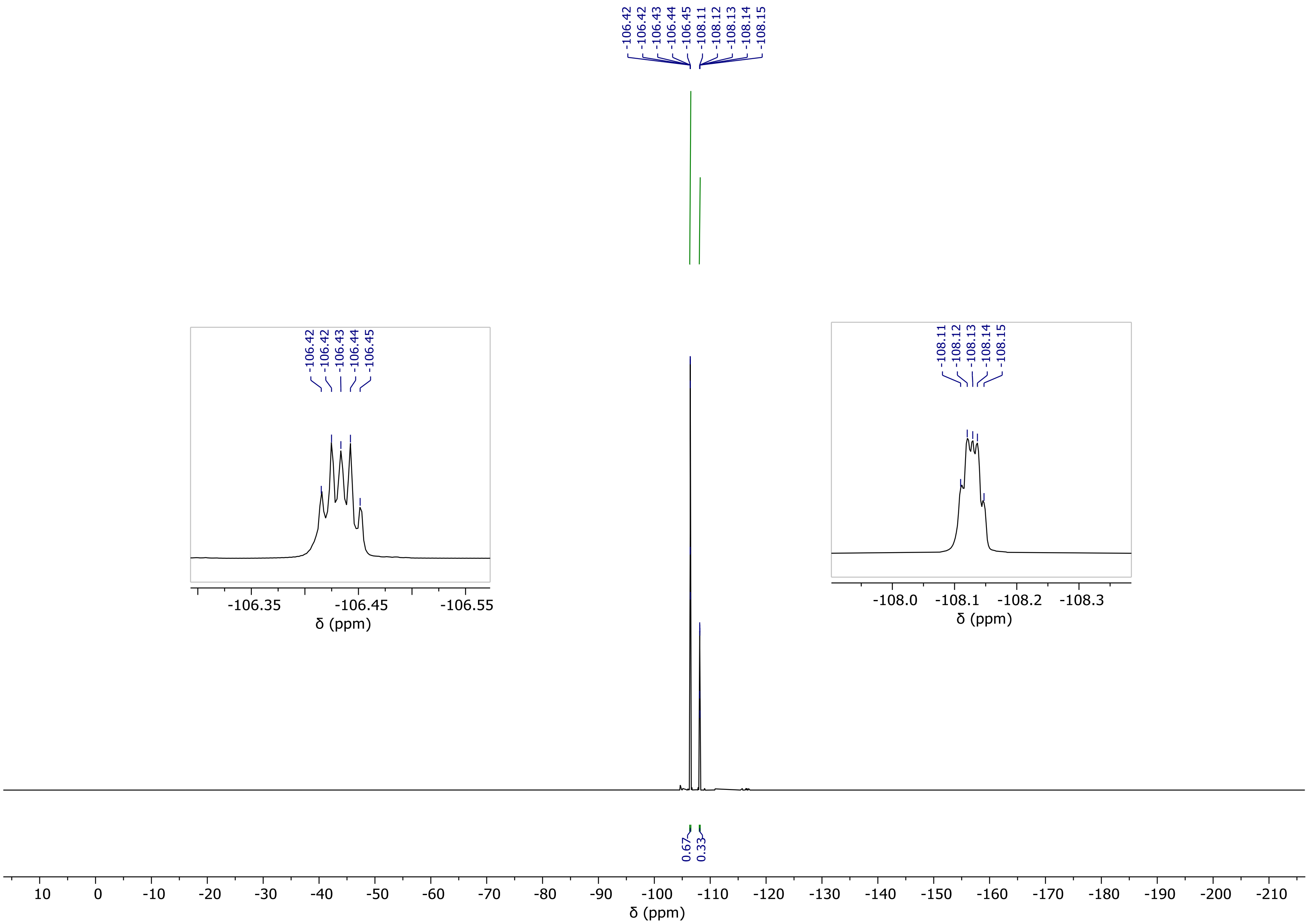

^{19}F-NMR spectrum of compound **3** (565 MHz, $CDCl_3$, 25 °C).

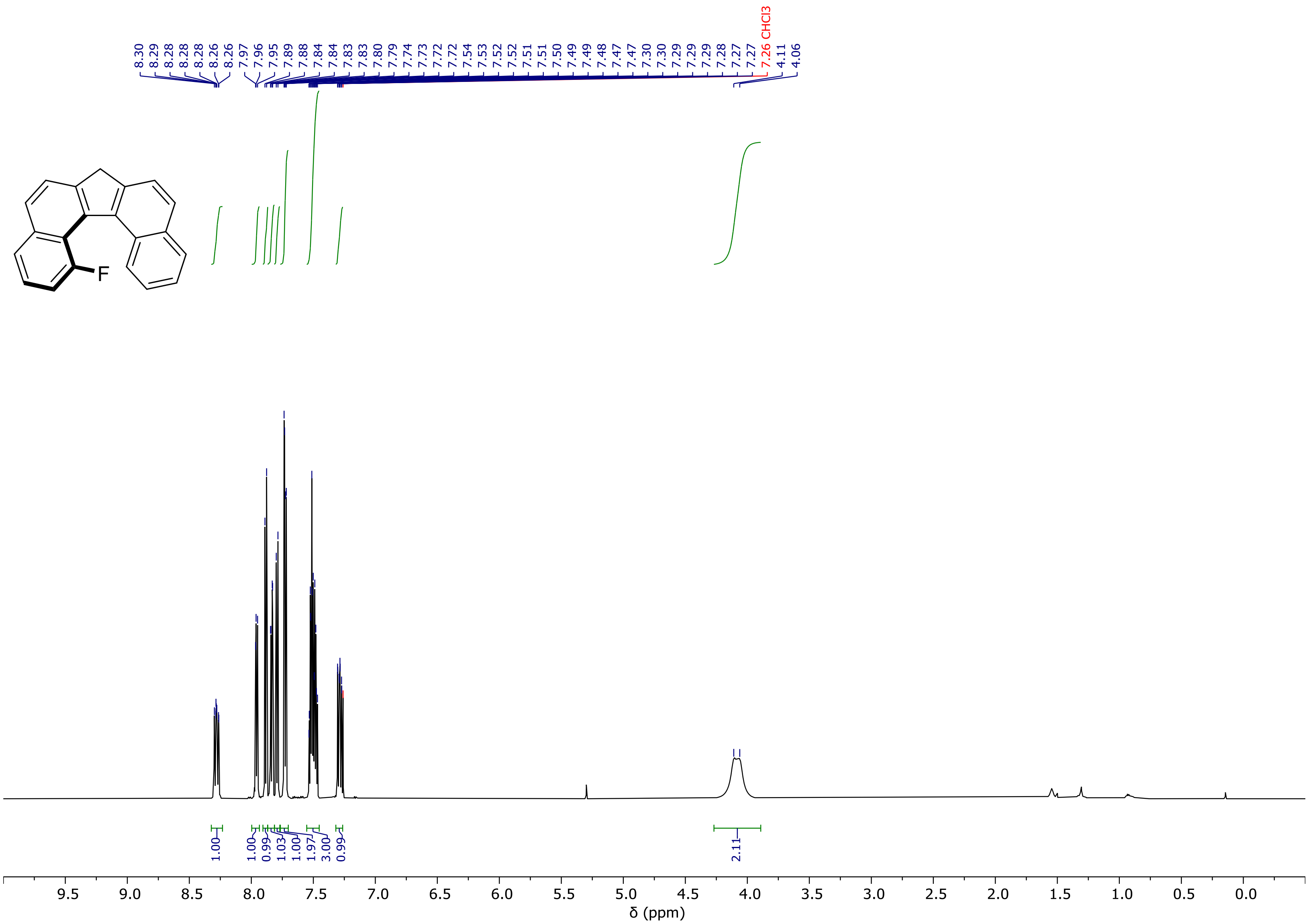

^{1}H-NMR spectrum of compound **4** (600 MHz, $CDCl_3$, 25 °C).

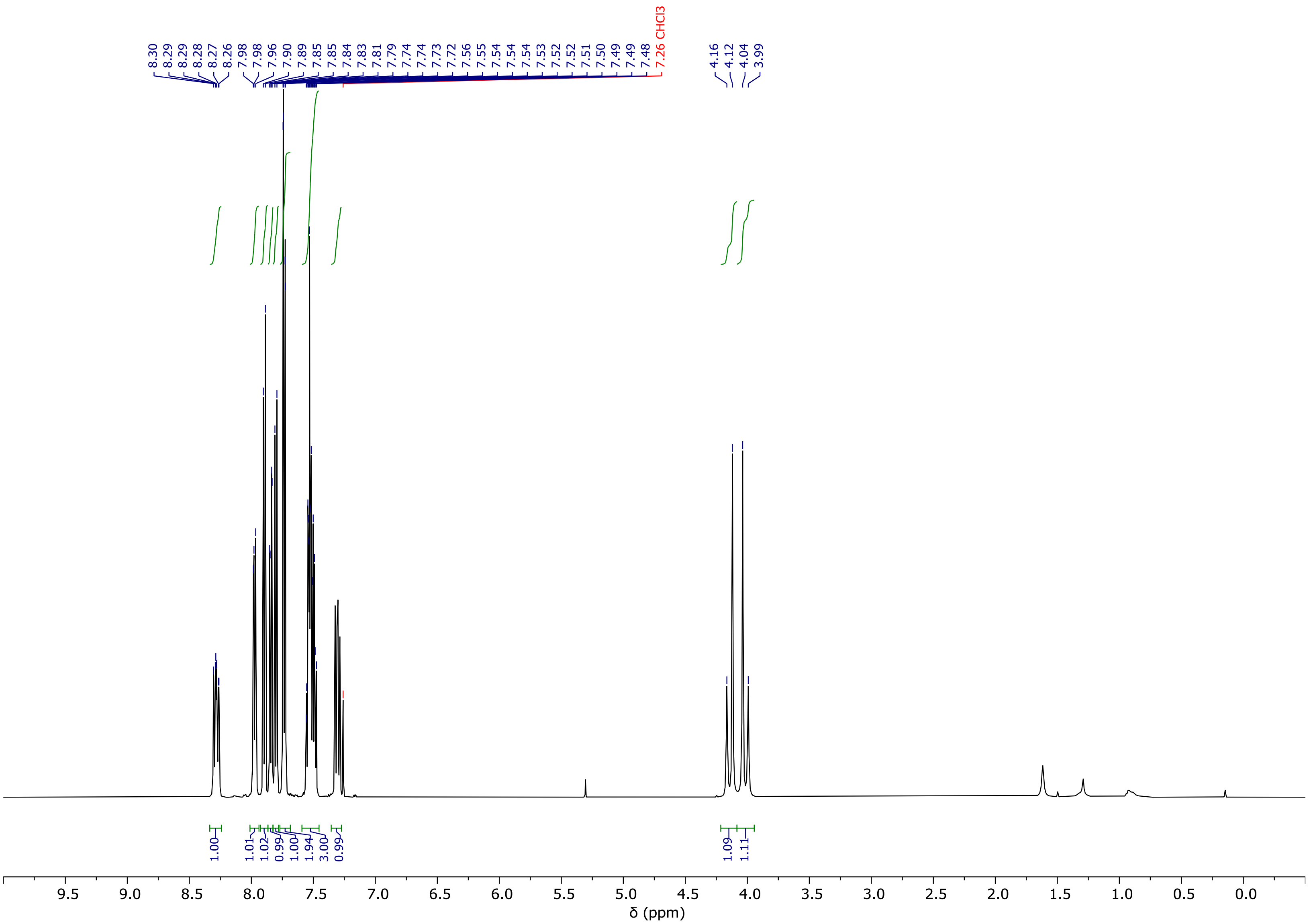

^{1}H-NMR spectrum of compound **4** (500 MHz, $CDCl_3$, –10 °C).

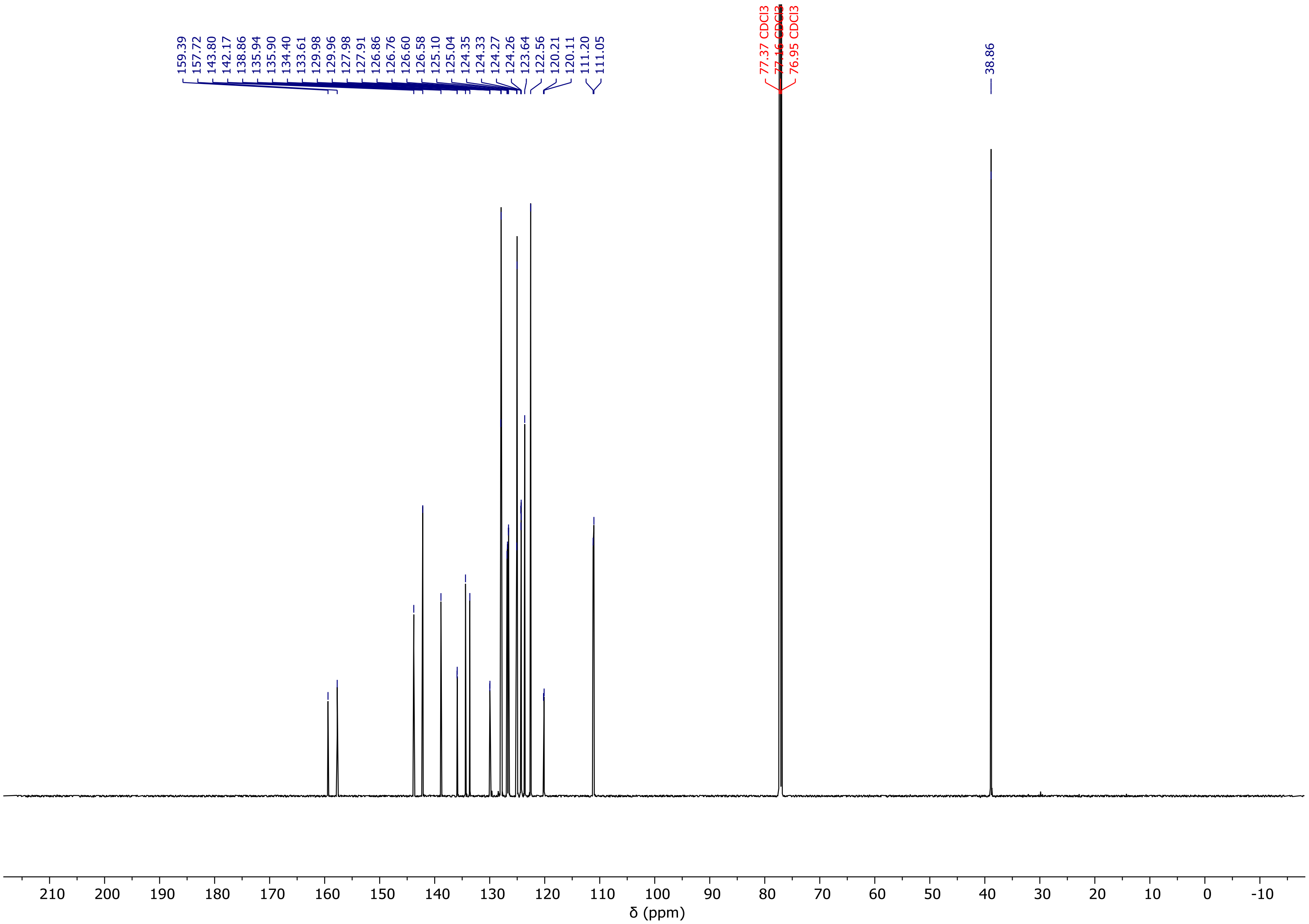

$^{13}C\{^{1}H\}$-NMR spectrum of compound **4** (151 MHz, $CDCl_3$, 25 °C).

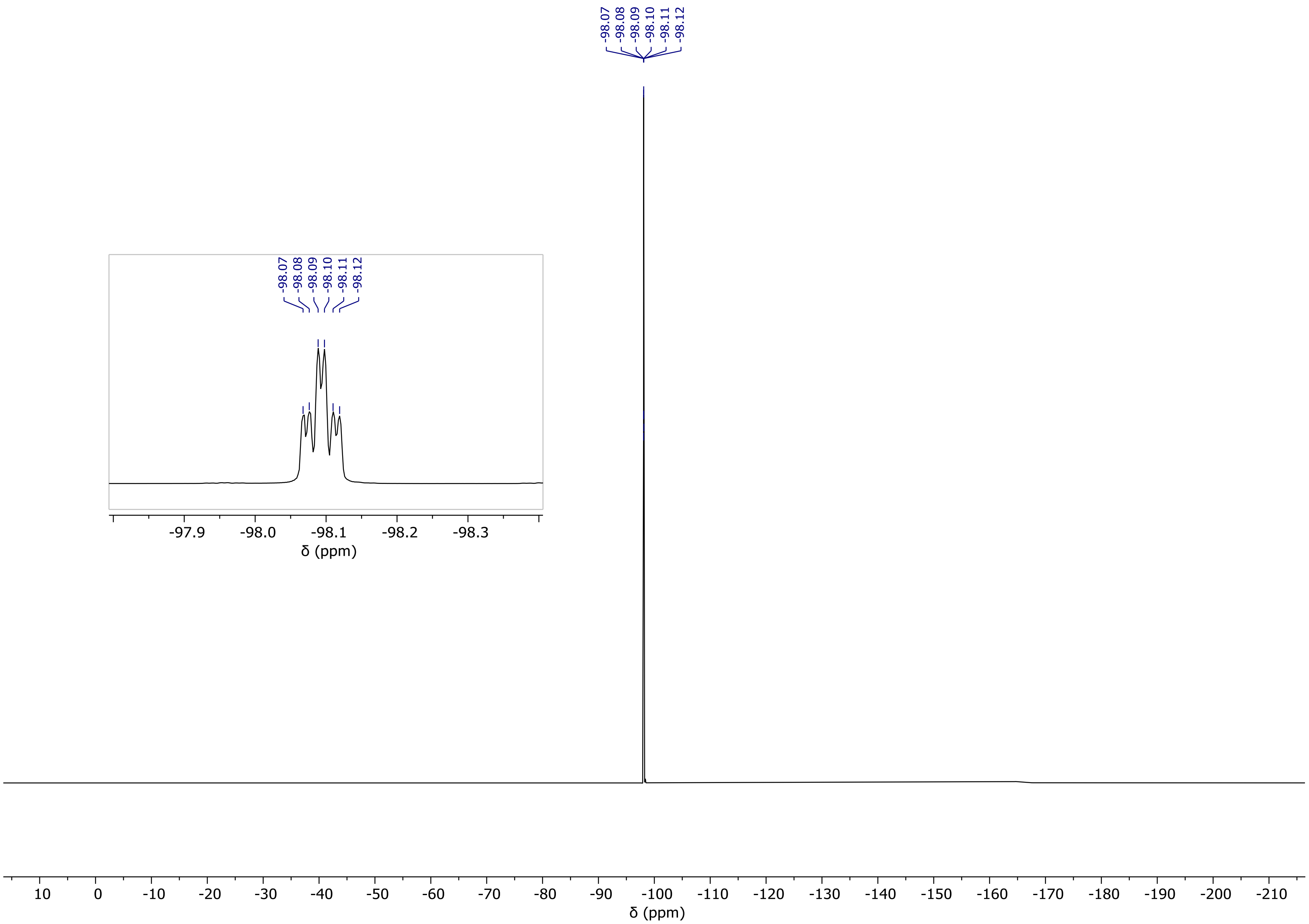

^{19}F-NMR spectrum of compound **4** (565 MHz, $CDCl_3$, 25 °C).

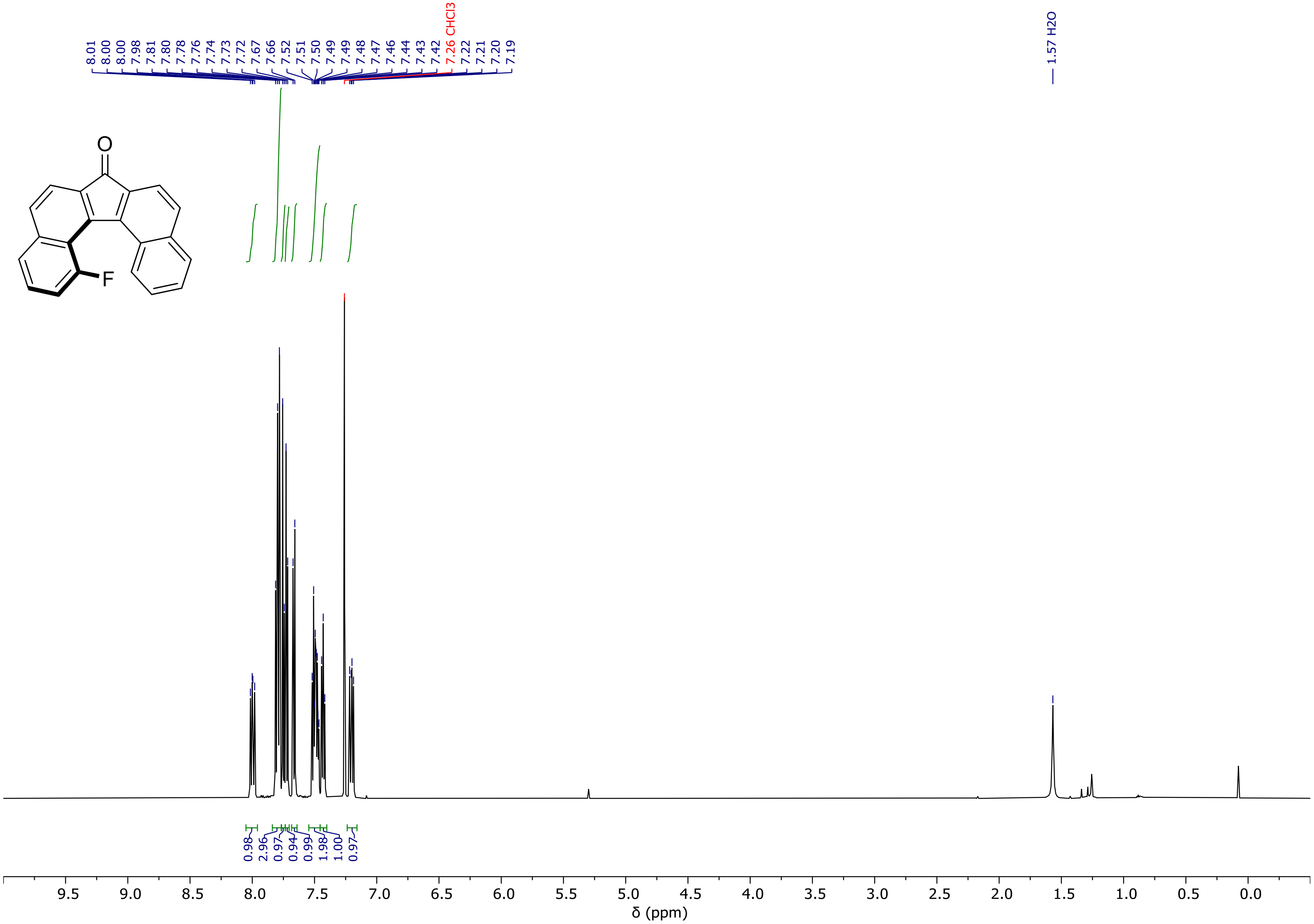

^{1}H-NMR spectrum of compound **5** (600 MHz, $CDCl_3$, 25 °C).

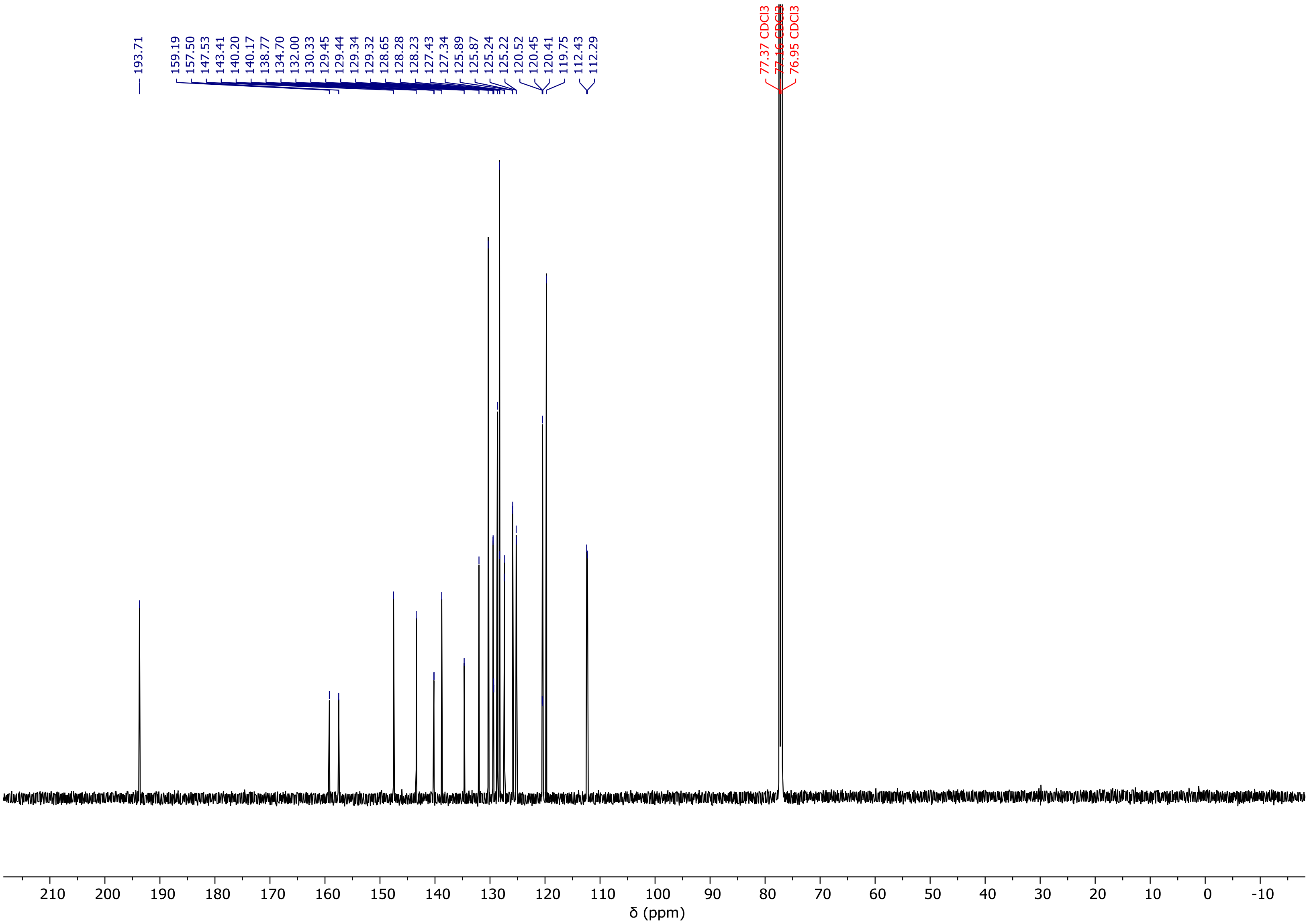

$^{13}C\{^{1}H\}$-NMR spectrum of compound **5** (151 MHz, $CDCl_3$, 25 °C).

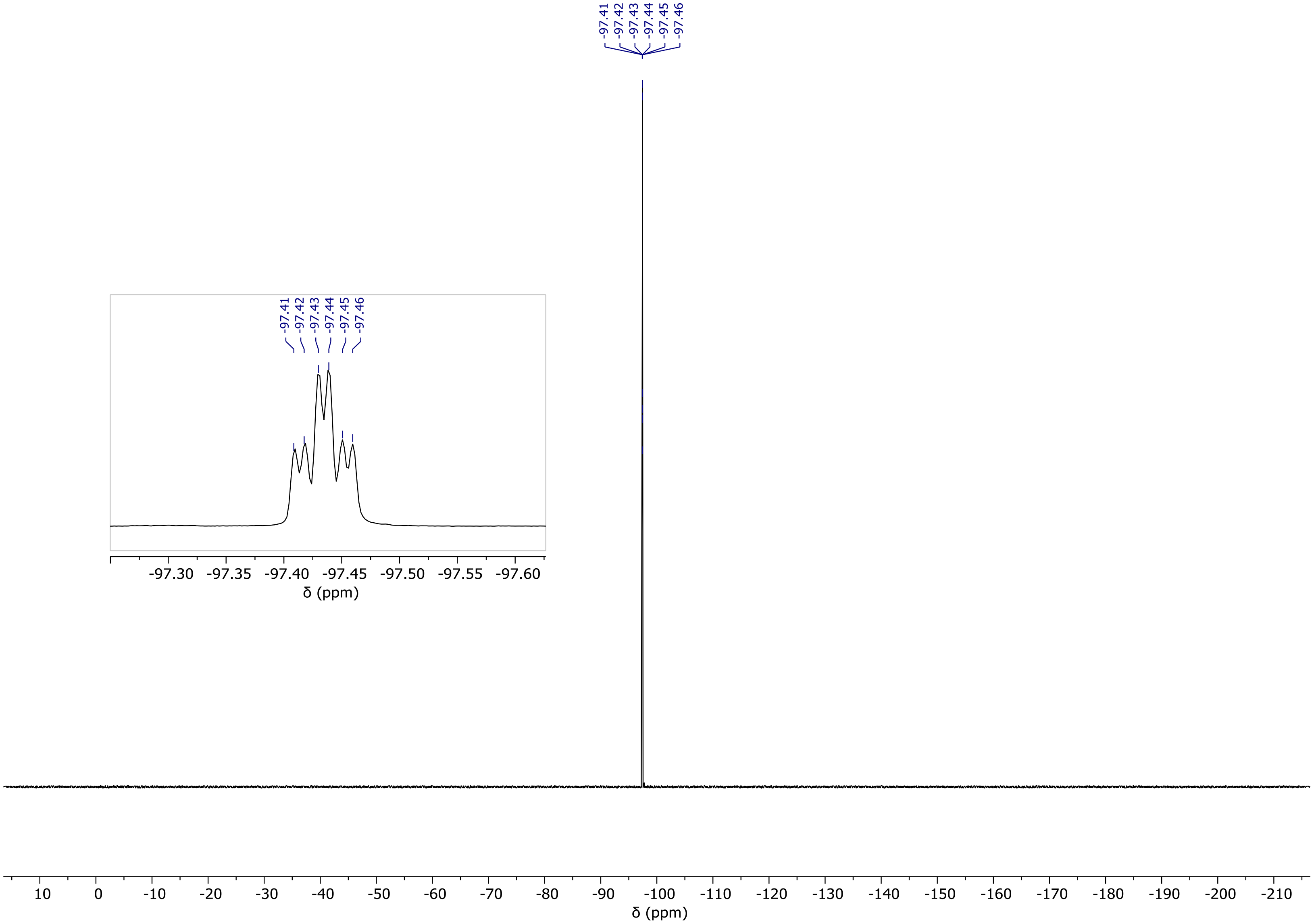

^{19}F-NMR spectrum of compound **5** (565 MHz, $CDCl_3$, 25 °C).

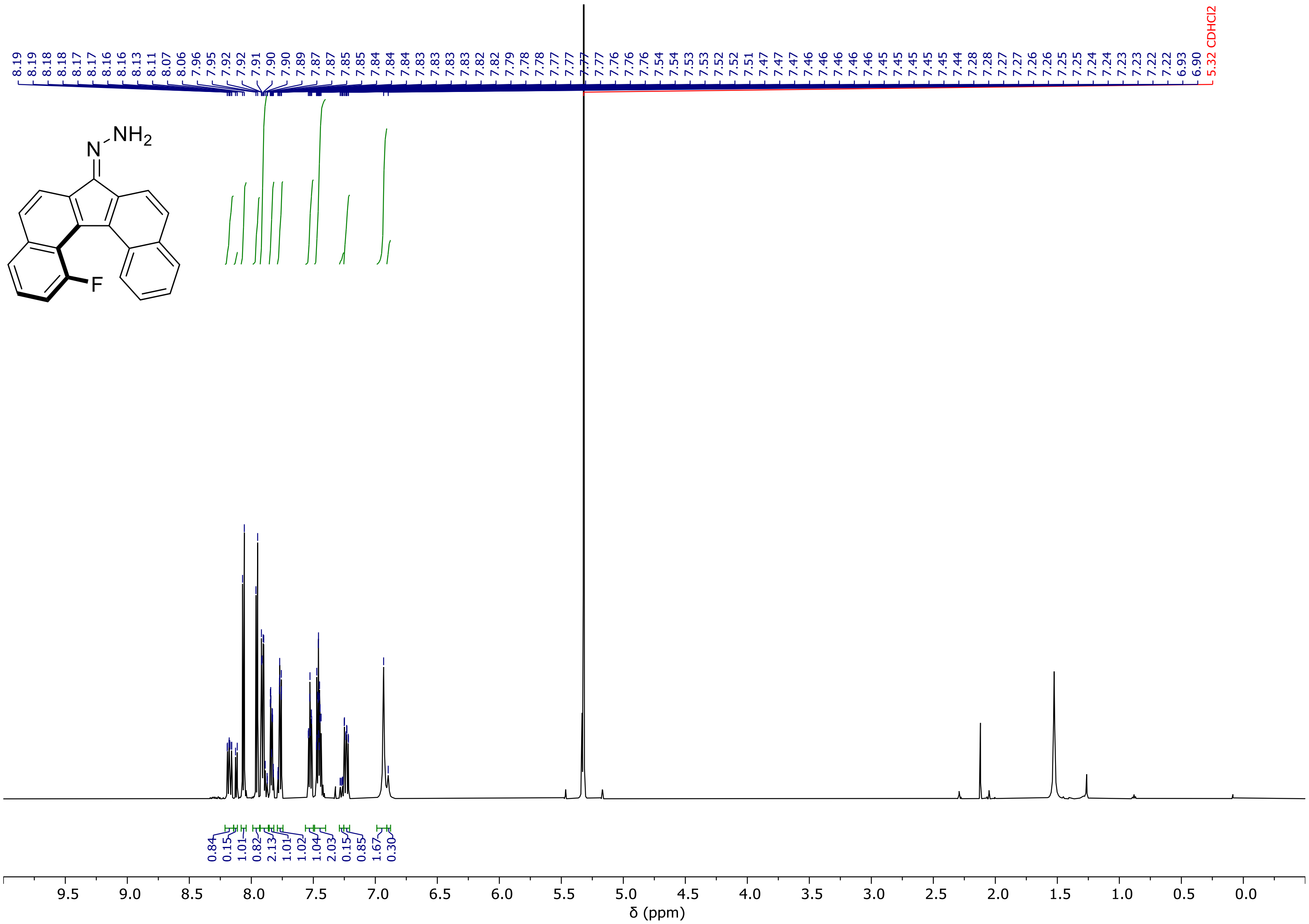

^{1}H-NMR spectrum of compound **6** (600 MHz, CD_2Cl_2, 25 °C).

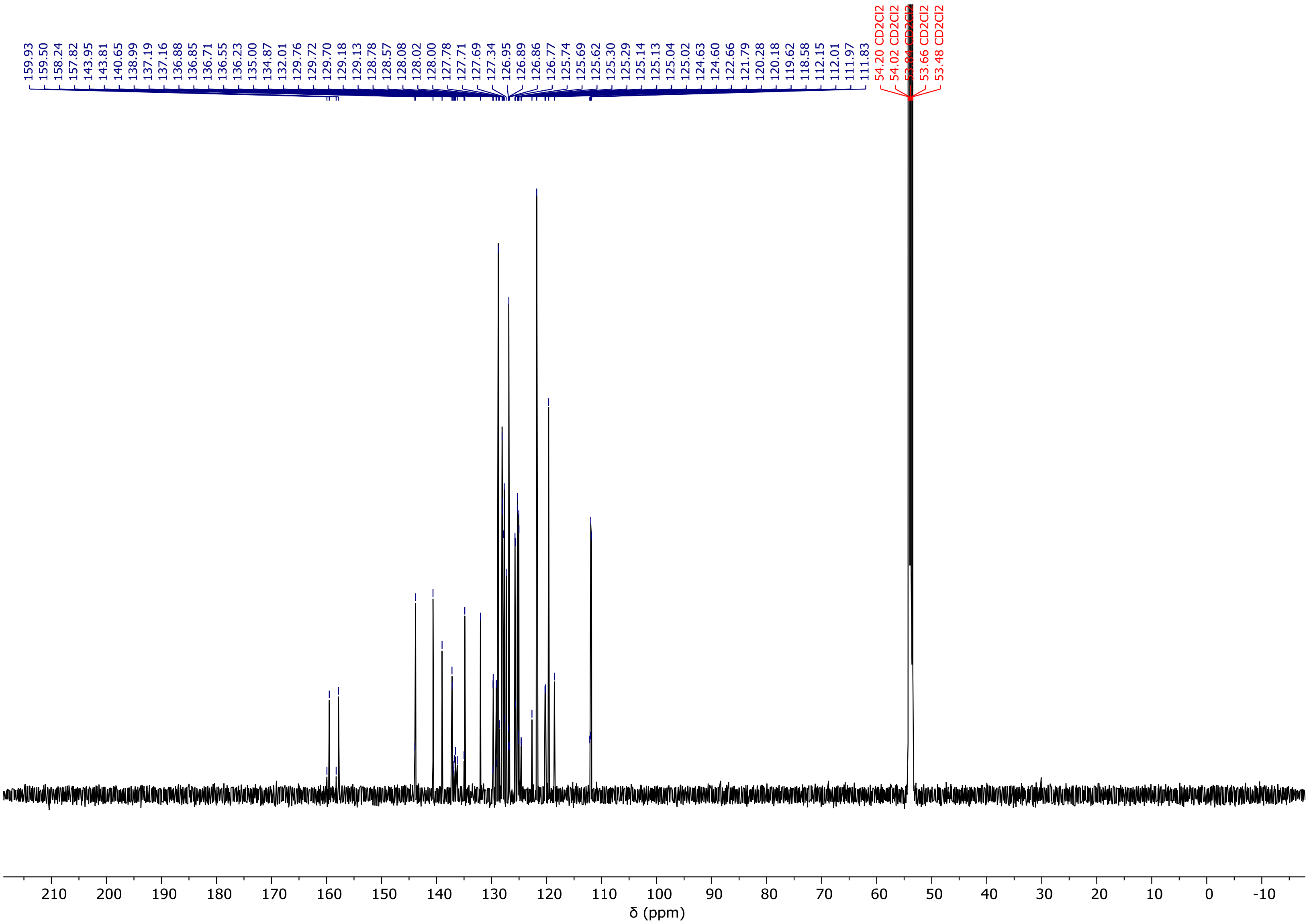

$^{13}C\{^{1}H\}$-NMR spectrum of compound **6** (151 MHz, CD_2Cl_2, 25 °C).

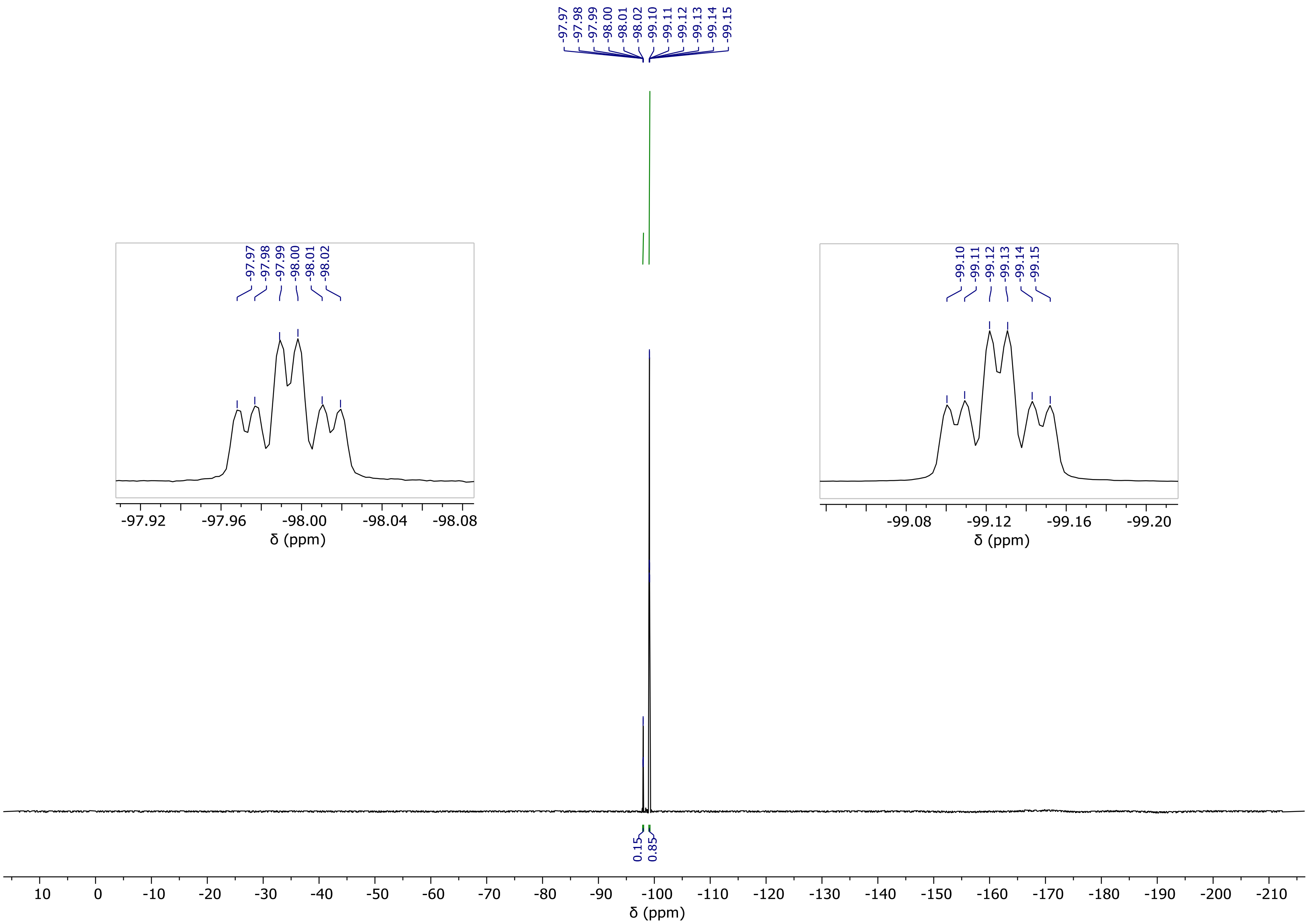

^{19}F-NMR spectrum of compound **6** (565 MHz, CD_2Cl_2, 25 °C).

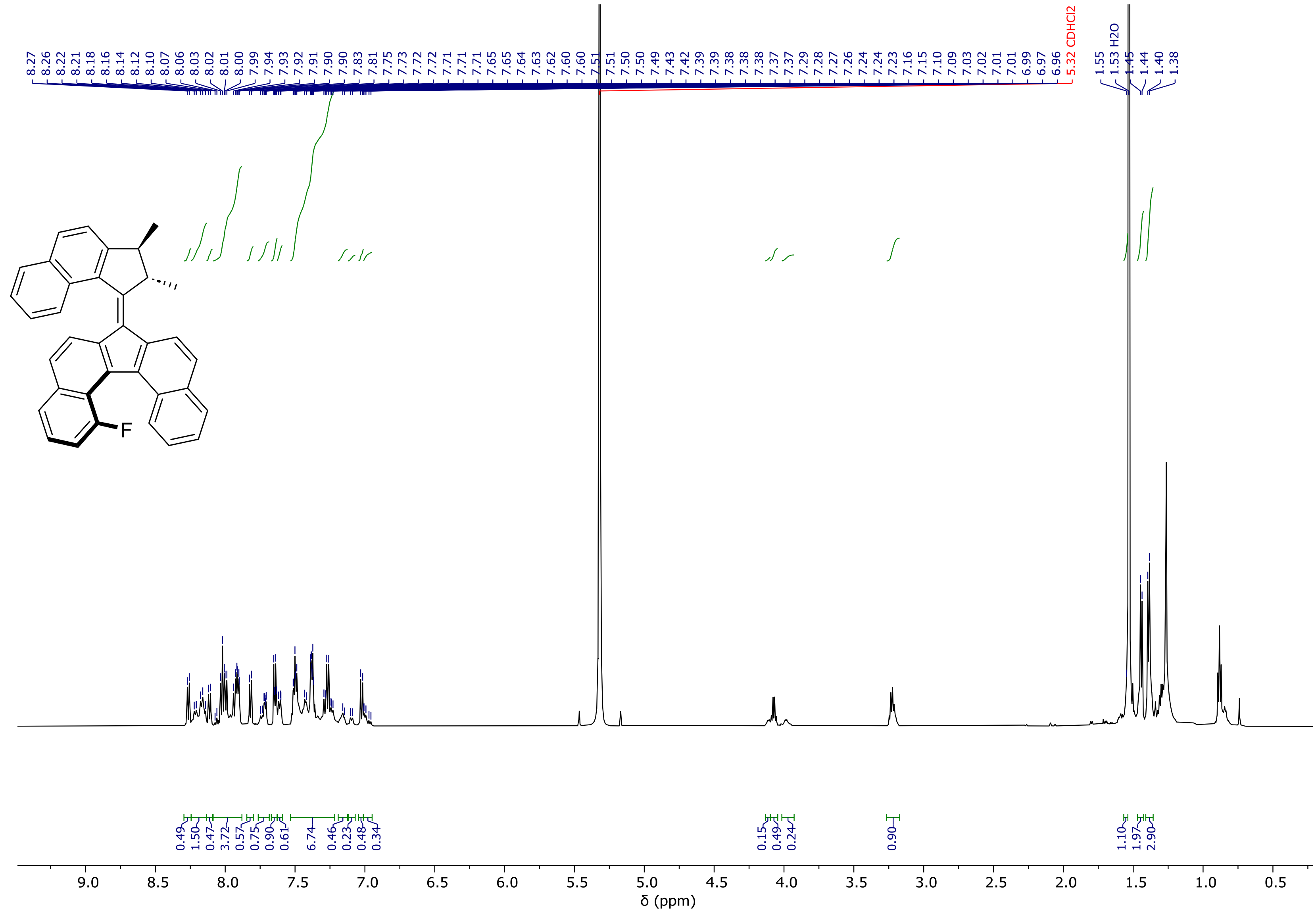

^{1}H-NMR spectrum of compound (2*S*,3*S*)-**M1** (600 MHz, CD_2Cl_2, 25 °C).

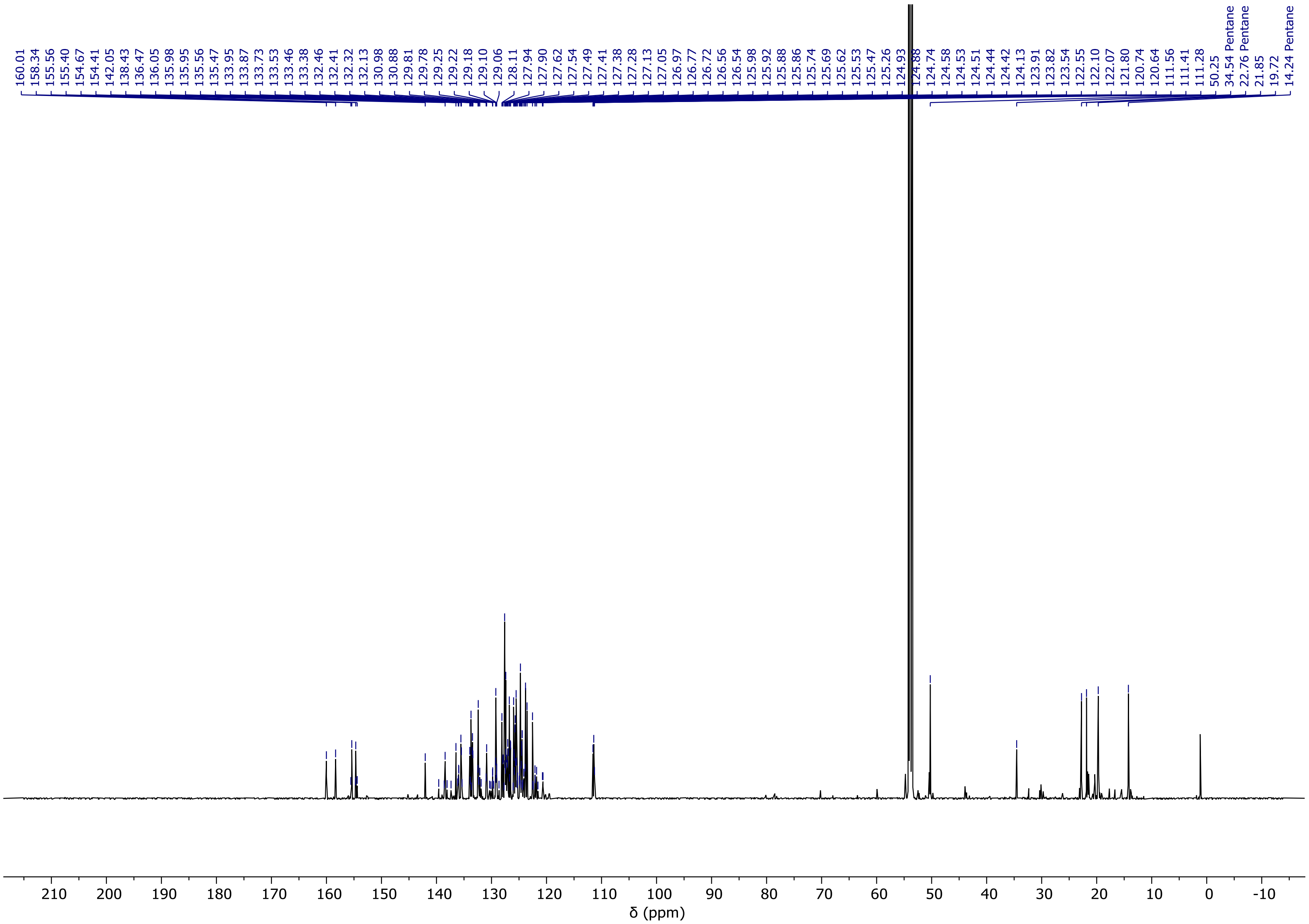

$^{13}C\{^{1}H\}$-NMR spectrum of compound (2*S*,3*S*)-**M1** (151 MHz, CD_2Cl_2, 25 °C).

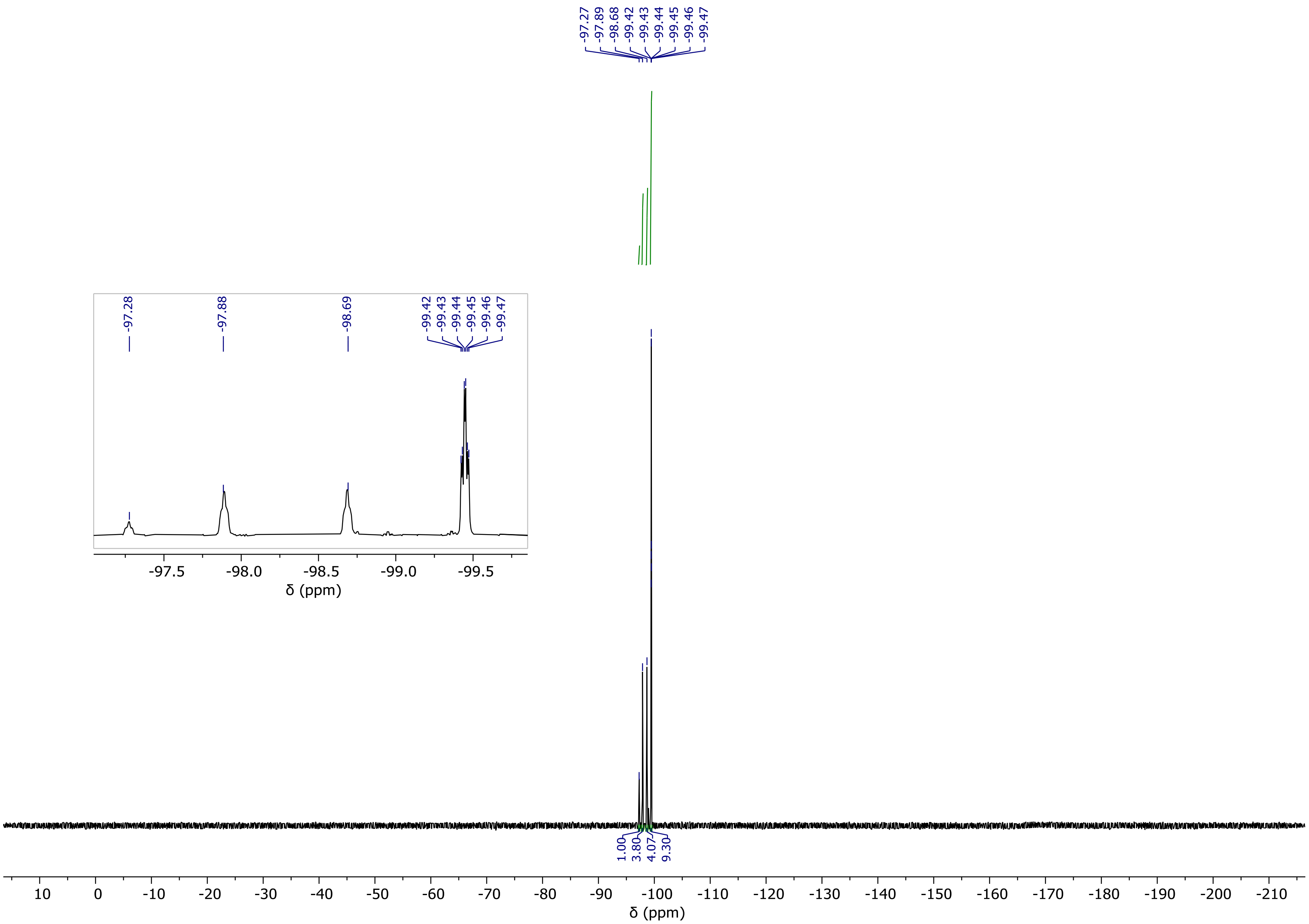

^{19}F-NMR spectrum of compound (2*S*,3*S*)-**M1** (565 MHz, CD_2Cl_2, 25 °C).

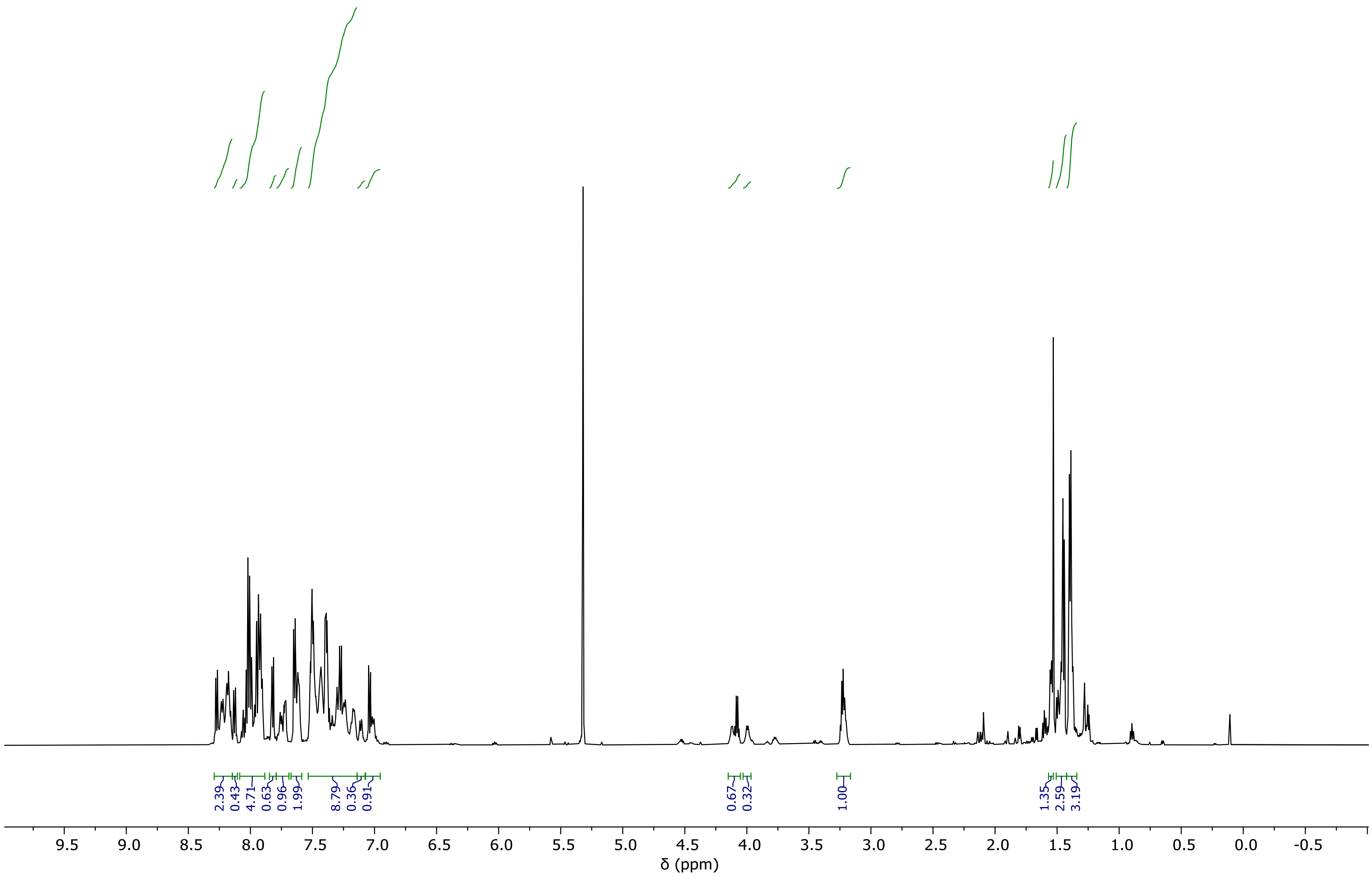
2.39
0.43
4.71
0.63
0.96
1.99
8.79
0.36
0.91
0.67
0.32
1.00
1.35
2.59
3.19
9.5
9.0
8.5
8.0
7.5
7.0
6.5
6.0
5.5
5.0
4.5
4.0
3.5
3.0
2.5
2.0
1.5
1.0
0.5
0.0
-0.5
δ (ppm)

^{1}H-NMR spectrum of compound (2*R*,3*R*)-**M1** (600 MHz, CD_2Cl_2, 25 °C).

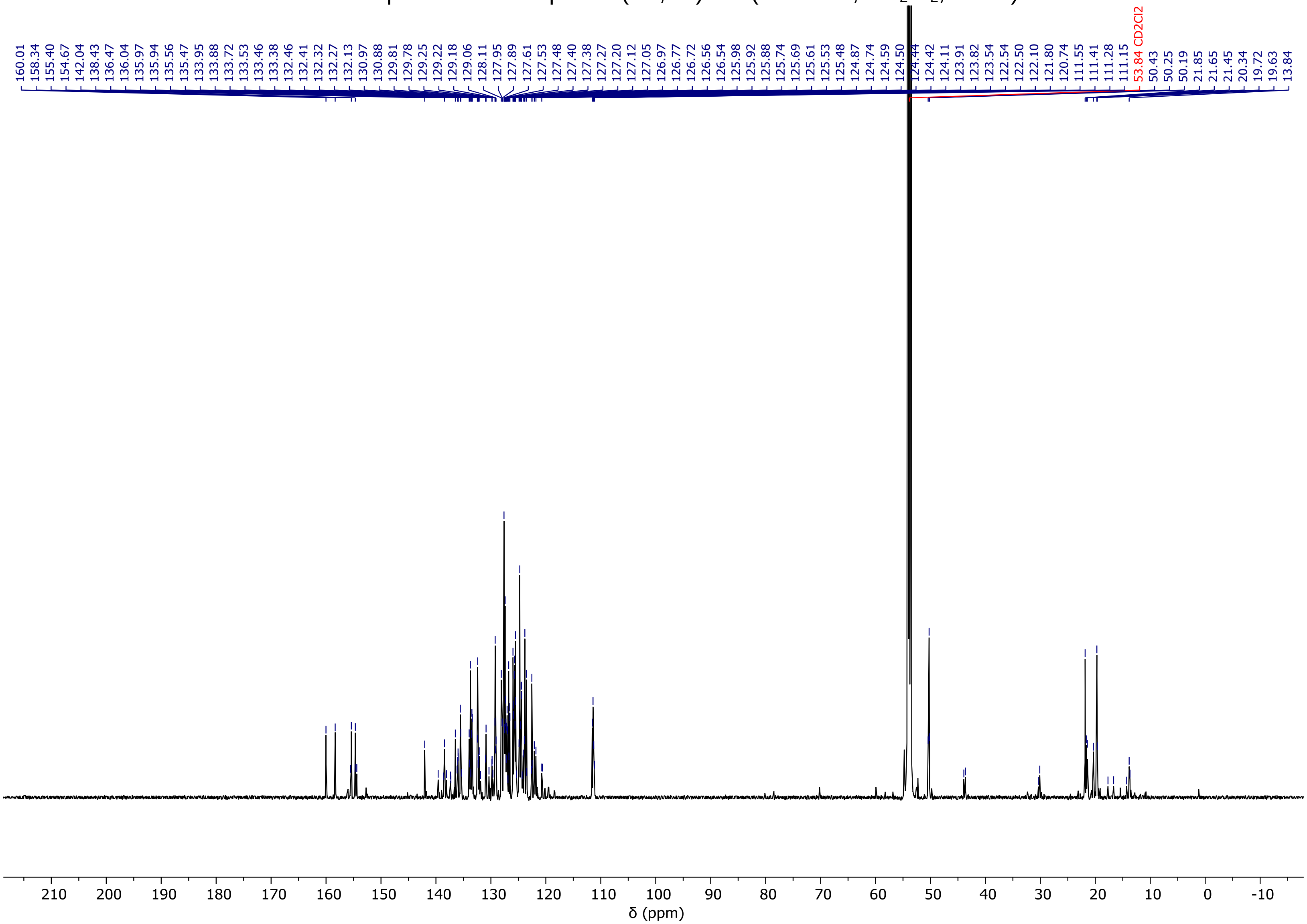

^{13}C{^{1}H}-NMR spectrum of compound (2*R*,3*R*)-**M1** (151 MHz, CD_2Cl_2, 25 °C).

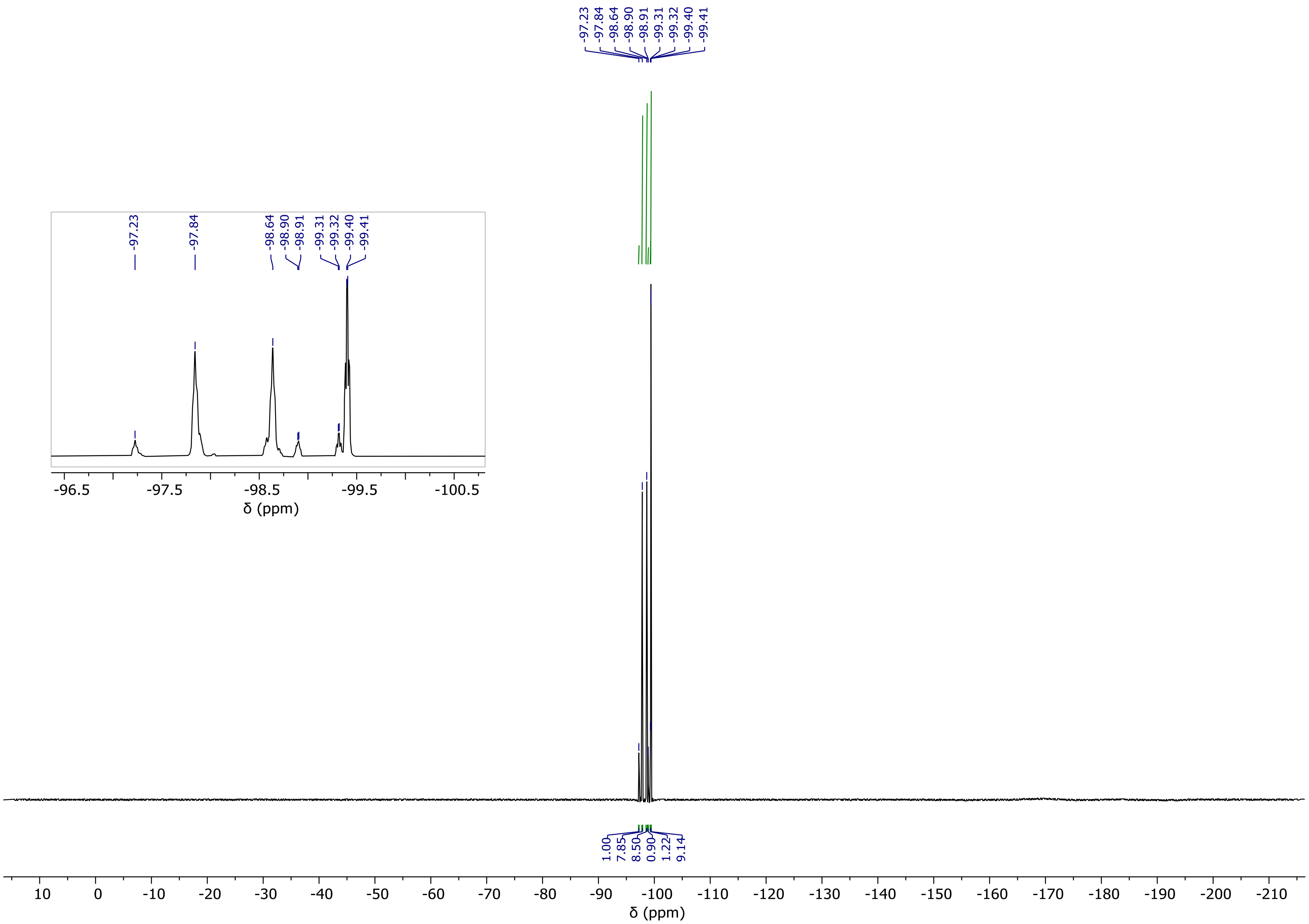

^{19}F-NMR spectrum of compound (2*R*,3*R*)-**M1** (565 MHz, CD_2Cl_2, 25 °C).

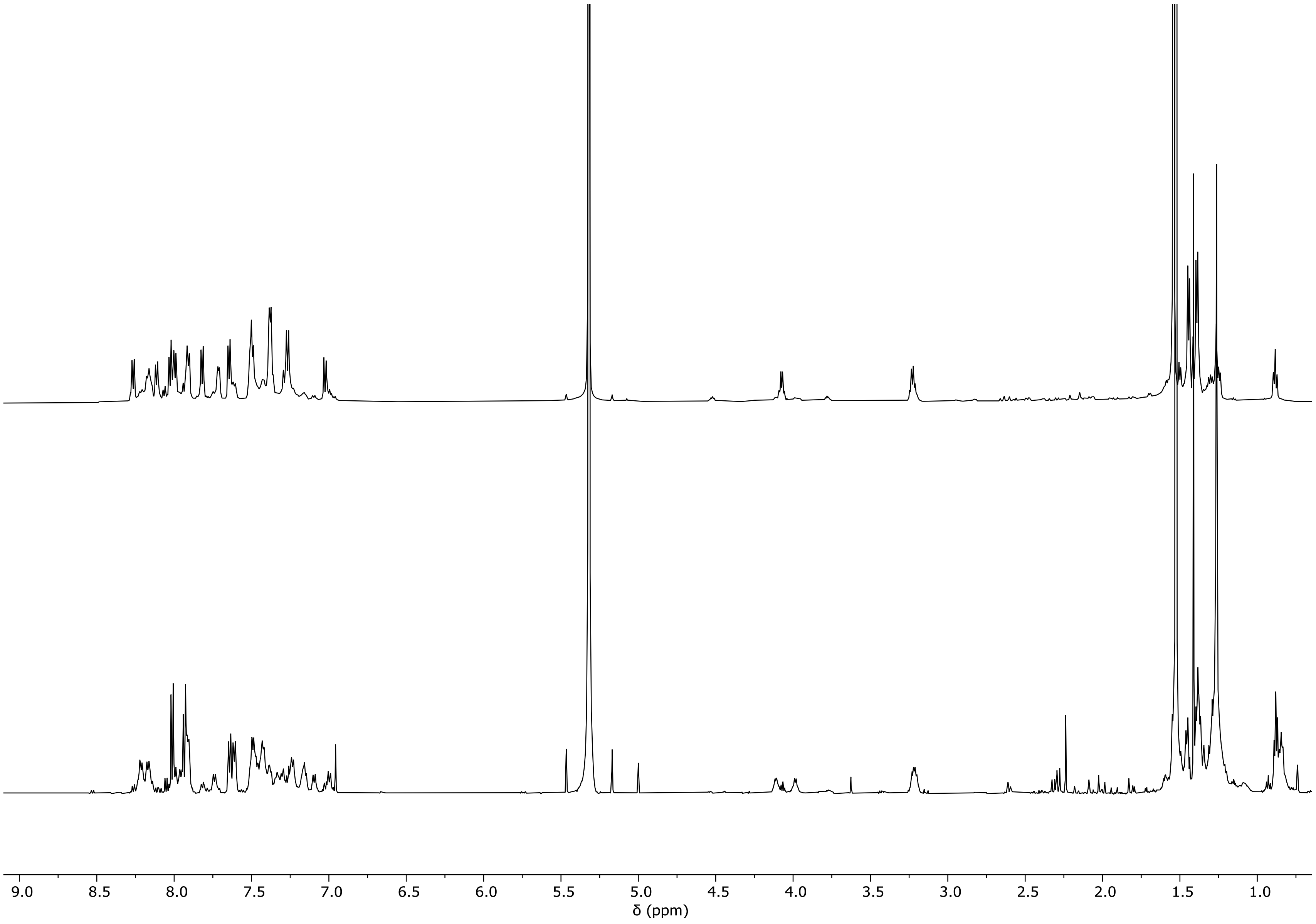

^{1}H-NMR spectrum of *E*-(2*S*,3*S*)**-M1** (top) *E*-(2*S*,3*S*)**-M1** (bottom) (600 MHz, CD_2Cl_2, 25 °C).

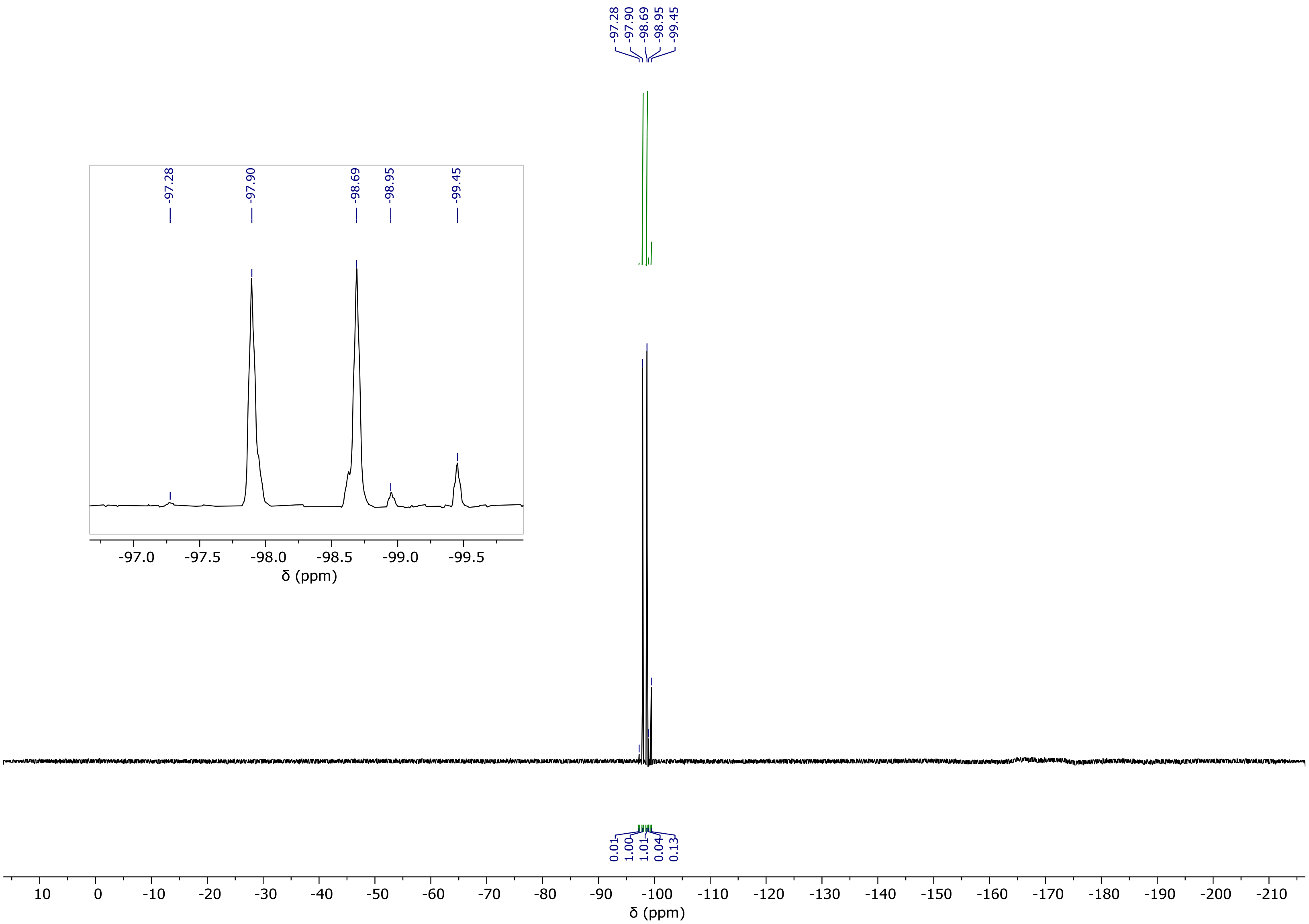

^{19}F-NMR spectrum of an enriched sample of the Z isomer of (2*S*,3*S*)-**M1** (565 MHz, CD_2Cl_2, 25 °C).

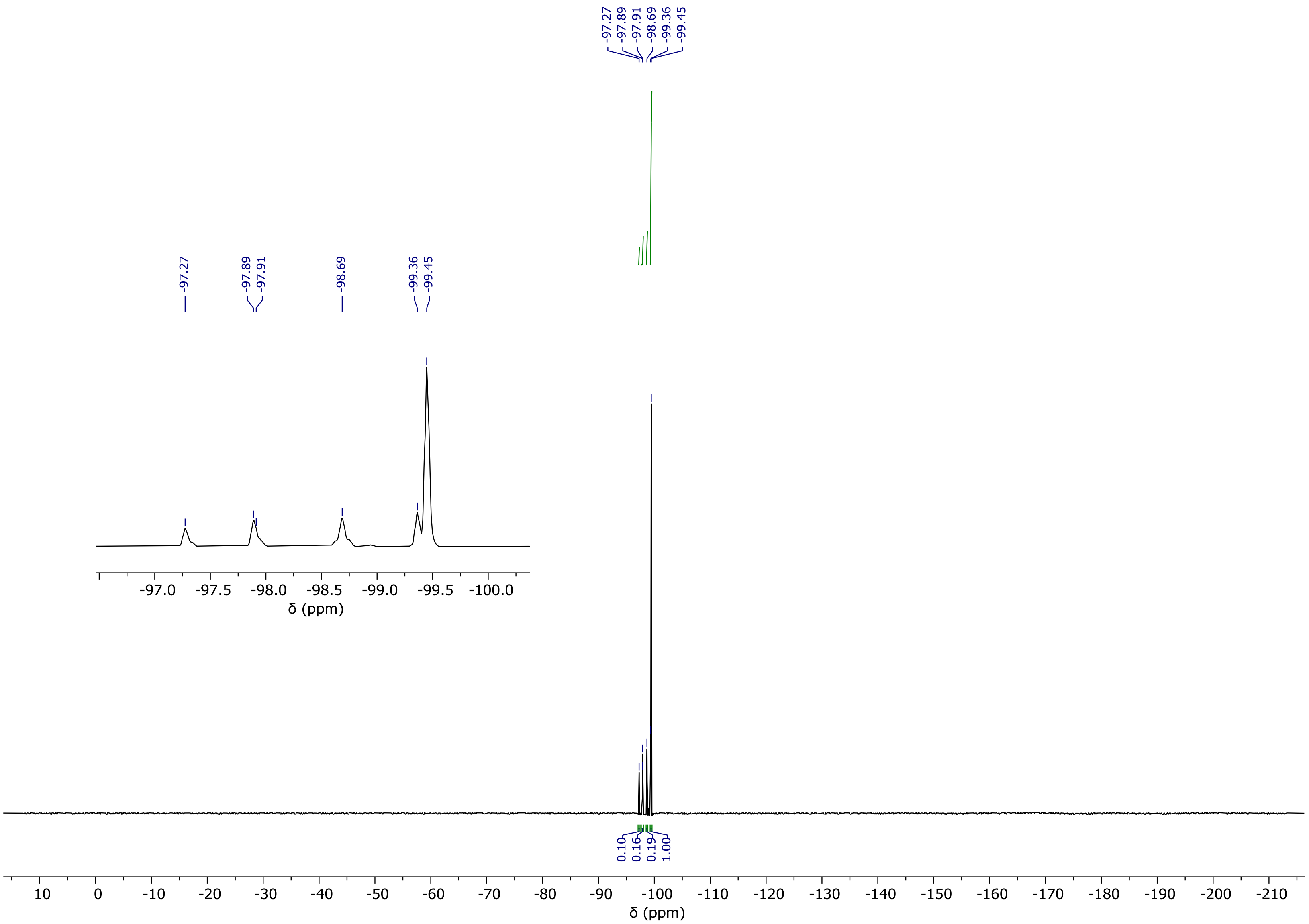

^{19}F-NMR spectrum of an enriched sample of the E isomer of (2*S*,3*S*)-**M1** (565 MHz, CD_2Cl_2, 25 °C).

9. References

[1] P. Nguyen, E. Corpuz, T. M. Heidelbaugh, K. Chow, M. E. Garst, *J. Org. Chem.* **2003**, *68*, 10195–10198.

[2] J.-L. Gras, *Org. Synth.* **1981**, *60*, 88.

[3] T. van Leeuwen, W. Danowski, E. Otten, S. J. Wezenberg, B. L. Feringa, *J. Org. Chem.* **2017**, *82*, 5027–5033.

[4] Y. Gisbert, M. Fellert, C. N. Stindt, A. Gerstner, B. L. Feringa, *J. Am. Chem. Soc.* **2024**, *146*, 12609–12619.

[5] F. T. Bergmann, S. Hoops, B. Klahn, U. Kummer, P. Mendes, J. Pahle, S. Sahle, *J. Biotechnol.* **2017**, *261*, 215–220.

[6] F. Neese, F. Wennmohs, U. Becker, C. Riplinger, *J. Chem. Phys.* **2020**, *152*, 224108.

[7] S. Grimme, A. Hansen, S. Ehlert, J.-M. Mewes, *J. Chem. Phys.* **2021**, *154*, 064103.

[8] V. Barone, M. Cossi, *J. Phys. Chem. A* **1998**, *102*, 1995–2001.

[9] N. A. Farrow, O. Zhang, J. D. Forman-Kay, L. E. Kay, *J. Biomol. NMR* **1994**, *4*, 727–734.

[10] T. van Leeuwen, J. Pol, D. Roke, S. J. Wezenberg, B. L. Feringa, *Org. Lett.* **2017**, *19*, 1402–1405.
